# Anteroposterior patterning of the zebrafish ear through Fgf- and Hh-dependent regulation of *hmx3a* expression

**DOI:** 10.1371/journal.pgen.1008051

**Published:** 2019-04-25

**Authors:** Ryan D. Hartwell, Samantha J. England, Nicholas A. M. Monk, Nicholas J. van Hateren, Sarah Baxendale, Mar Marzo, Katharine E. Lewis, Tanya T. Whitfield

**Affiliations:** 1 Bateson Centre and Department of Biomedical Science, University of Sheffield, Sheffield, United Kingdom; 2 Department of Biology, Syracuse University, Syracuse, New York, United States of America; 3 School of Mathematics and Statistics, University of Sheffield, Sheffield, United Kingdom; University of Washington, UNITED STATES

## Abstract

In the zebrafish, Fgf and Hh signalling assign anterior and posterior identity, respectively, to the poles of the developing ear. Mis-expression of *fgf3* or inhibition of Hh signalling results in double-anterior ears, including ectopic expression of *hmx3a*. To understand how this double-anterior pattern is established, we characterised transcriptional responses in Fgf gain-of-signalling or Hh loss-of-signalling backgrounds. Mis-expression of *fgf3* resulted in rapid expansion of anterior otic markers, refining over time to give the duplicated pattern. Response to Hh inhibition was very different: initial anteroposterior asymmetry was retained, with de novo duplicate expression domains appearing later. We show that Hmx3a is required for normal anterior otic patterning, and that otic patterning defects in *hmx3a*^*-/-*^ mutants are a close phenocopy to those seen in *fgf3*^*-/-*^ mutants. However, neither loss nor gain of *hmx3a* function was sufficient to generate full ear duplications. Using our data to infer a transcriptional regulatory network required for acquisition of otic anterior identity, we can recapitulate both the wild-type and the double-anterior pattern in a mathematical model.

## Author summary

Understanding how signalling molecules impart information to developing organ systems, and how this is interpreted through networks of gene activity, is a key goal of developmental genetic analysis. In the developing zebrafish inner ear, differences in gene expression arise between the anterior and posterior poles of the ear placode, ensuring that sensory structures in the ear develop in their correct positions. If signalling pathways are disrupted, a mirror-image ear can result, developing with two anterior poles. We have used genetic, pharmacological and mathematical modelling approaches to decipher the pathway of gene action required to specify anterior structures in the zebrafish ear. Patterns of gene expression are dynamic and plastic, with two different routes leading to the formation of duplicate anterior structures. Expression of the *hmx3a* gene is an early response to the anterior signalling molecule Fgf3, but is not sufficient to drive the formation of ectopic anterior structures at the posterior of the ear. The *hmx3a* gene codes for a protein that regulates other genes, and in humans, mutation of *HMX* genes results in diseases affecting inner ear function. Our work provides a framework for understanding the dynamics of early patterning events in the developing inner ear.

## Introduction

The otic placode—precursor of the vertebrate inner ear—has the remarkable ability to generate a mirror-image organ with duplicate structures under some experimental conditions in fish and amphibians, as originally described by R. G. Harrison over eighty years ago (reviewed in [1]). Understanding the generation of such duplicated structures can give us fundamental insights into mechanisms of organ patterning, tissue polarity and symmetry-breaking during embryogenesis. During normal development in the zebrafish, anteroposterior asymmetries in otic gene expression are evident as early as the 4-somite stage (11.5 hours post fertilisation (hpf)), when expression of the transcription factor gene *hmx3a* appears at the anterior of the otic placode [2]. Additional genes with predominantly anterior patterns of expression in the otic placode or vesicle begin to be expressed over the next 10 hours, including the transcription factor genes *hmx2* and *pax5* [2, 3], together with the fibroblast growth factor (Fgf) family genes *fgf3*, *fgf8a* and *fgf10a* [4, 5]. Later, at otic vesicle stages (24 hpf onwards), the size and position of the otoliths, together with the position, shape and planar polarity patterns of the sensory maculae, provide landmarks for distinguishing anterior and posterior structures in the ear [6]. In addition, a few markers begin to be expressed specifically in posterior otic tissue (*pou3f3b*, *bmp7a* and *fsta*) at otic vesicle stages [3, 7, 8], but these are not reliable posterior markers at earlier otic placode stages.

Concomitant with the appearance of anteroposterior asymmetry in the zebrafish otic domain, other early patterning events occur that are symmetrical about the anteroposterior axis. Of relevance for our study, a single sensory-competent domain, marked by the expression of *atoh1b*, splits into two domains, one at each pole of the ear, by 12 hpf. This process is dependent on Notch signalling and *atoh1b* function, and defines differences between the poles of the otic placode and a central zone [9]. The two poles express various markers symmetrically, including *atoh1a* and *deltaD*, between 14–18 hpf [9], presaging the appearance of pairs of *myo7aa*-positive sensory hair cells (tether cells) at each pole by 18–24 hpf [10]. Thus, by the completion of otic induction at 14 hpf (10 somites), the otic domain has two clear poles defined by the symmetric expression of *atoh1a* and *deltaD*, with the anterior pole distinguished from the posterior by the asymmetric expression of *hmx3a*.

Although anteroposterior asymmetries in otic gene expression are already apparent by 12 hpf in the zebrafish, these can be disrupted by interfering with extrinsic signalling pathway activity after this time. For example, manipulations of either Fibroblast growth factor (Fgf) or Hedgehog (Hh) signalling between 14–19 hpf can result in striking double-anterior or double-posterior mirror-image ears. Fgf signalling is both required and sufficient to act as an anteriorising cue, whereas Hh signalling is both required and sufficient for the acquisition of posterior otic identity [6, 11, 12]. In these studies, we showed that transient *fgf3* mis-expression at 14 hpf or Hh pathway loss-of-function result in the loss of posterior-specific expression domains of *fsta* at 30 hpf and *otx1b* at 45–48 hpf, and the gain of anterior-specific gene expression in the posterior of the ear (*hmx2* and *pax5* at 24 hpf after *fgf3* mis-expression; *hmx3a* at 30 hpf in Hh loss-of-function mutants). These findings suggest that Fgf and Hh signalling normally act to establish and maintain the asymmetric expression of marker genes within the otic epithelium. However, the details of their temporal mode of action in the duplication of anterior otic fates have not been explored.

In the current study, we have compared the dynamics of the transcriptional responses that precede the acquisition of a duplicated anterior otic fate in an Fgf gain-of-signalling or a Hh loss-of-signalling context. Although the final duplicated ear structures appear similar after each manipulation, the early transcriptional responses differ for each signalling pathway, progressing in distinct ways to give rise to the double-anterior pattern at larval stages. One gene that shows an early transcriptional response in the zebrafish otic placode to disruption of either Fgf or Hh signalling is the *Hmx* family homeobox gene *hmx3a*. We have examined the effects of both loss-of-function and gain-of-function of *hmx3a* on inner ear patterning. Our data suggest that *hmx3a* is a key early target for the otic anteriorising activity of Fgf signalling, and that the function of *hmx3a* is required for the anterior-specific otic expression of *fgf3* and *pax5*, together with correct positioning and development of the sensory maculae. However, unlike high Fgf levels or low Hh pathway activity, mis-expression of *hmx3a* was unable to generate full duplications of anterior character at the posterior of the ear. A mathematical model based on our experimental findings can recapitulate both the wild-type and duplicated anterior pattern, allowing us to explore the dynamical principles underlying the generation of a mirror-image duplicated organ system.

## Results

### Early mis-expression of *fgf3*, but not *fgf8a*, can generate complete double-anterior ear duplications

**Fig 1:**
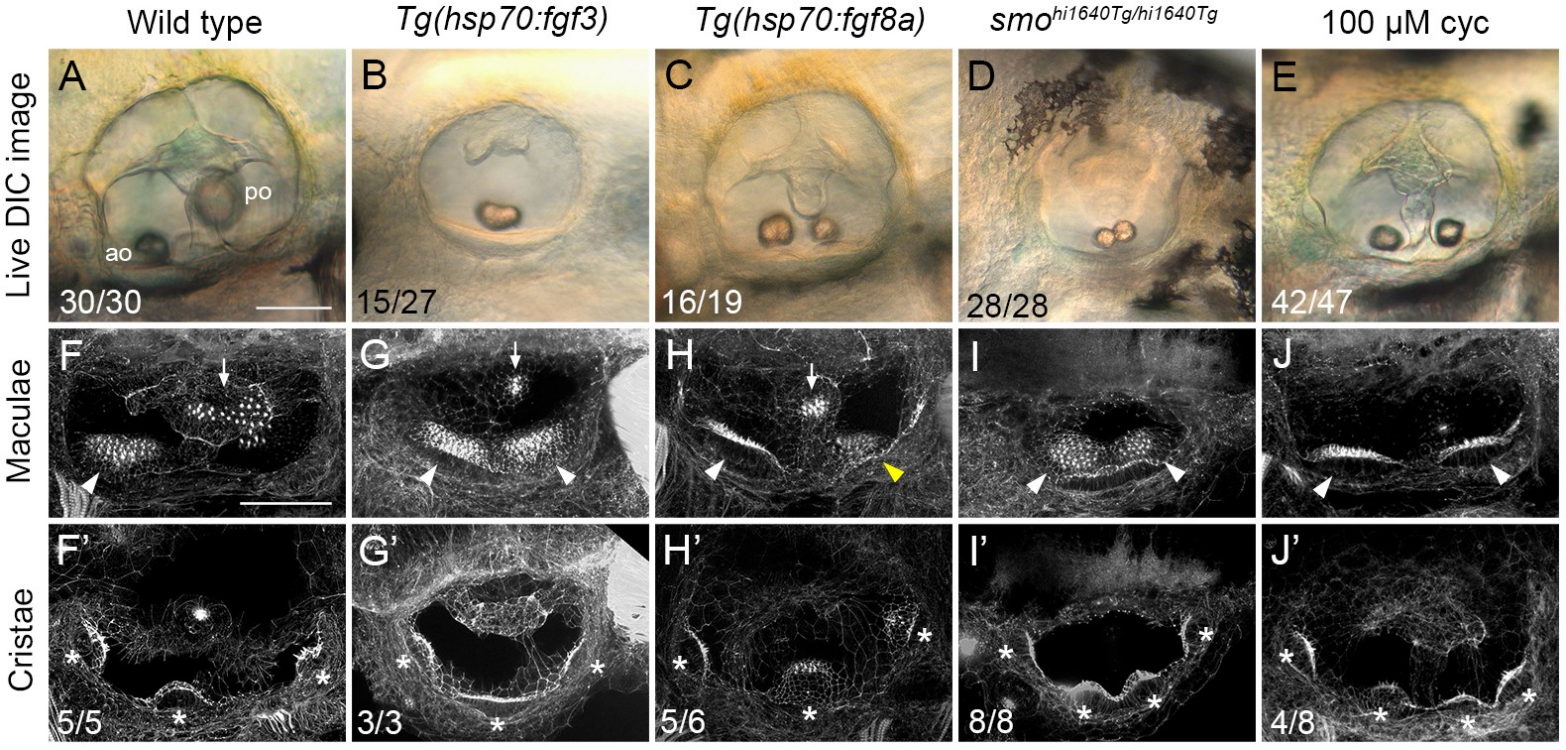
Duplicate double-anterior ear phenotypes resulting from early Fgf mis-expression or Hh pathway inhibition. (A–E) Differential interference contrast (DIC) images of ears in live embryos at 3 dpf (72 hpf). (F–J’) Confocal images of FITC-phalloidin stains, revealing stereociliary bundles on sensory hair cells in the maculae (F–J) or cristae (F’–J’). Anterior maculae and duplicate anterior maculae are marked with arrowheads; posterior maculae and remnants of posterior maculae are marked with arrows. Cristae and duplicate cristae are marked with asterisks. Yellow arrowhead in H indicates macula that is ventromedial in position, and close to remnants of the posterior macula (arrow). Note the enlarged lateral crista in G’. (The bright spot in the centre of F’ is a lateral line neuromast.) Representative phenotypes are shown; numbers of embryos displaying these phenotypes are indicated on the panels. All *Tg(hsp70*:*fgf3)* heat-shocked embryos (*n* = 27) and *smo*^*hi1640Tg/hi1640Tg*^ mutants (*n* = 28) showed double-anterior ears. In B, 15/27 ears had a single fused otolith as shown; the remaining 12/27 ears had two separate, but small and ventrally-positioned otoliths. In C, 16/19 ears showed varying degrees of duplication with two small, ventrolaterally positioned otoliths (14/19, as shown) or fused otoliths (2/19). The remaining 3/19 ears showed a wild-type phenotype. In D, 17/28 ears had two otoliths touching as shown; in the remaining 11/28 ears, the otoliths were separate, but both small and ventrally positioned. Genotypes or treatments are indicated for each column. Transgenic lines were subject to 30 minutes of heat shock at 14 hpf. Additional controls for this figure are shown in S1 Fig. Lateral views; anterior to the left. Abbreviations: ao, anterior (utricular) otolith; po, posterior (saccular) otolith; cyc, cyclopamine. Scale bar in A, 50 μm (applies to A–E); scale bar in F, 50 μm (applies to F–J’).

To establish optimal conditions for generating a double-anterior ear in the zebrafish embryo, we compared otic phenotypes in transgenic lines for two different *fgf* genes, *fgf3* and *fgf8a*, with systemic transgene expression driven under the control of the *hsp70* heat-shock promoter [13, 14]. Previously, we showed that a 2-hour heat shock in the *Tg(hsp70*:*fgf3)* line at 14 hpf (10-somite stage) resulted in a robust duplication of anterior otic structures [6]. We chose this time point to avoid any interference with otic placode induction, which is also Fgf-dependent, but is complete by 14 hpf [15, 16]. The 14 hpf time point also follows completion of the Notch-dependent signalling event that distinguishes the otic poles from a central zone of epithelium [9]. For the treatments described here, we reduced the time of heat shock to 30 minutes at 39°C. This shorter heat shock still results in a full ear duplication, but should minimise effects of Fgf mis-expression on other developing organ systems. After heat shock, embryos were then cultured at 33°C for a further 30 minutes, to reduce the incubation temperature gradually, before being returned to 28.5°C and incubated until 3 days post fertilisation (dpf) for processing and analysis (Fig 1). This stepwise reduction in temperature is thought to extend transgene activation and reduce cell death following heat shock [17, 18]. Non-transgenic sibling embryos, subjected to the same heat-shock treatment, served as controls (S1 Fig).

In *Tg(hsp70*:*fgf3)* embryos, a 30-minute heat shock at 14 hpf gave a robust and complete duplication of anterior otic patterning at 72 hpf, as indicated by the mis-positioning or fusion of the posterior otolith, loss of posterior elements of the saccular macula, and a duplication of anterior (utricular-like) sensory elements on the posteroventral floor of the ear (Fig 1B, 1G and 1G’). The phenotypes seen after mis-expression of *fgf8a* (30-minute heat shock at 14 hpf) were milder and more variable than those for *fgf3*, and included a split and mis-positioned saccular macula rather than a complete duplication of anterior elements (Fig 1C and 1H), and a normal complement of three cristae (Fig 1H’). A 30-minute heat shock of either transgenic line at a later stage (18 hpf) resulted in only mild effects on ear size and shape, and otolith position (S2A–S2D Fig) [6]. We therefore chose to use the *Tg(hsp70*:*fgf3)* line, with a 30-minute heat shock (39°C) at 14 hpf followed by 30 minutes at 33°C, in subsequent heat-shock experiments.

### Genetic or pharmacological inhibition of Hh signalling can also result in complete double-anterior ear duplications

To optimise our protocols for generating double-anterior duplicated ears through inhibition of Hh signalling, we first examined the ear phenotype in *smo*^*hi1640Tg/hi1640Tg*^ mutants. The *hi1640Tg* allele (a transgenic insertion in the *smoothened* gene, and a likely null [19]) is thought to result in a stronger reduction in Hh signalling than the point mutation alleles *smo*^*b641*^ and *smo*^*b577*^, both of which predict single amino acid substitutions [20], and which we used in previous studies [6, 11]. The *smo*^*hi1640Tg/hi1640Tg*^ mutants showed a fully penetrant double-anterior duplicated ear phenotype, with two similar-sized small otoliths located ventrally, complete loss of the posterior (saccular) macula, and duplication of the anterior (utricular) macula at the posterior of the ear, with anterior and posterior elements sometimes present as a contiguous patch of hair cells covering the ventral floor (Fig 1D and 1I). Four cristae, rather than the usual three, were present in all (8/8) mutant ears imaged (Fig 1I’). (For comparison, four cristae were present in only about 50% of ears of *smo*^*b641/b641*^ mutant embryos [11].)

Pharmacological inhibition of the transducer of the Hh pathway, Smoothened, using the small molecule cyclopamine, can also produce double-anterior ear duplications [12, 21]. This approach enables a conditional inhibition of Hh signalling over a defined time window. For the experiments described here, we treated wild-type embryos with 100 μM cyclopamine from 14–22.5 hpf. To examine later stages, we washed out the cyclopamine at 22.5 hpf and allowed embryos to develop further until 3 dpf (72 hpf), when they were fixed for staining and imaging. Stage-matched sibling embryos—either untreated, or treated with vehicle (ethanol) only—served as controls. This cyclopamine treatment regime was sufficient to generate the double-anterior ear phenotype, characterised by two ventrally-positioned, small (utricular-like) otoliths, loss of the posterior (saccular) macula, and a clear duplication of the anterior (utricular) macula (Fig 1E and 1J). Ears in 4/8 treated embryos had four cristae (Fig 1J’); the remaining 4/8 had the normal complement of three cristae. The size and shape of the ear were less affected than in the *smo*^*hi1640Tg/hi1640Tg*^ mutant embryos, presumably due to the transient nature of the cyclopamine treatment. Taken together, these data show that either genetic or pharmacological inhibition of Hh signalling in wild-type zebrafish embryos between 14–22.5 hpf results in a robust and reproducible double-anterior ear phenotype at 3 dpf.

### Following early *fgf3* mis-expression, otic expression of anterior markers is initially broad, with *pax5* resolving into two discrete domains

One of the most striking transcriptional changes in response to *fgf3* mis-expression is the expansion or duplication of the expression of the anterior otic markers *hmx2* and *pax5* by 24 hpf [6]. To examine the temporal dynamics of this transcriptional response, we assayed for expression of these and additional anterior otic marker genes following our optimised ‘early’ heat-shock regime (14 hpf, 30 min, 39°C) at three different time points: 16 hpf (2 hours post heat shock, to examine any rapid response), 22.5 hpf (8.5 hours post heat shock, when anterior otic expression of *hmx2* and *pax5* is strongly established in wild-type embryos) and 36 hpf (22 hours post heat shock, to examine whether any disruption to the expression pattern resolves or changes over time). For *hmx2* and *pax5*, which showed dynamic expression changes, we subsequently included two additional time points (25.5 hpf and 30 hpf) to capture these changes in more detail.

**Fig 2:**
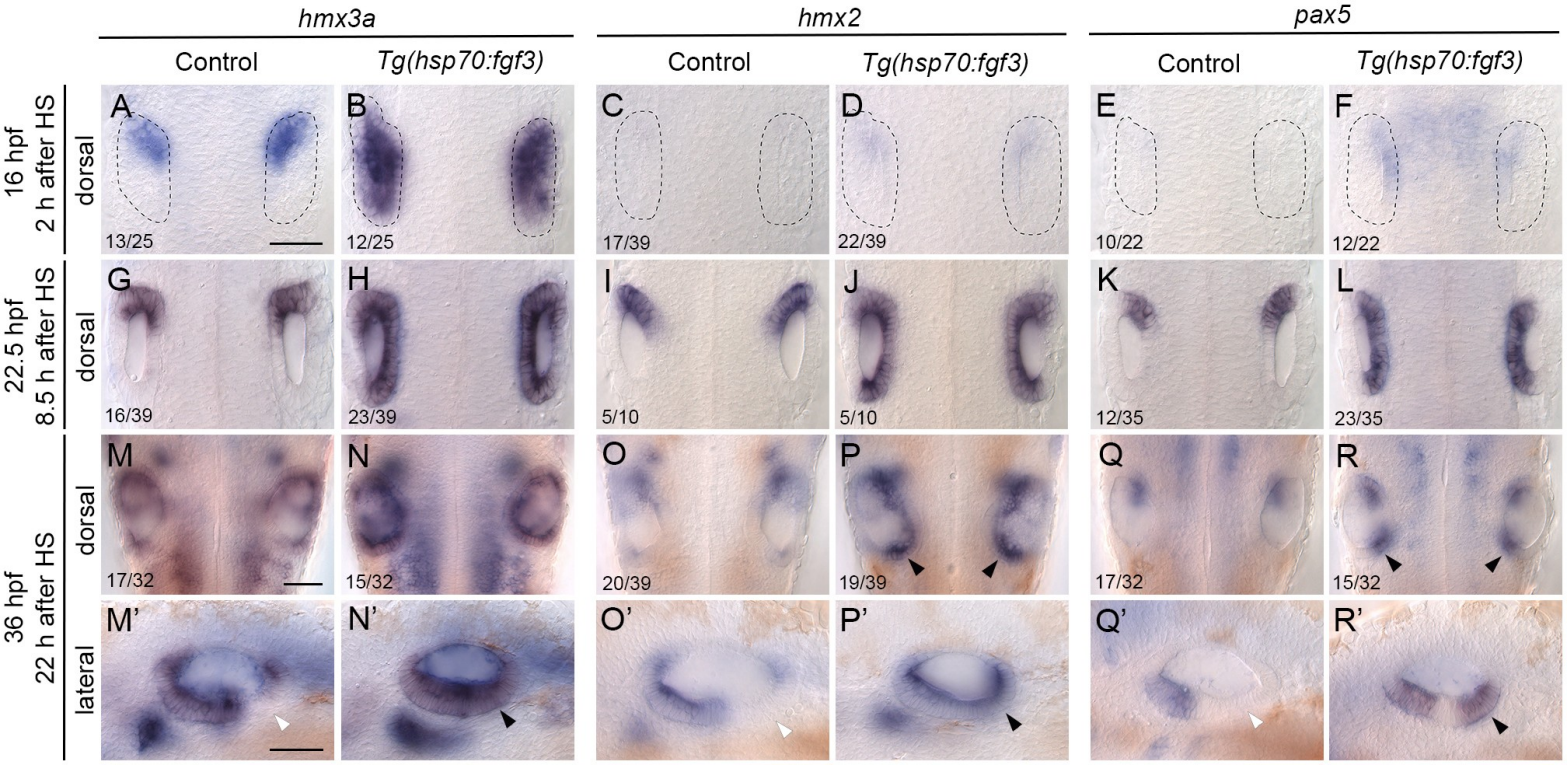
Expression of the otic anterior marker genes *hmx3a*, *hmx2* and *pax5* after early *fgf3* mis-expression. In situ hybridisation of otic expression patterns in *Tg(hsp70*:*fgf3)* embryos following a 30-minute heat shock (HS) at the 10-somite stage (14 hpf). Controls (left-hand panels of each pair of images) were sibling non-transgenic embryos subjected to the same heat shock. Numbers in the dorsal view panels indicate the number of embryos with the phenotype shown and total number (e.g. 13/25) from a mixed batch of transgenic and non-transgenic embryos in each pair of panels; 50% of the batch was expected to be transgenic. (A–F) Two hours after heat shock (16 hpf), expression of *hmx3a* expanded to cover the entire otic region (B), but there was only a trace of expression of *hmx2* or *pax5* in the otic placode at this stage. Weak expression of *pax5* in the hindbrain after heat shock (F) did not persist (L). (G–L) At 22.5 hpf (8.5 hours after HS), expression of all three genes had now expanded to cover the entire anteroposterior axis of the otic vesicle on the medial side. (M–R’) At 36 hpf (22 hours after HS), expression of *hmx3a* remained expanded across the otic anteroposterior axis (N,N’); expression of *hmx2* was strong at the anterior and posterior poles, and weaker in central regions (P,P’), whereas expression of *pax5* resolved into two discrete domains at the anterior and posterior poles of the otic vesicle, and was lost from central regions (R,R’). White arrowheads indicate regions that are normally free of expression in controls; black arrowheads mark ectopic expression in transgenic embryos. A–R are dorsal views showing both otic vesicles, with anterior to the top; M’–R’ are lateral views with anterior to the left. Scale bars, 50 μm (scale bar in A applies to A–L; in M applies to M–R; in M’ applies to M’–R’). For additional examples and time points for *hmx2*, see S4 Fig; for *pax5*, see S5 Fig.

We first tested expression of three genes coding for transcription factors (*hmx3a*, *hmx2* and *pax5*; Fig 2). At the earliest time point (16 hpf, two hours after heat shock), *hmx3a* showed the strongest response: expression had already expanded to cover the entire anteroposterior extent of the otic placode (Fig 2A and 2B). The anterior markers *hmx2* and *pax5*, not normally expressed at this stage in wild-type embryos, were expressed at very low levels in the anterior of the otic placode of heat-shocked embryos (Fig 2C–2F). We were also able to detect widespread and robust up-regulation of the Fgf target gene *etv4* (formerly *pea3*) in transgenic embryos at this time point (S3 Fig). By 22.5 hpf, all three transcription factor genes were strongly expressed in a broad zone across the entire anteroposterior axis of the otic vesicle in heat-shocked embryos, on the medial side, as can be seen in a dorsal view (Fig 2G–2L). Note that the overall size and shape of these otic vesicles were relatively normal. Although there was some variability (including between both ears of the same fish), the vesicles were oval in shape, indicating that otic induction had not been compromised (compare with the small, rounded vesicles of *fgf8a*^*ti282/ti282*^ mutants, in which otic induction is disrupted [4]). By 36 hpf, wild-type otic expression of *hmx* genes was more complex, but a clear difference between anterior expressing and posterior non-expressing regions was evident in ventral otic epithelium (Fig 2M, 2M’, 2O and 2O’). By contrast, in heat-shocked embryos, expression of *hmx3a* remained strong across the entire anteroposterior axis of the ear in ventral regions (Fig 2N and 2N’); expression of *hmx2* weakened in central regions during intermediate stages, but at 36 hpf was present in a contiguous ventral domain (Fig 2P and 2P’; S4 Fig), while expression of *pax5* was lost in central regions, resolving into two discrete ventral domains at the anterior and posterior poles by 25.5 hpf (Fig 2R and 2R’; S5 Fig).

To test whether the milder ear phenotype caused by a later heat shock reflects a failure to establish the early transcriptional responses described above, we also examined expression of anterior markers in *Tg(hsp70*:*fgf3)* embryos after heat shock at 18 hpf (30 min, 39°C). Unexpectedly, we found that the otic expression of *hmx3a* and *pax5* was very similar to that following an early (14 hpf) heat shock, with a broad band of ectopic expression extending across the entire anteroposterior axis of the otic vesicle by 22.5 hpf (S2E–S2H’ Fig). This suggests that the loss of competence to generate a complete double-anterior ear after a late heat shock is not due to an inability to express *hmx3a* and *pax5* ectopically throughout the otic epithelium at otic vesicle stages.

### Inhibition of Hh signalling results in a slower and spatially distinct transcriptional response in the otic vesicle

**Fig 3:**
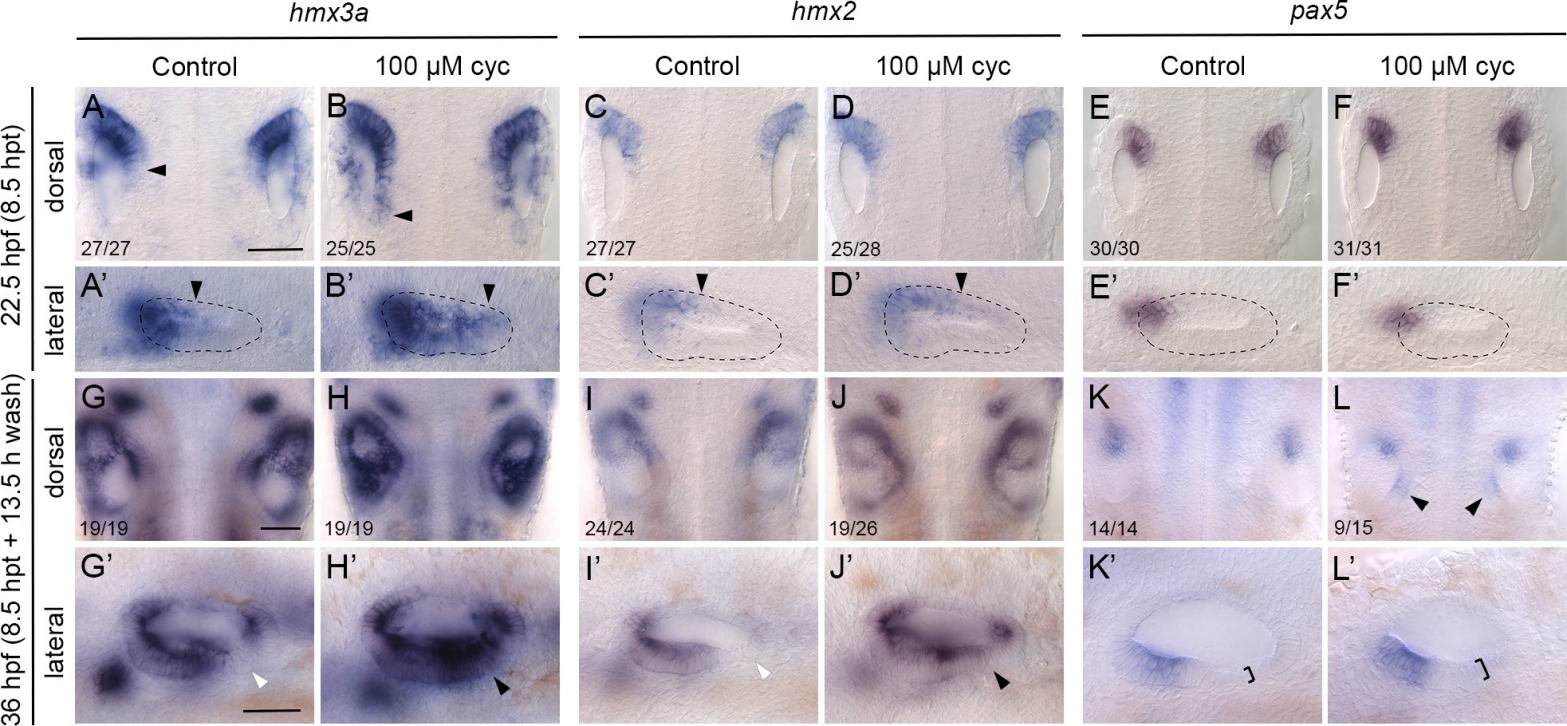
Expression of the otic anterior marker genes *hmx3a*, *hmx2* and *pax5* after Hh pathway inhibition. Expression of mRNA for anterior otic markers in embryos treated with 100 μM cyclopamine (cyc) from the 10-somite stage (14 hpf) until 22.5 hpf. Controls in the left-hand panels of each pair of images were treated with vehicle (ethanol) only. (A–F’) At 22.5 hpf (8.5 hours post initiation of treatment, hpt), expression of *hmx3a* expanded into posterior regions of the otic vesicle (arrowheads); expression of *hmx2* showed a modest expansion and there was no change in the otic expression pattern of *pax5*. Arrowheads in A–D’ indicate posterior extent of otic expression. (G–L’) At 36 hpf (8.5 hpt + 13.5 h wash), expression of both *hmx3a* and *hmx2* extended into posteroventral regions of the otic epithelium. White arrowheads indicate regions that are normally free of expression in controls; black arrowheads mark ectopic expression in cyclopamine-treated embryos. By 36 hpf, expression of *pax5* appeared in a new discrete domain in posteromedial otic epithelium after cyclopamine treatment (L, arrowheads); in a lateral view, the epithelium in posterolateral regions was thicker than normal (K’,L’, brackets). A–L are dorsal views showing both otic vesicles, with anterior to the top; A’–L’ are lateral views with anterior to the left. Scale bars, 50 μm (scale bar in A applies to A–F’; in G applies to G–L; in G’ applies to G’–L’).

To compare the transcriptional response after *fgf3* mis-expression at 14 hpf with that following conditional Hh pathway inhibition from the same time point, we examined otic expression of anterior marker genes after treatment of wild-type embryos with cyclopamine (100 μM, 14–22.5 hpf; Fig 3). To confirm the efficacy of cyclopamine treatment, we also examined the expression of *ptch2*, a known target of Hh signalling. Expression of *ptch2* was down-regulated throughout the embryo at 22.5 hpf, but not abolished (S6 Fig). (By contrast, *ptch2* expression is almost entirely lost at 24 hpf in *smo*^*hi1640Tg/hi1640Tg*^ mutants [19]). We also checked expression of *etv4* following cyclopamine treatment, but found no major changes in expression at 22.5 hpf (S6 Fig). This result confirmed that there are no strong direct effects of the transient pharmacological inhibition of the Hh pathway on Fgf signalling activity in the ear, in line with our previous findings after genetic abrogation of Hh signalling [6].

We examined otic marker genes at two different time points following cyclopamine treatment (22.5 hpf and 36 hpf). Otic expression of both *hmx3a* and *hmx2* was expanded posteriorly on the medial side of the otic vesicle at 22.5 hpf, 8.5 hours after the start of the treatment (Fig 3A–3D’; S7 Fig). Expanded otic *hmx* gene expression was also present by 23 hpf in the *smo*^*hi1640Tg/hi1640Tg*^ mutant, in which the Hh pathway is constitutively inactive (S7 Fig). Importantly, there was no significant difference in the expansion of *hmx3a* expression in the ear at 22.5–23 hpf between the *smo* mutants and cyclopamine-treated embryos, indicating that our cyclopamine treatment regime is effective at suppressing Hh signalling relevant to otic patterning at this stage (S7 Fig).

The spatial pattern of *hmx* expansion in response to Hh inhibition was different to that seen after mis-expression of *fgf3*. Specifically, in cyclopamine-treated embryos or *smo*^*hi1640Tg/hi1640Tg*^ mutants, *hmx3a* and *hmx2* were expressed in a graded fashion across the ear at 22.5 hpf (8.5 hours after treatment), with higher levels anteriorly, rather than in a uniform broad band (compare Fig 3B with Fig 2H). To examine later time points, treated embryos were transferred to fresh medium at 22.5 hpf without cyclopamine, as described above. By 36 hpf (13.5 hours post wash), expression of *hmx* genes had expanded further posteriorly to cover most of the ventral floor of the otic vesicle in cyclopamine-treated embryos (Fig 3G–3J’), as observed previously at 30 hpf in *con*^*tf18b/tf18b*^ and *smo*^*b641/b641*^ mutants, both of which have a strong reduction in Hh signalling [11].

Expression of *pax5* was slower to respond following cyclopamine-mediated inhibition of Hh signalling, with no apparent expansion of the expression domain within the otic vesicle at 22.5 hpf (Fig 3E–3F’). These results corroborate our previous observations in *con*^*tf18b/tf18b*^ and *smo*^*b641/b641*^ mutants, where there was little change in the otic expression of *pax5* at 24 hpf [11]. However, by the later time point (36 hpf; 13.5 hours post wash), a new, discrete domain of *pax5* expression appeared within posteromedial otic epithelium of cyclopamine-treated embryos (Fig 3K and 3L). Anteroposterior asymmetry in treated ears was still evident at this stage: the posterior domain of expression was weaker, and in a more medial position, than the anterior expression domain (Fig 3K and 3L). However, the epithelium in posteroventral regions was thicker than normal, indicating development of a duplicate domain of sensory tissue (Fig 3K’ and 3L’). Taken together, our data indicate that the duplicated anterior domain resulting from either an Fgf gain-of-function or Hh loss-of-function includes a duplication of *pax5* expression, but that otic patterning progresses through completely different intermediate states to achieve this duplicated pattern, depending on the signalling pathway that has been disrupted.

### Expression of Fgf family genes in the otic epithelium following *fgf3* mis-expression or Hh inhibition

**Fig 4:**
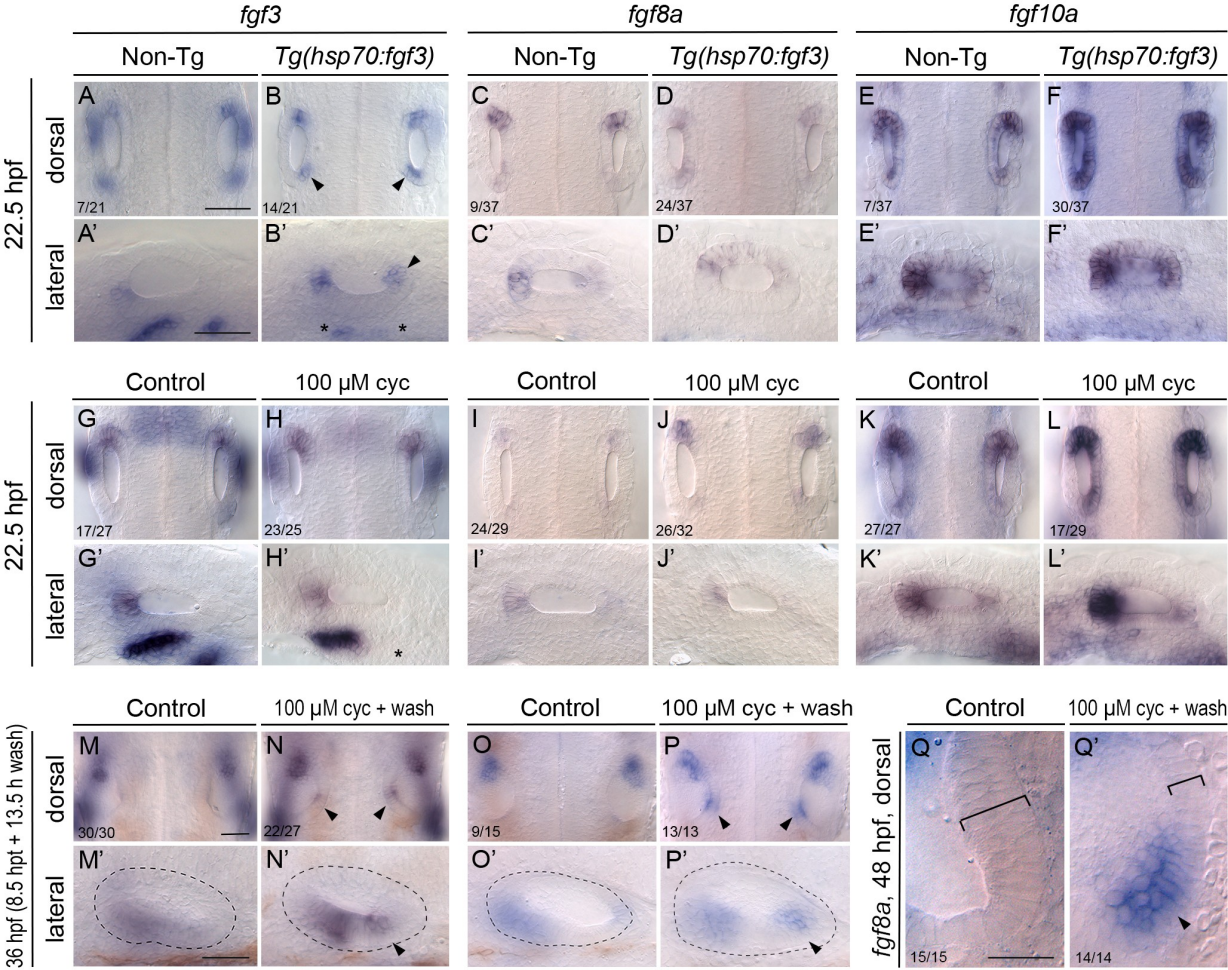
Otic expression of *fgf* genes following mis-expression of *fgf3* or inhibition of Hh signalling. (A–F’) In situ hybridisation for otic expression of *fgf* genes in *Tg(hsp70*:*fgf3)* embryos following a 30-minute heat shock (HS) at the 10-somite stage (14 hpf). Controls (left-hand panels of each pair of images) were sibling non-transgenic (Non-Tg) embryos subjected to the same heat shock. Numbers of embryos shown in the dorsal view panels indicate the number showing the phenotype from a mixed batch of transgenic and non-transgenic embryos in each pair of panels; 75% of the batch is expected to be transgenic. A–B’ show staining with a probe specific to the 3’ UTR of *fgf3*: note the ectopic patch of endogenous *fgf3* expression at the posterior otic pole (B,B’; arrowheads) and disruption to *fgf3* expression ventral to the otic vesicle (B’; asterisks) after heat shock in transgenic embryos. Expression of *fgf10a* is strengthened in the otic vesicle of transgenic embryos after heat shock (E–F’). (G–Q’) Expression of mRNA for *fgf* genes in embryos treated with 100 μM cyclopamine (cyc) from the 10-somite stage (14 hpf) until 22.5 hpf. Controls in the left-hand panels of each pair of images were treated with vehicle (ethanol) only. Numbers of embryos with the phenotype shown for individual treatments are indicated in the dorsal view panels. There was little change to the otic expression patterns of *fgf3* or *fgf8a* at 22.5 hpf (8.5 hours post treatment) (G–J’), but note the loss of *fgf3* expression ventral to the ear (H’; asterisk). Expression of *fgf10a* in the otic vesicle was strengthened after inhibition of Hh signalling in about 50% of treated embryos (L,L’). At 36 hpf (8.5 hpt + 13.5 h wash), ectopic expression of both *fgf3* and *fgf8a* appeared in a new posteromedial domain in the ears of cyclopamine-treated embryos (M-P’; arrowheads). (Q,Q’) Expression of *fgf8a* in the posterior of the otic vesicle at 48 hpf (8.5 hpt + 25.5 h wash). Ectopic expression has strengthened (arrowhead) and medial epithelium is thinner than normal (brackets). Dorsal views of the left ear, with anterior to the top. Scale bar in A, 50 μm (applies to A–L); scale bar in A’, 50 μm (applies to A’–L’); scale bar in M, 50 μm (applies to M–P); scale bar in M’, 50 μm (applies to M’–P’); scale bar in Q, 20 μm (applies to Q,Q’).

Expression of *fgf3* is itself a marker of anterior otic epithelium from 21 hpf [9], and so can also be used to indicate the presence of a duplicated anterior otic pattern. We therefore examined the expression of *fgf* genes to provide additional confirmation of anterior character in the duplicated ears. To distinguish between expression of the *fgf3* transgene and endogenous *fgf3* expression, we used a probe generated from the *fgf3* 3’ UTR, which is not included in the transgenic construct. In *Tg(hsp70*:*fgf3)* embryos after early (14 hpf) heat shock, expression of endogenous *fgf3* now appeared in a new domain at the posterior of the otic vesicle at 22.5 hpf (Fig 4A–4B’, arrowheads). Importantly, expression was not found across the entire anteroposterior axis, but was only present at the poles. Expression of endogenous *fgf3* in pharyngeal endoderm beneath the ear was reduced or missing in heat-shocked transgenic embryos (Fig 4A–4B’, asterisks). We also examined the otic expression of *fgf8a* and *fgf10a* (Fig 4C–4F’). These genes are also normally expressed in the anterior of the otic vesicle, but show a less restricted pattern of expression than that of *fgf3*, with weaker expression also normally found in posterior regions [4, 5, 22]. Following early heat shock of *Tg(hsp70*:*fgf3)* embryos, there was little change in the expression of *fgf8a* in the otic epithelium, whereas expression of *fgf10a* was strengthened at both anterior and posterior poles (Fig 4C–4F’).

We also examined the otic expression of *fgf* genes after pharmacological inhibition of Hh signalling. At 22.5 hpf, following cyclopamine treatment from 14 hpf, there was little change in the expression domain or levels of *fgf3* or *fgf8a* in the otic epithelium (Fig 4G–4J’), although there was loss of an *fgf3* expression domain in pharyngeal pouch endoderm ventral to the ear (Fig 4H’, asterisk). Otic expression of *fgf10a* was strengthened in about 50% (17/29) of cyclopamine-treated embryos at this early time point, especially at the anterior otic pole (Fig 4K–4L’). At 36 hpf (13.5 hours after cyclopamine wash-out), new, discrete domains of *fgf3* and *fgf8a* had appeared at the posterior of the ear, indicating a duplication of anterior otic character (Fig 4M–4P’, arrowheads). By 48 hpf, the duplicated expression domain of *fgf8a* persisted (Fig 4Q’, arrowhead), and loss of the thickened epithelium characteristic of the posterior macula on the medial wall of the otic vesicle was also apparent (Fig 4Q and 4Q’, brackets). These data demonstrate that in both Fgf gain-of-function and Hh loss-of-function contexts, the duplicated anterior otic character includes expression of *fgf* genes.

Note that the double-anterior ear phenotypes resulting from *fgf3* mis-expression or Hh inhibition differ from those resulting from a loss of Notch signalling. In *mindbomb1*^*ta52b/ta52b*^ *(mib1)* mutants, in which the *ta52b* mutation is known to have strong antimorphic effects on Notch signalling [23], an expanded expression domain of *atoh1* genes marks a sensory-competent area in the otic epithelium at 14 hpf [9]. Hair cells differentiate precociously and in excess; supporting cells are missing; otoliths are small and fail to biomineralise correctly; and supernumerary hair cells are extruded from the ear by 60 hpf [24, 25]. By contrast, in the ears of embryos that have undergone *fgf3* mis-expression or Hh inhibition from 14 hpf, we do not find any evidence for supernumerary or precocious hair cell production, loss of supporting cells, or extrusion of hair cells from the epithelium. Size and shape of otoliths is affected, but they appear to become mineralised normally, unlike in *mib1* mutants. The *mib1*^*ta52b/ta52b*^ mutant phenotype is therefore quite different to the phenotypes we have described here. To confirm this, we have examined the expression of *hmx3a*, *fgf3*, *fgf8a* and *pax5* in *mib1*^*ta52b/ta52b*^ mutants (S8 Fig). We found no evidence for any expansion of *hmx3a* or *pax5* expression, posterior duplication of *fgf3*, or posterior upregulation of *fgf8a* expression. By contrast, anteroposterior asymmetry in otic expression was retained for all four genes, but all were in fact down-regulated at the anterior pole of the otic vesicle. Expression of *fgf3* in pharyngeal endoderm beneath the ear was also unaffected in *mib1*^*ta52b/ta52b*^ mutants (S8F’ Fig), in contrast to the effects of *fgf3* mis-expression (Fig 4B’) or Hh inhibition (Fig 4H’).

### Loss of *hmx3a* function results in a fusion of sensory maculae and otoliths, and a reduction in anterior otic character

**Fig 5:**
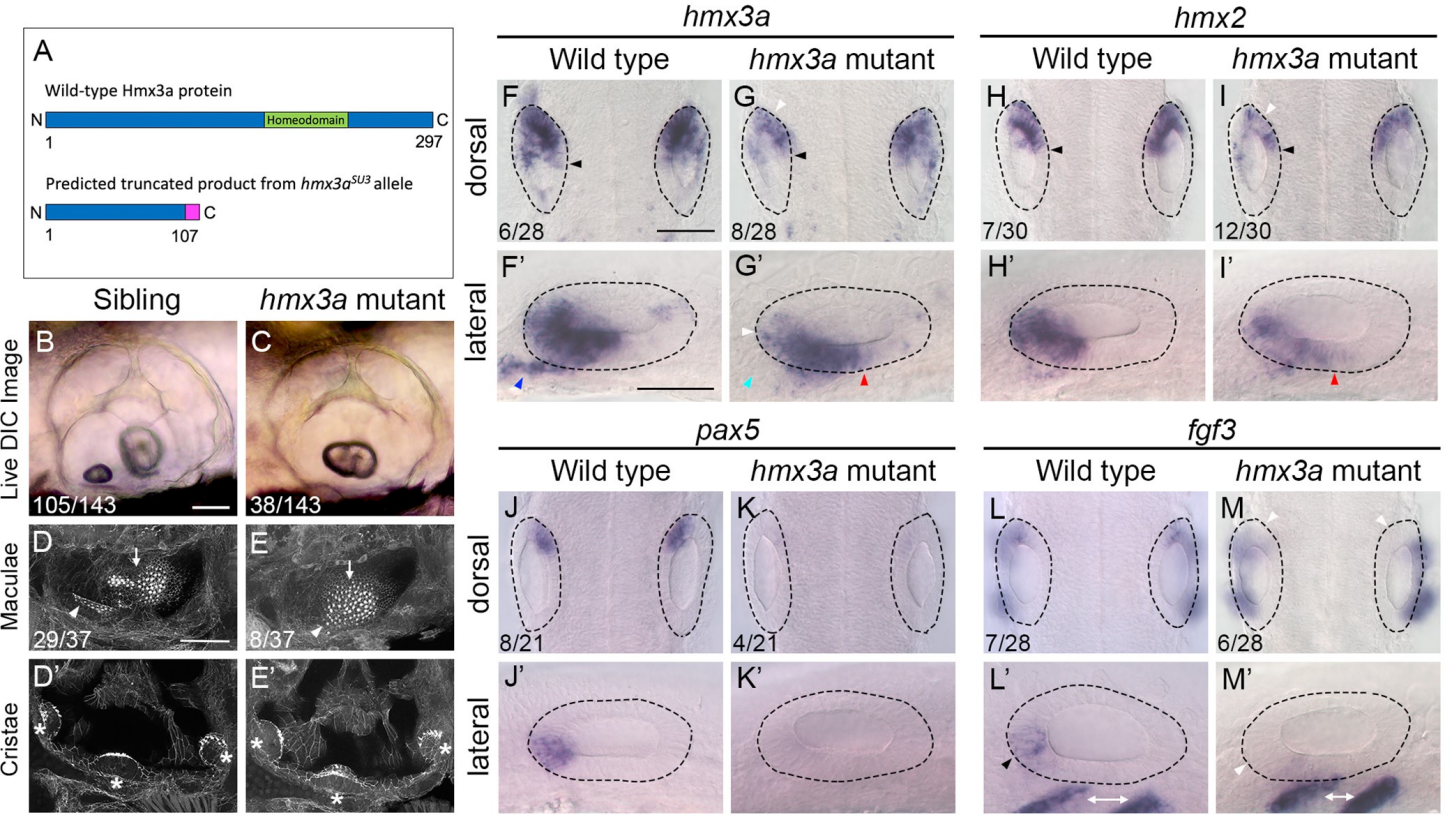
Fused otoliths and sensory maculae, and reduction of anterior otic character, in *hmx3a*^*SU3/SU3*^ mutants. (A) Schematic diagram showing the predicted truncated product for the *hmx3a*^*SU3*^ allele. The mutation was generated using a CRISPR sgRNA targeting sequence in exon 2 upstream of the DNA-binding homeodomain (green). The predicted truncated protein produced by the *hmx3a*^*SU3/SU3*^ allele contains a Thr to Gly substitution at amino acid 107, followed by a stretch of 10 further incorrect amino acids (magenta). The truncated protein lacks the homeodomain. (B,C) Differential interference contrast (DIC) images of ears in live embryos at 3 dpf (72 hpf). Numbers of embryos in a batch from a mating between heterozygous parents are given. Note the fused otolith in the *hmx3a*^*SU3/SU3*^ mutant ear (C). (D–E’) FITC-phalloidin stains of the sensory maculae (D,E) and cristae (D’,E’) in the ear at 3 dpf (72 hpf). Numbers of embryos showing the phenotype from a cross between heterozygous parents are shown. White arrowhead: anterior macula; white arrow: posterior macula; asterisks indicate cristae. Additional examples are shown in S9 Fig. (F–M’) In situ hybridisation for otic anterior markers at 24 hpf in genotyped wild-type and *hmx3a*^*SU3/SU3*^ mutant embryos. The dotted outline marks the outer edge of the otic epithelium. Black arrowheads in F–I indicate the extent of *hmx* expression in medial epithelium; white arrowheads indicate areas of reduced expression levels; blue arrowhead in F’ marks presumed otic or anterior lateral line neuroblasts; light blue arrowhead in G’ indicates loss of expression in this area; red arrowheads in G’,I’ mark expansion of expression in ventral otic epithelium. Black arrowhead in L’ indicates anterior otic expression domain of *fgf3*, lost in M,M’ (white arrowheads); white double-headed arrows mark expression of *fgf3* in pharyngeal pouch endoderm. Numbers in panels F–M indicate numbers of embryos genotyped as either wild type or homozygous mutant that showed the representative expression patterns illustrated. Scale bars, 50 μm (scale bar in B applies to B,C; scale bar in D applies to D–E’; scale bar in F applies to F–I, J–M; scale bar in F’ applies to F’–I’, J’–M’).

Given the early anterior-specific otic expression of *hmx3a* [2], the dependence of this expression on Fgf signalling [6, 26, 27], and the rapid change in otic *hmx3a* expression after mis-expression of *fgf3* or Hh inhibition (this work), we hypothesised that *hmx3a* is required for normal otic anterior development. A previous study using morpholino-mediated knockdown suggested a requirement for both *hmx3a* and *hmx2* in acquisition of anterior otic identity and expression of *pax5* [2]. However, the effects of individual gene knockdown or mutation were not reported. To test the individual requirement for *hmx3a* function in the acquisition of otic anterior identity, we examined the ear phenotype in homozygous mutants for a recessive truncating allele lacking the homeodomain, *hmx3a*^*SU3*^, which we generated using CRISPR/Cas9 technology (Fig 5A; Materials and Methods). In homozygous *hmx3a*^*SU3/SU3*^ mutants, the otoliths were positioned close together at 33 hpf, were side by side at 48 hpf, had started to fuse at 66 hpf and had fully fused by 4 dpf (Fig 5B and 5C). This phenotype appeared to be fully penetrant (38/143 embryos from a cross between heterozygous parents; 26.6%). Semicircular canal pillars and the dorsolateral septum were present in the ears of mutant embryos, although formation of the ventral pillar was delayed. Overall, the ear shape appeared more symmetrical than in wild-type siblings (Fig 5B and 5C). We imaged ears from three mutant embryos at 3 dpf to analyse sensory patch formation (Fig 5D–5E’; S9 Fig). In all three ears imaged, the two maculae appeared fused or closely juxtaposed. Although the anterior and posterior elements of the fused macula were not obviously distinct, the overall shape retained some anteroposterior asymmetry. Hair cells of the anterior (utricular) macula were displaced medially, and in one of three ears imaged, were reduced in number. In the two other examples, however, normal numbers of hair cells were present (S9 Fig). The posterior macula was misshapen, and lacked the anterior extension present in the wild type. All three cristae were present (*n* = 3 ears; Fig 5D’ and 5E’; S9 Fig).

To understand the basis of the *hmx3a*^*SU3/SU3*^ mutant otic phenotype at 3–4 dpf, we examined expression of markers at earlier (otic vesicle) stages. At 24 hpf, expression of both *hmx3a* and *hmx2* was reduced in intensity within the otic epithelium. On the medial side of the ear, the spatial extent of *hmx3a* and *hmx2* expression was unchanged (Fig 5F–5I, black arrowheads), but levels were reduced (white arrowheads); anteroventrally, there was a reduction in *hmx3a* expression in presumed neuroblasts (Fig 5F’ and 5G’, blue and light blue arrowheads), and a mild posterior expansion of the spatial extent of expression for both genes in ventral otic epithelium (Fig 5G’ and 5I’, red arrowheads). Expression of the anterior markers *pax5* and *fgf3* was drastically reduced within anterior otic epithelium in *hmx3a*^*SU3/SU3*^ mutants at 24 hpf (Fig 5J–5M’, arrowheads), although expression of *fgf3* in pharyngeal pouch endoderm ventral to the ear was unaffected (Fig 5M and 5M’, double-headed white arrows). Expression of the same markers in *hmx3a*^*SU3/SU3*^ mutants at 27 hpf was similar, but otic expression of *hmx2* was more strongly reduced than that of *hmx3a*, especially in the anterior pole in the area corresponding to the normal expression domain of *fgf3* and *pax5* (S10 Fig). Expression of the posterior marker *fsta* at 30 hpf did not reveal any significant duplication of expression in anterior otic epithelium in *hmx3a*^*SU3/SU3*^ mutant ears (S10 Fig). Taken together, the results suggest that a loss of *hmx3a* function results in a similar phenotype to that of *fgf3*^*-/-*^ (*lia*^*t21142/t21142*^) mutants [3, 6, 28]. Although some anteroposterior asymmetry has been lost, the phenotype is not as strong as the double-posterior duplications that result from inhibition of all Fgf signalling or over-activity of Hh signalling, which show a complete loss of the anterior macula and lateral crista, and duplication of elements of the posterior macula [6, 12]. We conclude that *hmx3a* function is required for normal anterior otic expression of *pax5* and *fgf3*. However, loss of *hmx3a* function is not sufficient to result in a complete loss of anterior character and duplication of posterior structures at the anterior of the ear.

### Mis-expression of *hmx3a* is not sufficient to result in a duplication of anterior otic identity

**Fig 6:**
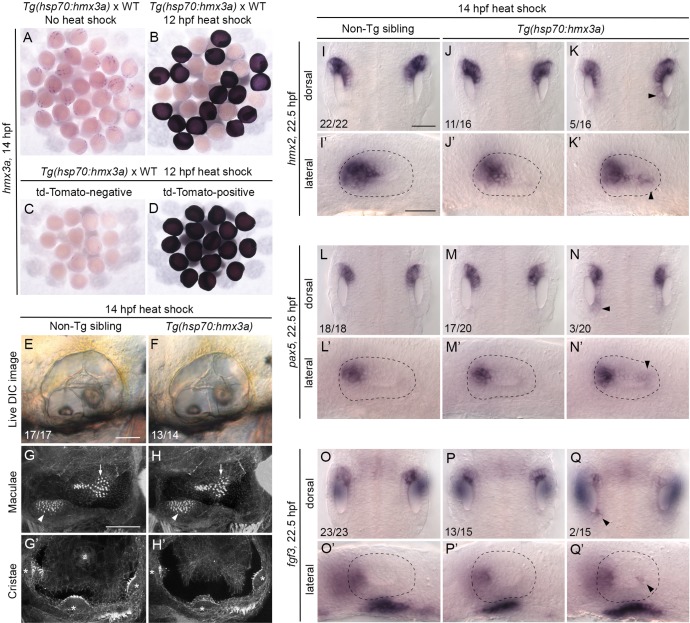
Mis-expression of *hmx3a* is not sufficient to generate an anterior ear duplication. (A–D) Control experiments to check for successful expression of the *hmx3a* transgene after heat shock at 12 hpf. Embryos were fixed and stained by in situ hybridisation two hours later, at 14 hpf. (A,B) A mixed batch of embryos from a cross between a fish hemizygous for the transgene and a wild type (WT). All embryos (31/31) showed the normal pattern of expression of *hmx3a* in the absence of heat shock (A). After a 60-minute heat shock at 12 hpf, ~50% of the batch (17/30) showed strong, systemic expression of the transgene at 14 hpf, as expected (B). All embryos shown in B were stained in the same tube. (C,D) Embryos heat-shocked for 60 minutes at 12 hpf were sorted on the basis of tdTomato expression before fixing. All tdTomato-negative embryos (14/14) were also negative for expression of the *hmx3a* transgene (C); all tdTomato-positive embryos (16/16) were also positive for *hmx3a* transgene expression (D). (E,F) Live DIC images of ears of non-transgenic (E) and transgenic (F) sibling embryos at 3 dpf (72 hpf), after a 30-minute heat shock at 14 hpf. (G–H’) Confocal images of FITC-phalloidin-stained ears at 3 dpf (72 hpf). Position and size of the two maculae (G,H, arrowheads and arrows) and three cristae (G’,H’, asterisks) were normal in both non-transgenic and transgenic sibling embryos after heat shock. (I–Q’) In situ hybridisation for otic marker genes in non-transgenic and *Tg(hsp70*:*hmx3a)* sibling embryos after a 30-minute heat shock at 14 hpf. Dorsal views of both otic vesicles (I–Q) and lateral views of a single otic vesicle (I’–Q’) are shown. Note weak ectopic expression of *hmx2* and *pax5*, and posterior otic expression of *fgf3*, in the otic vesicles of a minority of transgenic embryos (right hand column; arrowheads). Numbers of embryos showing the phenotypes are shown for each panel. WT, wild type (AB strain). Scale bar in E, 50 μm (applies to E–F); scale bar in G, 50 μm (applies to G–H’); scale bar in I, 50 μm (applies to I–Q); scale bar in I’, 50 μm (applies to I’–Q’).

As otic expression of the anterior markers *pax5* and *fgf3* is strongly reduced in *hmx3a*^*SU3/SU3*^ single mutants, and because expression of *hmx3a* is an early transcriptional response to manipulations of both Fgf and Hh signalling, we hypothesised that mis-expression of *hmx3a* alone would be sufficient to drive the expression of *pax5* and *fgf3* in the posterior of the otic placode and to give rise to a double-anterior duplication, bypassing the requirement for Fgf or Hh pathway manipulation. To test this idea, we created a transgenic line driving expression of the *hmx3a* coding sequence under the control of the *hsp70* heat-shock promoter. A 60-minute heat shock of *Tg(hsp70*:*hmx3a)* embryos at 12 hpf resulted in a robust and widespread expression of the *hmx3a* transgene two hours later (Fig 6A–6D). To avoid any disruption of otic placode induction, and to be comparable to the *fgf3* heat-shock experiments, we heat-shocked *Tg(hsp70*:*hmx3a)* embryos at 14 hpf to induce systemic mis-expression of *hmx3a*. After 30 minutes at 39°C, heat-shocked embryos were incubated at 33°C for 30 minutes before being returned to 28.5°C and incubated until 22.5 hpf, when they were fixed for processing and analysis, or until 3 dpf for assessment of any ear duplication (Fig 6E–6Q’).

Despite robust expression of the *hmx3a* transgene, the ears of *Tg(hsp70*:*hmx3a)* embryos heat-shocked for 30 minutes at 14 hpf did not recapitulate the duplicated double-anterior otic phenotype seen in *Tg(hsp70*:*fgf3)* embryos. Position and number of the otoliths, morphology of the semicircular canal pillars and position of the sensory patches in heat-shocked embryos were normal at 3 dpf (Fig 6E–6H’; compare with Fig 1B). At 22.5 hpf, otic vesicles were slightly smaller and rounder in heat-shocked transgenic embryos than those in heat-shocked non-transgenic siblings, but markers were expressed normally in most cases (Fig 6I–6Q’). Otic expression of *hmx2* was mildly up-regulated in a graded fashion (higher at the anterior) in 5/16 transgenic embryos at 22.5 hpf (Fig 6I–6K’), similar to the de-repression of *hmx* expression seen after Hh inhibition in wild-type embryos. There was also a mild up-regulation of *pax5* expression in posterior otic epithelium at 22.5 hpf in 3/20 embryos (Fig 6L–6N’), but *pax5* was never expressed in a broad zone as in the heat-shocked *Tg(hsp70*:*fgf3)* embryos. A weak patch of *fgf3* expression appeared in posterior otic epithelium at 22.5 hpf, similar to the duplicated zone of endogenous *fgf3* expression in heat-shocked *Tg(hsp70*:*fgf3)* embryos, but in only 2/15 embryos (Fig 6O–6Q’).

To check that the *hmx3a* transgene was functional, we sequenced cDNA from transgenic embryos, amplified with transgene-specific primers, which indicated that the open reading frame was intact (S11 Fig). We also examined the phenotype of transgenic embryos after an even earlier heat shock, during otic placode induction (8–9 hpf and 10–11 hpf). Here, we saw a range of otic abnormalities in 80% of transgenic embryos (*n* = 106), including missing otoliths, but some embryos also had small heads and eyes (S12 Fig). Ear patterning appeared normal in about 20% of transgenic embryos heat-shocked at these earlier stages. Longer (1- or 2-hour) heat shocks at 14–15 hpf also resulted in normal otic patterning (*n* = 49; S12 Fig). We conclude that the *hmx3a* transgene is likely to be functional, but that its mis-expression alone during otic placode stages (14–15 hpf, which should result in strong systemic expression until at least 17 hpf) cannot substitute for Fgf mis-expression or Hh inhibition in the generation of a double-anterior duplicated ear. Up-regulation of *hmx3a* in the ear at later stages, beyond 18 hpf, was not sufficient either, as our late *fgf3* heat shock experiments demonstrated (S2 Fig).

### A dynamical model of anteroposterior patterning in the zebrafish ear

**Fig 7:**
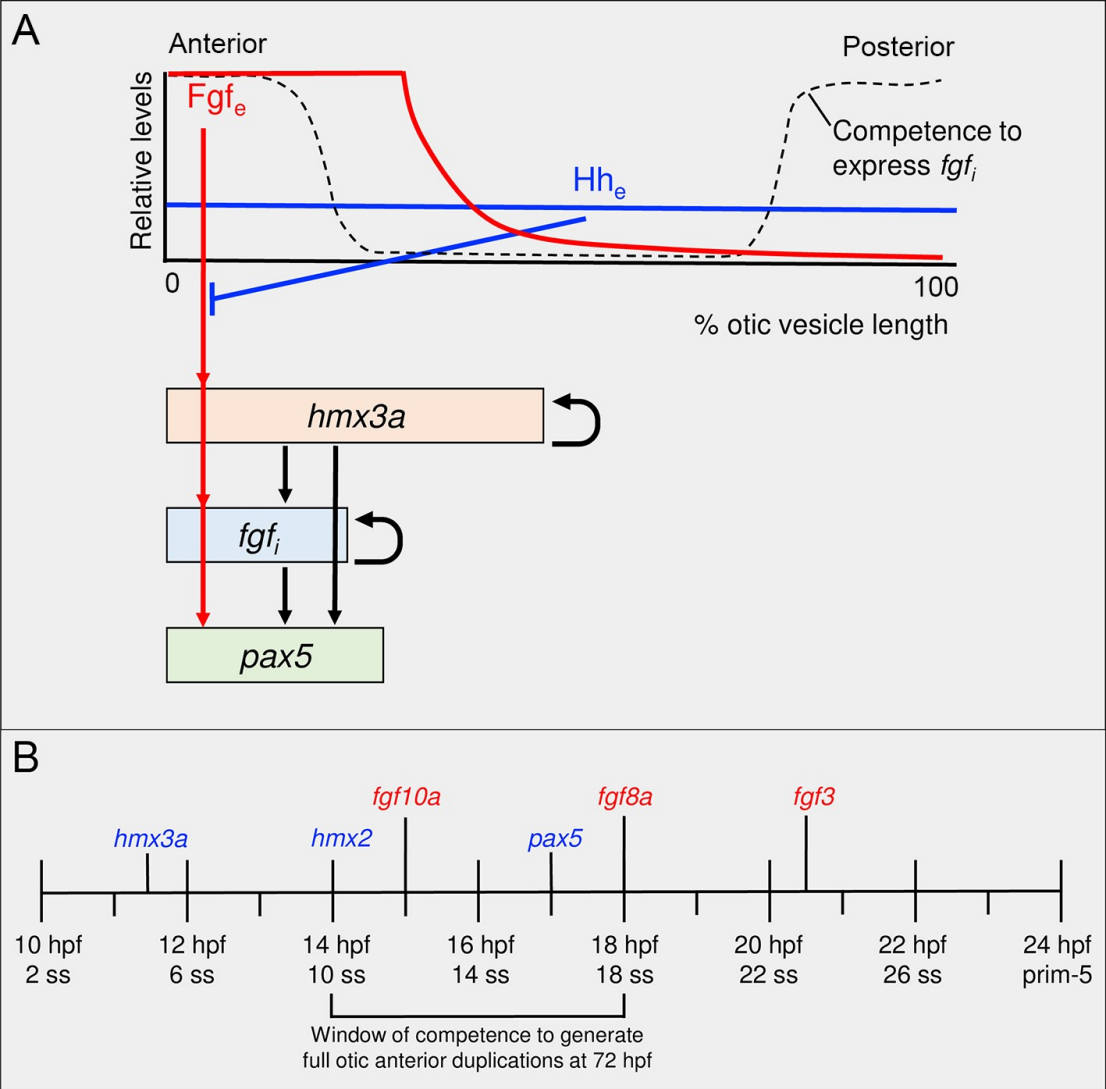
Proposed gene network and timeline for the acquisition of anterior identity in the zebrafish otic vesicle. (A) Proposed gene regulatory network. (B) Schematic timeline showing sequential onset of expression of anterior markers in the zebrafish otic placode and vesicle. Diagrams in A and B are based on the results of this study, together with previously published data [2–6, 9, 11, 12, 28]. Abbreviations: Fgf_e_, extrinsic Fgf protein; *fgf*_*i*_, intrinsic (otic vesicle) *fgf* gene expression; Hh_e_, extrinsic Hedgehog protein; hpf, hours post fertilisation; ss, somite stage.

Taken together, our data and those from previously-published studies suggest a temporal hierarchy of events for otic anteroposterior patterning dependent on extrinsic sources of Fgf and Hh signalling (Fig 7). To assess whether this network of inferred genetic regulatory interactions can account for the dynamic expression patterns we observe, we developed a mathematical model of otic anteroposterior patterning in the wild-type ear and following manipulation of the Fgf and Hh signalling pathways. The model is based on a set of differential equations describing the genetic interactions in the otic epithelium outlined in Fig 7A. In addition, patterning in the model is dependent on the existence of two sources of spatial information. First, we assume that otic competence to express *fgf* genes in response to Fgf and Hmx3a protein is localised to the two poles of the developing otic vesicle. This is necessary in the model to ensure that induced endogenous *fgf* mRNA expression in the otic epithelium (intrinsic *fgf* (*fgf*_*i*_)) is restricted to the poles, even when *fgf3* is expressed uniformly throughout the tissue following heat shock. Second, we represent the effect of *fgf* mRNA expression in rhombomere 4 as an anterior-to-posterior gradient of extrinsic Fgf (Fgf_e_) protein, present at high levels up to 30% of the otic vesicle length (corresponding to the position of the rhombomere 4/5 boundary), and forming a decreasing spatial gradient across the remainder of the otic axis (Fig 7A; S1 Model). Although we do not have a measure of actual Fgf protein concentration, our assumption is supported by measurements of fluorescence across the otic anteroposterior axis in the *Tg(dusp6*:*d2EGFP)* reporter line, which expresses a destabilised GFP variant as an indirect readout of Fgf activity [29] (S13 Fig).

We assume a spatially uniform level of Hh signalling throughout the otic epithelium (see discussion in [11]), and that Hh signalling antagonises the effects of Fgf signalling on otic anterior marker genes (*fgf*_*i*_, *hmx3a* and *pax5*) by increasing their response threshold for Fgf-induced expression. This functional attenuation is unlikely to be at the level of an immediate target of Fgf signalling such as *etv4*, as Hh inhibition did not result in major changes to *etv4* expression ([6]; this work). One possibility is that it could reflect integration of activity of the two signalling pathways at the level of binding sites in the promoters of the otic anterior genes. In addition, we propose that Hmx3a, together with Fgf and Hh, regulates its own expression and that of other genes in the network. Currently, our data do not distinguish whether these regulatory relationships are direct or indirect.

**Fig 8:**
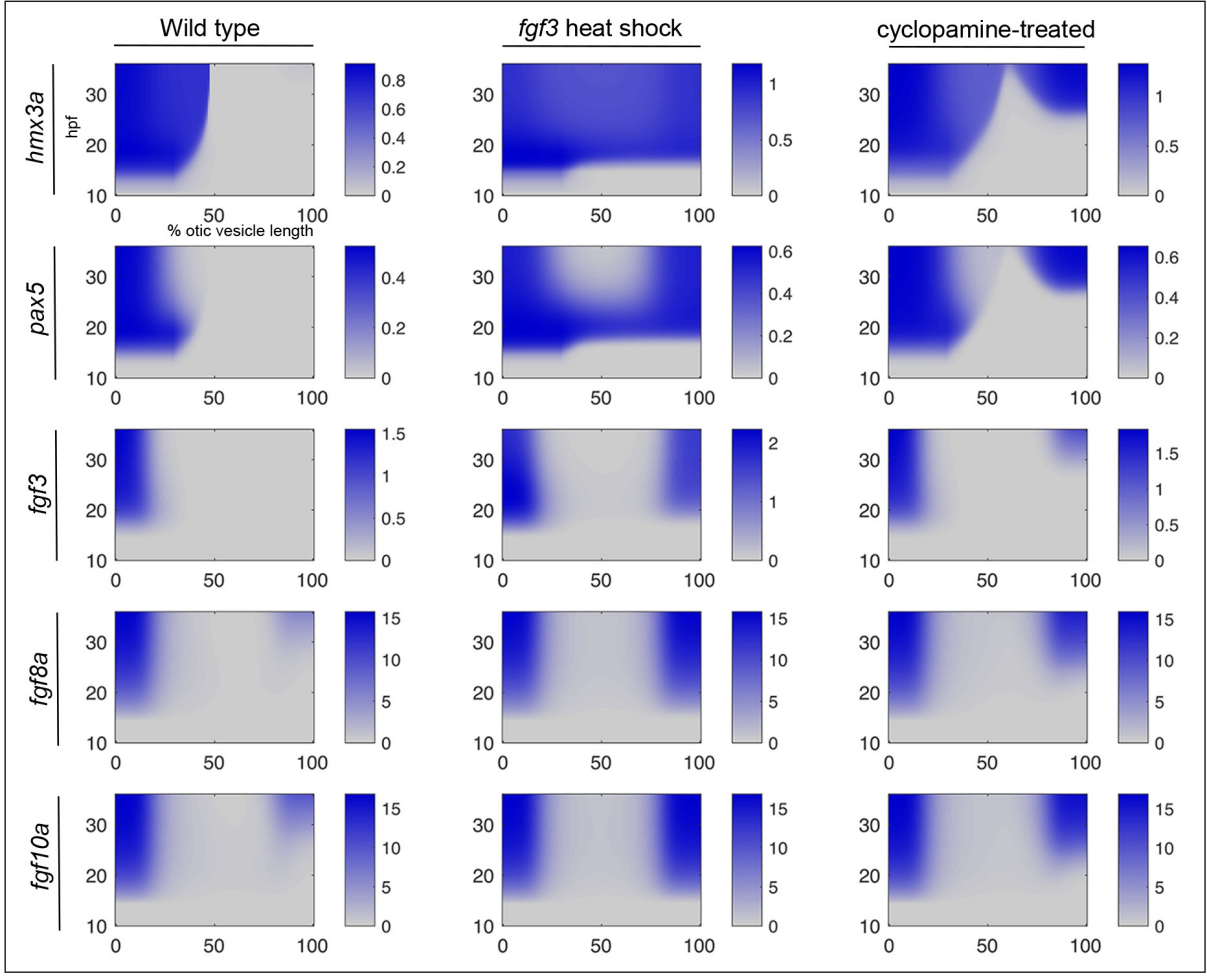
A model for the acquisition of anterior identity in the zebrafish otic vesicle. Solutions of a differential equation-based model to describe gene expression dynamics of the proposed network. Endogenous mRNA expression levels (blue) are shown as a function of position along the otic anteroposterior axis (*x* axis; % otic vesicle length from the anterior end) and time (*y* axis; hpf). Levels are shown in arbitrary units in the bars to the right of each panel. Exogenous *fgf3* mRNA from the transgene after heat shock is not shown. Left-hand column: wild type; middle column: with transient heat-shock induction of *fgf3* at 14 hpf; right-hand column: with inhibition of Hh signalling (cyclopamine treatment from 14–22.5 hpf). For full details, see S1 Model.

The dynamic behaviour of the model is presented in Fig 8 (for full details, see S1 Model). In wild-type embryos (Fig 8, left-hand column), expression of *hmx3a* and *pax5* is triggered in anterior otic tissue. The extent of expression is determined by the spatial reach of the extrinsic Fgf protein (Fgf_e_) gradient from rhombomere 4. Although all cells in the model are competent to express the anterior markers *hmx3a* and *pax5*, they do not receive sufficient Fgf_e_ to do so at the posterior otic pole in a wild-type embryo. After transient heat shock-induced systemic mis-expression of *fgf3* at 14 hpf (Fig 8, middle column), expression of both *hmx3a* and *pax5* is induced across the entire anteroposterior axis. However, the ability of heat shock-induced *fgf3* mis-expression to trigger endogenous intrinsic *fgf* (*fgf*_*i*_) expression requires the coincidence of both Fgf protein and competence to express *fgf*_*i*_ at the poles, and so *fgf*_*i*_ is not induced in the middle of the otic axis. After decay of heat shock-induced Fgf protein, expression of *pax5* is lost from central regions, but is maintained at the poles by Fgf signalling from *fgf*_*i*_ expression. By contrast, expression of *hmx3a* is maintained in central regions due to its autoregulation. De-repression of anterior markers after Hh pathway inhibition (Fig 8, right-hand column) results from a lowering of the threshold for response to Fgf signalling, establishing duplicate expression domains of *pax5* and *fgf3* at the posterior pole. Thus, although both heat shock-driven mis-expression of *fgf3* and inhibition of Hh signalling result in anterior duplications (Fig 8; compare the patterns at the 36 hpf time point), the transient dynamics exhibited by the model at earlier time points are distinct.

## Discussion

### Different transcriptional dynamics in the otic vesicle in response to manipulations of Fgf and Hh signalling

The zebrafish otic placode is a convenient system in which to understand the gene network dynamics that lead to asymmetries along the axis of a developing organ. Asymmetries in gene expression are evident from early (otic placode) stages, but the system is clearly equipotential, since either a gain of Fgf signalling or a loss of Hh pathway activity at otic placode stages can produce remarkably similar double-anterior zebrafish ears at 3 dpf [6, 11]. Interestingly, this final duplicated pattern arises via very different intermediate states in terms of gene expression patterns, depending on the signalling pathway that has been disrupted, as we have shown here. Mis-expression of *fgf3* at 14 hpf leads to a rapid loss of asymmetry, with broad expansion of anterior otic markers across the entire anteroposterior axis of the ear within a few hours of heat shock-driven mis-expression. Expression of *pax5*, which is required for normal development of the anterior (utricular) macula [3], later resolves into two discrete domains. By contrast, initial asymmetries in gene expression persist for several hours after inhibition of Hh pathway activity, with new duplicate expression domains of anterior markers (*pax5*, *fgf3* and *fgf8a*) only appearing nearly a day later at the posterior otic pole. We have identified *hmx3a* as an early otic transcriptional response to manipulations of both signalling pathways. However, although a loss of *hmx3a* demonstrates its requirement for normal otic patterning, this does not result in a complete double-posterior duplication, and mis-expression of *hmx3a* does not appear to be sufficient to drive the formation of a double-anterior ear.

Our data and mathematical model suggest that the Fgf/Hh system is sufficient to pattern the anteroposterior axis of the ear. In our scheme, there is only one input (extrinsic Fgf activity) that has a graded distribution across the otic anteroposterior axis. Notably, there is no need to infer an opposing graded input of extrinsic signalling activity that is high at the posterior of the ear. Although Retinoic Acid (RA) is thought to form such a gradient, and contributes to anteroposterior patterning in both the chick and zebrafish ear [30, 31], its activity can clearly be over-ridden by manipulations of Fgf or Hh signalling in generating either double-anterior or double-posterior zebrafish ears. Our model therefore differs from other models of axial patterning, for example in generation of dorsoventral pattern in the vertebrate neural tube. Here, information from two anti-parallel noisy gradients is integrated and refined by cross-repressing interactions between target genes, providing precise positional information along the axis [32, 33]. However, the sufficiency of our network and model does not necessarily rule out a contribution from the RA gradient in generating correct anteroposterior patterning in the wild-type ear.

At present, we do not have a full mechanistic explanation for the differences in response dynamics after manipulations of the Fgf and Hh signalling pathways. Although *hmx3a* responds rapidly to manipulation of Fgf signalling, its regulation may well be indirect; a recent study identified only one gene, *Etv5*, as a direct up-regulated target of Fgf signalling during induction of otic-epibranchial precursor cells in the chick [34]. In zebrafish, transcription of *etv4* and *spry4* is known to be an early response to Fgf signalling [35–37], with *spry4* expression appearing within one hour of implantation of a bead coated with Fgf8 protein during epiboly stages [37]. Our work here shows that robust, systemic expression of *etv4* occurs within two hours of the onset of heat shock in *Tg(hsp70*:*fgf3)* embryos; we had previously shown strong expression of *etv4* in the otic placode four hours after heat shock [6]. Thus, Etv4 is a good candidate for an immediate early transcriptional effector of Fgf signalling in our proposed genetic network. However, as *etv4* mRNA expression is not strongly perturbed by Hh pathway inhibition ([6]; this work), effects of Hh and Fgf on *hmx3a* expression are likely to be integrated further downstream, for example at the level of the *hmx3a* promoter. The slower response of *hmx3a* transcription to Hh inhibition might reflect the persistence of Hh pathway effectors, such as Gli activator proteins, which must be degraded before the effect of inhibiting Smoothened with cyclopamine can take effect.

As the otic vesicle develops, additional levels of regulation are likely to contribute to the regulation of *hmx3a* and other genes in the network. For example, in the chick, regulation of *Hmx3* expression in the dorsolateral otocyst has recently been shown to be influenced by both Shh and non-canonical BMP signalling through PKA and GLI3R [38]. In addition, negative feedback on otic *fgf* expression via *sprouty* genes [4] is likely to help to restrict gene expression to the poles and sharpen expression domain boundaries within the otic epithelium.

### Requirement for intrinsic factors at the otic anterior and posterior poles in establishing a duplicate pattern after signalling pathway manipulation

One of the intriguing features of the double-anterior ears is that systemic mis-expression of an anteriorising factor (*fgf3*) gives rise to two defined and separate anterior maculae with mirror-image symmetry, rather than establishing uniform anterior identity across the entire medial otic domain. The final duplicate pattern develops despite the initial broad expression of anterior markers after heat shock, which demonstrates that the entire medial side of the otic placode and vesicle is competent to express *hmx* genes and *pax5* in response to Fgf signalling. However, a day after heat shock, expression of *pax5* is lost from the centre of this domain and only maintained at the anterior and posterior poles of the otic vesicle, suggesting either that expression is subsequently repressed in the central domain, or that an intrinsic factor or factors is required to maintain expression at the poles. Attractive candidates for the latter role include *atoh1a*, which is expressed in discrete domains at the anterior and posterior otic poles at 14 hpf [9]. Atoh1a is thought to act in a positive feedback loop together with Fgf signalling in the zebrafish ear [9, 39]. Fgf pathway activity is also observed at both poles of the otic vesicle at 24 hpf (this work), 28 hpf and 50 hpf using a destabilised fluorescent transgenic reporter, *Tg(dusp6*:*d2EGFP)* [29]. We hypothesise that a positive feedback loop involving a pole-specific factor and all three *fgf* genes contributes to the maintenance of anterior-specific gene expression and generation of the double-anterior pattern. This builds on previous feedback models for anterior otic patterning and the regulation of otic *pax5* expression [2, 3].

A similar broad medial expansion of *hmx3a* and *pax5* has been recently reported to result from systemic mis-expression of *sox2* or *sox3* at 12.5 hpf [40]. However, this early mis-expression results in a smaller and mis-shapen otic vesicle (most likely due to a disruption of otic induction), and phenotypes were not followed beyond 30 hpf. It will be interesting to see whether a duplicated anterior pattern results from these manipulations.

### Comparison of the *hmx3a*^*SU3/SU3*^ and *fgf3*^*t21142/t21142*^ mutant otic phenotypes in the zebrafish

The otic phenotype of *hmx3a*^*SU3/SU3*^ single mutants closely resembles that of *hmx3a/hmx2* double morphants [2], and of *fgf3*^*t21142/t21142*^ mutants [3, 6, 28]. The similarity of the *fgf3* and *hmx3a* otic mutant phenotypes suggests that a major role for the extrinsic Fgf3 signal is to activate *hmx3a* expression in anterior otic epithelium. Note that pharmacological inhibition of all Fgf signalling [6] or over-activity of the Hh pathway [12] both result in a stronger otic phenotype than in *hmx3a*^*SU3/SU3*^ mutants. The retention of some anteroposterior asymmetries in gene expression and the fused sensory macula in *hmx3a*^*SU3/SU3*^ mutants, together with the presence of the lateral crista, suggest that the *hmx3a*^*SU3/SU3*^ otic phenotype, like that of *fgf3*^*t21142/t21142*^ mutants, does not represent a complete double-posterior duplication. We also failed to see strong ectopic expression of the posterior marker *fsta* at the anterior of the ear in *hmx3a*^*SU3/SU3*^ mutants, although this is a less reliable indicator of posterior duplication; it is expressed at both poles of the ear following strong Fgf inhibition [6], but lost altogether in the extreme double-posterior ears that can result from elevated Hh signalling [12].

Despite the similarities between the loss-of-function phenotypes for *fgf3* and *hmx3a* in the zebrafish ear, the gain-of-function effects for each of the two genes are strikingly different. Whereas mis-expression of *fgf3* at 14 hpf reliably generates a complete double-anterior ear, mis-expression of *hmx3a* at the same time point had very little effect on otic development. It is remarkable just how robust the embryo is to this kind of perturbation, considering that the systemic high levels of transgene expression must be energetically expensive to support. Indeed, there is usually some transient developmental delay after heat shock, but gross patterning of the ear at 3 dpf appeared normal in *Tg(hsp70*:*hmx3a)* heat-shocked embryos.

Why, then, is *hmx3a* ineffective in establishing duplicate anterior development when mis-expressed? It is possible that it needs to be delivered together with *hmx2*; the two genes are tightly linked on zebrafish chromosome 17 [41], spatially co-expressed in the zebrafish otic vesicle (although with different temporal onset) [2], and are known to have partially overlapping roles in the mouse ear [42]. A predicted *hmx3b* gene (RefSeq XM_017358610.2) is also present in the zebrafish genome on chromosome 12. If a second Hmx family protein or other binding partner was limiting, this might explain the lack of activity of the mis-expressed *hmx3a* transcript. Alternatively, Hmx3a could act as a competence factor, only functioning in the context of high Fgf or low Hh signalling to initiate duplicate anterior otic development. It is also possible that Fgf signalling abrogates an unidentified negative regulator of the otic anterior gene network at the same time as activating the expression of *hmx3a*. In the presence of such an inhibitor, systemic over-expression of *hmx3a* would be ineffective at activating the expression of genes such as *hmx2*, *pax5* and *fgf3* in posterior otic domains.

### Comparison of the effects of loss of *Hmx3* function on otic development between zebrafish and amniotes

Anterior-specific otic expression of *Hmx3* and *Hmx2*, including their temporal order of expression onset in the ear, is conserved between zebrafish, mouse and chick [2, 43–45]. Loss of *Hmx3* function in the mouse causes a range of reported otic defects with variable penetrance and expressivity, which depend on the nature of the targeted mutant allele. A homozygous targeted deletion of exon 1 and part of exon 2 of *Hmx3* resulted in a variable disruption of the lateral (horizontal) and posterior semicircular canal ducts, and loss of the lateral (horizontal) crista [46]. A weaker phenotype was seen after disruption of the homeodomain in exon 3 of *Hmx3*; in these mutants, all three semicircular canal ducts were present, but the lateral (horizontal) ampulla and crista were missing. The utricular and saccular maculae were juxtaposed in a common utriculosaccular chamber [42, 44], as we found in the zebrafish *hmx3a*^*SU3/SU3*^ mutant. A notable difference between the mouse and zebrafish mutants is the presence of all three cristae, including the lateral crista, in the zebrafish *hmx3a*^*SU3/SU3*^ mutants. Formation of the ventral pillar for the lateral canal was also present, although delayed. It will be interesting to see whether mutations in *hmx2* (not currently available) affect morphogenesis of the zebrafish semicircular canal system; in the mouse, targeted disruption of *Hmx2* results in a loss of all three semicircular canal ducts, with partial or complete loss of some ampullae and cristae, in addition to a fused utriculosaccular chamber [47]. In humans, *HMX3* and *HMX2* are located together, close to *FGFR4*, on chromosome 10; hemizygous microdeletions that remove all three genes are thought to be causative for syndromes characterised by inner ear morphological anomalies, vestibular dysfunction and sensorineural hearing loss [48, 49].

In conclusion, our study demonstrates that although Fgf gain-of-signalling and Hh loss-of-signalling produce similar morphological duplications of the zebrafish ear, they do so via distinct dynamical patterns of gene expression, providing valuable insights into normal anterior otic development. In addition, we determine that *hmx3a*, a gene expressed as an early transcriptional response to both Fgf and Hh manipulation, has a conserved role in correct separation of the sensory maculae within the otic vesicle, and is required—but not sufficient—for normal anterior otic development. We have also shown that our proposed genetic network for zebrafish otic anterior development can be recapitulated with a mathematical model that assumes interactions between a graded extrinsic source of Fgf, a uniform inhibitory influence of Hh, and equipotential competence to adopt an anterior identity at the otic poles. Interactions between these inputs and their downstream targets within the otic tissue (*hmx3a*, *hmx2*, *pax5* and *fgf* genes) lead to correct anteroposterior patterning in the developing zebrafish ear. The model will be a useful framework for further elucidation and functional validation of the proposed gene regulatory network required for the acquisition of anterior otic identity in the zebrafish.

## Materials and methods

### Ethics statement

All animal work in the Whitfield lab was covered by licencing from the UK Home Office (PPL 40/3655, P66302E4E). All zebrafish experiments conducted in the Lewis lab were approved by the Syracuse University Institutional Animal Care and Use Committee (IACUC).

### Animals

Adult zebrafish (*Danio rerio*) were kept in circulating water at 28.5°C with a 14-hour light/10-hour dark cycle. The wild-type line used was AB; mutant alleles were *hmx3a*^*SU3*^ (this work; see below for details), mibta52b [23–25], and *smo*^*hi1640Tg*^ [19]; transgenic lines were *Tg(dusp6*:*d2EGFP)* [29], *Tg(hsp70*:*fgf3)* [13], *Tg(hsp70*:*fgf8a)*^*×17*^ [14] and *Tg(hsp70*:*hmx3a)* (this work; see below for details). The *Tg(hsp70*:*fgf3)* line was maintained on a *mitfa*^*w2/w2*^ background to reduce pigmentation. Embryos were staged as described [15] and incubated at 28.5°C in E3 (5 mM NaCl, 0.17 mM KCl, 0.33 mM CaCl_2_, 0.33 mM MgSO_4_, 0.0001% methylene blue), unless otherwise indicated.

### Heat shock

Embryos were cultured in E3 at 28.5°C prior to heat shock. For heat shock, embryos from either a cross between two hemizygous transgenic carriers, or an outcross between a transgenic carrier and a wild-type, were transferred to 25 ml of preheated E3 in a Falcon tube and incubated at 39°C for 30 minutes, unless otherwise indicated. Embryos were then returned to their original plates of E3, which had been preheated to 33°C during the heat shock, and incubated for a further 30 minutes at 33°C. Plates were then returned to 28.5°C and incubated until embryos reached the desired stage for fixation. In heat-shock experiments with mixed batches of transgenic and non-transgenic embryos, a transgenic genotype was confirmed by expression of tdTomato in *Tg(hsp70*:*hmx3a)* embryos or abnormal shape of the yolk extension in *Tg(hsp70*:*fgf3)* embryos, in addition to analysis of the phenotypes described in the text.

### Cyclopamine treatment

Embryos were treated in 12-well plates (3 ml total volume; ≤30 embryos per well) at 28.5°C with InSolution Cyclopamine, *V*. *californicum* (Calbiochem). Chorions were punctured with a sterile hypodermic needle prior to treatment to improve compound penetration. After treatment, embryos were washed twice in E3 before either being fixed or incubated in E3 before fixation later. Vehicle-only controls consisted of an equivalent volume of the solvent (ethanol) to that used in the highest experimental treatment concentration. Embryos from the same batch (siblings) were randomly allocated into control and treatment groups.

### In situ hybridisation

Embryos were dechorionated and fixed in 4% paraformaldehyde overnight at 4°C. In situ hybridisation was carried out as described [50]. For most experiments, at least 25 embryos (biological replicates) were stained in any given batch. Where relevant, numbers of embryos with the phenotype of interest and total number in the batch (e.g. 29/30) are shown directly on the figure panels (see figure legends for details). Analysis of gene expression via in situ hybridisation is not quantitative, but we have chosen markers that give a clear and robust qualitative response to changes in signalling pathway activity. We have used information from these spatial expression patterns to infer parameters for the mathematical model (see below). Where appropriate, we have measured the spatial extent of expression along the medial side of the otic vesicle in a dorsal view using ImageJ.

### Generation of a template for the *fgf3* 3’ UTR-specific in situ hybridisation probe

The 3’ UTR of *fgf3* was amplified from wild-type (AB strain) genomic DNA in a nested PCR, incorporating the T7 promoter, using the following primers: F1 TCTCTTGACACAGATGGAGATCC, R1 AATATACAAAGTACTCCTGATTGCA; F2 AAGGCCACTGAGAGTCCAAAA, T7-R2 TAATACGACTCACTATAGGGCAGTAGCCTATCACATGTACGT. Each PCR was run for 30 cycles with an annealing temperature of 53°C.

### Generation of the *hmx3a*^*SU3*^ mutant allele

The single guide RNA (sgRNA) targeting *hmx3a* was designed using CHOPCHOP [51, 52]. The sgRNA DNA template was generated using the cloning-free method of Gagnon and colleagues [53]. The template was transcribed and purified using the standard protocols of the MEGAshortscript T7 kit (AM1354, Thermo Fisher Scientific). sgRNA was resuspended in 40 μl of sterile water and the concentration and purity measured using spectrophotometry, before aliquoting for storage at -80°C. To make *Cas9* mRNA, *pCS2-nls-zCas9-nls* plasmid DNA [54] was digested with *NotI* and purified by phenol:chloroform extraction, before being transcribed and purified using standard protocols of the mMESSAGE mMACHINE SP6 kit (AM1340, Thermo Fisher Scientific). The resultant mRNA was resuspended, assayed and stored as for the sgRNA. The single cell of one-cell stage AB wild-type embryos was injected with 2 nl of a mixture of 200 ng/μl sgRNA + 600 ng/μl *nls-ZCas9-nls* mRNA. Founders were identified by high resolution melt analysis, using the following primers: PMA F: CGAATGCTAATTTGGCCTCTATTACT and PMA R: TTTTGTTGTCGTCTTCATCGTCC, and Precision Melt Supermix for High Resolution Melt (HRM) Analysis (172–5112, Bio-Rad), performed on a CFX96 Touch System (1855195, Bio-Rad), equipped with Precision Melt Analysis Software (1845025, Bio-Rad). Amplification data were generated using the following program: 95.0°C for 3 minutes, followed by 45 cycles of 95.0°C for 15 seconds, 60.0°C for 20 seconds and 70.0°C for 20 seconds. Melt data were generated using the following program: 65.0°C for 30 seconds, 65.0°C–95.0°C at an incremental rate change of 0.2°C, held for 5 seconds each step, 95.0°C for 15 seconds. Stable F1 heterozygous fish were confirmed by sequencing. All subsequent genotyping was performed by PCR, using the primers F: TGGCAAAGTGACACGACCAG and R: GAGAACACCGTGCGAGTTTTC, Taq DNA Polymerase (M0320S, NEB) and the PCR program: (94.0°C for 2 minutes, 35 cycles of: 94.0°C for 30 seconds, 64.9°C for 30 seconds and 72.0°C for 30 seconds, followed by a final extension at 72.0°C for 2 minutes). The *hmx3a*^*SU3*^ allele is a 69 bp insertion, flanked on either side by 2-base mismatches. The insertion introduces a premature stop codon at nucleotides 352–354 of the edited coding sequence. The insertion in the mutant allele can be distinguished by performing gel electrophoresis on a 2% TBE agarose gel (100V for 40 minutes). The wild-type allele generates a 331 bp product, compared with the 400 bp mutant allele product.

### Generation of the *Tg(hsp70*:*hmx3a)* line

The zebrafish *hmx3a* cDNA sequence (RefSeq NM_131634.2), including the complete open reading frame, endogenous Kozak sequence and 15 bp of 3’ UTR, was cloned into a *Tol2*-containing *ubi*:*tdTomato* destination vector, flanked by a 5’ *hsp70* promoter and a 3’ SV40 late polyadenylation signal sequence, using the Tol2kit [55] (Invitrogen). 50 ng of this construct were injected into one-cell stage embryos together with 50 ng of in vitro-transcribed transposase RNA. Injected embryos (G0) were raised to adulthood, and their progeny (F1) screened for expression of the tdTomato marker. F1 embryos with positive expression were raised to adulthood to generate a stable *Tg(hsp70*:*hmx3a)* transgenic line. Progeny were tested by in situ hybridisation after heat shock to check for misexpression of the *hmx3a* transgene.

To check that the *hmx3a* coding sequence was intact, cDNA was generated from heat-shocked embryos from an in-cross of *Tg(hsp70*:*hmx3a)* fish by RT-PCR and sequenced. PCR primers (F: TACGACTCACTATAGGGCGAATTG; R: GCAATTAACCCTCACTAAAGGGAA) were designed to be transgene-specific, binding to residual multiple cloning site sequences within the integrated construct, with a predicted amplicon size of 1053 bp. Sequences were aligned and displayed using Ensembl MUSCLE and ExPasy BOXSHADE.

### Phalloidin staining

Embryos were fixed in 4% PFA overnight, washed in PBS (3×10 minutes) and permeabilised in 2% Triton-X100 (Sigma) for 3–4 days at 4°C. Following further washes in PBS (3×5 minutes), embryos were stained with FITC-phalloidin (1:20; Sigma) or Alexa Fluor 647-phalloidin (1:100; Thermo) in PBS (overnight, 4°C). Embryos were washed in PBS (3×60 minutes), dissected in PBS and mounted in Vectashield (Vectorlabs) prior to confocal imaging.

### Microscopy, photography and image processing

Live and fixed embryos were imaged on either an Olympus BX51 or a Zeiss Axio Imager M1 compound microscope using brightfield, DIC and epifluorescence optics as appropriate, and CellB or Axiovision image acquisition software, respectively. For confocal imaging, either a Nikon A1 or a Zeiss LSM 710 confocal microscope was used. For fluorescent imaging requiring large fields of view, a Zeiss Axio Zoom.V16 stereomicroscope with Zen acquisition software was used. The S1 Movie and associated still images shown in S13 Fig were acquired with a Zeiss Z.1 light-sheet microscope. Sample drift was corrected using the Manual Drift Correction plugin within FIJI (Fiji Is Just ImageJ) [56]. FIJI was used for all image processing. Figure panels were assembled using Adobe Photoshop 2015.5.0. All dorsal views (except in S13 Fig) are shown with anterior to the top; lateral views show anterior to the left.

### Statistical analysis

Statistical analyses were performed using GraphPad Prism version 7.0c for Mac OSX (GraphPad software, La Jolla California USA, www.graphpad.com). See figure legends and S1 Data for details.

### Mathematical model

Full information for generation of the mathematical model, including a list of parameters used, is given in S1 Model.

## Supporting information

S1 Fig Normal ear development in control embryos for experimental manipulations of Fgf and Hh signalling. (A–E) Differential interference contrast (DIC) images of ears in live embryos at 3 dpf (72 hpf). (F–J’) Confocal images of FITC-phalloidin stains, revealing stereociliary bundles on sensory hair cells in the maculae (F–J) or cristae (F’–J’), as shown in Fig 1. The first column (‘Wild type’) repeats column 1 of Fig 1 for comparison. Subsequent columns show representative images of controls for the experiments shown in Fig 1. All ears shown were patterned normally, although views and focal planes differ slightly. All ears were of normal size and had two normally-positioned otoliths (A–E), two maculae of normal size, shape and position (F–J), and three cristae (F’–J’). Lateral views; anterior to the left. Cyc, cyclopamine. Scale bar in A, 50 μm (applies to A–E); scale bar in F, 50 μm (applies to F–J’).
(TIF)

S2 Fig Effects of late mis-expression of *fgf* genes on otic patterning. (A–D) Differential interference contrast (DIC) images of ears in live embryos at 3 dpf (72 hpf); lateral views with anterior to the left. Control embryos are non-transgenic siblings subjected to the same heat-shock treatment at 18 hpf. Representative phenotypes are shown; numbers of embryos showing the phenotype are indicated on each panel. Note the relatively normal size and shape of the ears after heat shock in transgenic animals. The focal plane for all panels is at the level of the anterior otolith; note that the posterior otolith (out of focus) is positioned dorsomedially, relative to the anterior otolith, in both control and transgenic ears. (E–H’) In situ hybridisation for *hmx3a* (E–F’) and *pax5* (G–H’) at 22.5 hpf. Note the expansion of expression for both markers after heat shock of transgenic animals. E–H are dorsal views showing both ears; E’–H’ are lateral views with anterior to the left. Scale bar in A, 50 μm (applies to A–D); scale bar in E, 50 μm (applies to E–H’).
(TIF)

S3 Fig Time-course of expression of *etv4* mRNA after heat shock in *Tg(hsp70*:*fgf3)* embryos. Embryos were heat-shocked for 30 minutes at 39°C at 14 hpf (the 10-somite stage), fixed at various times after the onset of heat shock as shown (top), and processed for in situ hybridisation for *etv4*. All embryos were stained and photographed using bright field optics under identical conditions, and scored as having ‘light’ or ‘dark’ expression (presumed transgenic and non-transgenic embryos, respectively; 75% of the batch was expected to be transgenic). Number of embryos with the phenotype shown and total number in the batch are shown directly on the panels (e.g. 3/12). Expression levels at the 10-somite stage before heat shock (A,A’) were very variable, possibly due to leaky expression of the transgene, but corresponded to the published spatial pattern of expression [22]. Robust, systemic up-regulation of *etv4* was seen in embryos 2 hours after heat shock (E,E’). This persisted 8.5 hours after heat shock; here, 4/5 presumed transgenic embryos with abnormal morphology also had strong *etv4* expression (F’). Morphology was normal in presumed non-transgenic siblings showing the endogenous expression pattern of *etv4* (F). F,F’ show lateral views; all other panels are dorsal views of flat-mounted embryos.
(TIF)

S4 Fig Detailed time-course of expression of *hmx2* after early *fgf3* mis-expression. In situ hybridisation of otic expression of *hmx2* in *Tg(hsp70*:*fgf3)* embryos following a 30-minute heat shock (HS) at the 10-somite stage (14 hpf). Controls (A–D’) were sibling non-transgenic embryos subjected to the same heat shock. Numbers in the dorsal view panels indicate the number of embryos with the phenotype shown and total number (e.g. 5/14) from a mixed batch of transgenic and non-transgenic embryos in each pair of panels; 75% of the batch was expected to be transgenic. The first and last rows are biological replicates of data shown in Fig 2. Note the weakening of expression in central medial otic epithelium by 25.5 hpf in transgenic embryos. By 36 hpf, in a lateral view, *hmx2* is expressed throughout the ventral floor of the otic vesicle in transgenic embryos, associated with a thicker epithelium in posterolateral regions (D’, H’, brackets). All dorsal views show anterior to the top; all lateral views show anterior to the left. Scale bar in A, 50 μm (applies to all panels).
(TIF)

S5 Fig Detailed time-course of expression of *pax5* after early *fgf3* mis-expression. In situ hybridisation for otic expression of *pax5* in *Tg(hsp70*:*fgf3)* embryos following a 30-minute heat shock (HS) at the 10-somite stage (14 hpf). Controls (A–D’) were sibling non-transgenic embryos subjected to the same heat shock. Numbers in the dorsal view panels indicate the number of embryos with the phenotype shown and total number (e.g. 5/17) from a mixed batch of transgenic and non-transgenic embryos in each pair of panels; 75% of the batch was expected to be transgenic. The first and last rows are biological replicates of data shown in Fig 2. Note that the ectopic expression in transgenic embryos has already resolved into two domains by 25.5 hpf, and two discrete domains persist at 36 hpf. At 36 hpf, in a lateral view, the ectopic domain of *pax5* is associated with a thicker epithelium (D’, H’, brackets). One embryo at 36 hpf was unable to be scored. All dorsal views show anterior to the top; all lateral views show anterior to the left. Scale bar in A, 50 μm (applies to all panels).
(TIF)

S6 Fig Expression of target genes of the Hh and Fgf pathways after cyclopamine treatment. (A,B) In situ hybridisation for the Hh pathway target gene *ptch2* at 22.5 hpf, after treatment from 14.5 hpf with vehicle (ethanol) only (A) or 100 μM cyclopamine (cyc; B). The head and yolk of the embryo are shown. Lateral views; anterior to the left. Expression is reduced but not abolished in cyclopamine-treated embryos (B). (C–D’) In situ hybridisation for the Fgf target gene *etv4* at at 22.5 hpf, after treatment from 14.5 hpf with vehicle (ethanol) only (C,C’) or 100 μM cyclopamine (cyc; D,D’). There are no major changes to the otic expression of *etv4* at this stage after cyclopamine treatment. C,D show dorsal views of the two otic vesicles with anterior to the top; C’,D’ are lateral views of the otic vesicle with anterior to the left. Scale bar in A, 200 μm (applies to B); scale bar in C, 50 μm (applies to D); scale bar in C’, 50 μm (applies to D’).
(TIF)

S7 Fig Otic expression of *hmx* genes after genetic or pharmacological inhibition of Hh signalling. (A–D’) In situ hybridisation for *hmx* genes at 22.5 hpf in the otic vesicles of phenotypically wild-type and *smo*^*hi1640Tg/hi1640Tg*^ mutant embryos. In the mutant, otic expression extends posteriorly in a graded fashion at this stage. A–D show dorsal views of the two ears, with anterior to the top; A’–D’ show lateral views of the otic vesicle with anterior to the left. Scale bar in A, 50 μm (applies to A–D); scale bar in A’, 50 μm (applies to A’–D’). (E) Measurements of the extent of in situ hybridisation stain along the medial edge of the otic vesicle at 22.5 hpf, expressed as a percentage of the length of the medial edge of the otic epithelium. The expression domain of both *hmx* genes extends posteriorly in *smo*^*hi1640Tg/hi1640Tg*^ mutant embryos, and after treatment with 100 μM cyclopamine from 14 hpf. One-way ANOVA with Šídák’s post-test correction for multiple comparisons: ****p<0.0001; *p = 0.0119; ns, non-significant (*p* = 0.9998). Error bars represent the 95% confidence interval for the mean. *n* indicates number of embryos; two ears were measured for each embryo, with each ear measurement shown as a separate data point. See also S1 Data.
(TIF)

S8 Fig Retention of otic anteroposterior asymmetry in *mib1*^*ta52b/ta52b*^ mutants. (A–H’) In situ hybridisation for anterior otic markers in phenotypically wild-type sibling (A–H) and *mib1*^*ta52b/ta52b*^ mutant (A’–H’) ears at 25–26 hpf. All markers are expressed in the normal anterior domain in the *mib1*^*ta52b/ta52b*^ mutant otic vesicle, but at lower levels than in sibling embryos. An anterior gap in expression is seen in the *hmx3a* expression domain in the mutant (B’). Expression of *fgf3* in pharyngeal endoderm ventral to the ear is unaffected (F’). There is no evidence for expansion, strengthening or posterior duplicated otic expression for any of the genes tested. Numbers in the dorsal view panels indicate the number of embryos identified via morphological criteria as sibling or mutant that have the staining pattern shown. Scale bar in A, 50 μm (applies to all panels).
(TIF)

S9 Fig Maculae are fused, but cristae are normal, in *hmx3a*^*SU3/SU3*^ mutant ears. (A–F”) Confocal images of FITC-phalloidin-stained ears at 3 dpf (72 hpf); maximum intensity projections of selected *z*-stacks. Three ears were imaged for each genotype; note the fused maculae, but normal presence of three cristae, in each of the mutant ears (D–F”). The middle row of panels in each set is a duplicate of the panels above, with counts for visible hair cells in the anterior macula (blue) and posterior macula (red). The distinction between the anterior and posterior parts of the fused macula in panels D’–F’ was estimated based on hair cell position. Anterior macula counts in the wild-type samples are likely to be underestimates, as only some of the hair cells were visible in this orientation. It was not possible to distinguish any individual hair cells in the anterior macula in wild-type ear 2 (B’). The number of ears imaged was too small to draw firm conclusions about any changes in hair cell number in either macula. Note that the panels for wild-type ear 3 and *hmx3a*^*SU3/SU3*^ mutant ear 3 are reproduced in Fig 5. Scale bar, 50 μm (applies to all panels).
(TIF)

S10 Fig Expression of otic markers at 27–30 hpf in *hmx3a*^*SU3/SU3*^ mutants. (A–H’) Reduction in expression of anterior otic markers in the otic vesicle of *hmx3a*^*SU3/SU3*^ mutants at 27 hpf. Black arrowheads mark expression domains in wild-type ears and in presumptive neuroblasts anteroventral to the ear that are reduced or missing in mutants (white arrowheads). (I–J’) Expression of the posterior otic marker *follistatin-a* (*fsta*) in the otic vesicle at 30 hpf. Although weak *fsta* expression was detected in anterior otic epithelium in mutants (J’, black arrowhead), levels were not above the natural variation seen in wild-type siblings. Numbers in panels A–J indicate numbers of embryos genotyped as either wild type or homozygous mutant that showed the representative expression patterns illustrated. Scale bar in A, 50 μm (applies to A–J); scale bar in A’, 50 μm (applies to A’–J’).
(TIF)

S11 Fig Sequencing of transgene-derived cDNA from *Tg(hsp70*:*hmx3a)* embryos. Sequence of cDNA from three *Tg(hsp70*:*hmx3a)* embryos (Embryo 1–3) after heat shock, aligned to the NCBI RefSeq for zebrafish *hmx3a* (NM_131634.2). The start (ATG) and stop (TGA) codons of the open reading frame are shaded in pink. Exact matches with the RefSeq are shaded in black.
(PDF)

S12 Fig Development of embryos and otic phenotypes after mis-expression of *hmx3a* at different time points. (A–H) Transgenic *Tg(hsp70*:*hmx3a)* and non-transgenic sibling embryos were heat-shocked for 1 hour at the times indicated, and grown on to 3 dpf for examination of general morphology and any otic phenotype. Representative examples are shown. (I) Graphical representation of the results from 1- and 2-hour heat shocks. Abbreviations: N, normal positioning of both anterior (ventrolateral) and posterior (posteromedial) otoliths; S1—anterior otolith in wild-type ventrolateral position but posterior otolith more ventrally positioned; S2—single otolith in one ear with two wild-type positioned otoliths in the contralateral ear; S3—single otolith in both ears; *n*, number of embryos. See also S1 Data.
(TIF)

S13 Fig Expression of a fluorescent reporter for Fgf activity in the hindbrain and otic placode. (A–E”) Light-sheet imaging and fluorescence measurements of a representative *Tg(dusp6*:*d2EGFP)* embryo from the 8-somite stage to the 16-somite stage (A–D), and a second representative embryo at 24 hpf (E). A dorsal view of the hindbrain and right-hand otic region (dotted outline) is shown; anterior is to the left. Rhombomere 4 (r4) expression is bright during the 8–16-somite stages. Expression levels were averaged over a 28.7 μm-wide band, over 20 *z*-sections at intervals of 1 μm, both in the hindbrain posterior to the r3/r4 boundary (orange; left-hand scale on graph) and otic region (yellow; right-hand scale on graph). Position of the otic region was estimated by tracing the dataset backwards from a stage when the otic vesicle was evident. At 24 hpf, expression of d2EGFP in r4 decreased, whereas the otic vesicle had high Fgf activity at both the anterior and posterior poles (E–E”). The measurements at 24 hpf were taken from a different embryo using a lower laser power, resulting in different arbitrary units on the graph (E”). At earlier stages, expression of d2EGFP in the otic region was much lower than that in the rhombomeres, but a graded expression (higher at the anterior, lower at the posterior) was evident between the 8- and 16-somite stages (A–D”). The images in A–D’ were taken from the dataset shown in S1 Movie.
(TIF)

S1 Movie Time-lapse movie of a representative *Tg(dusp6*:*d2EGFP)* embryo from the 8-somite stage to the 16-somite stage. This movie was used to generate panels A–D’ in S13 Fig. Images were captured by light-sheet microscopy every 5 minutes for 4 hours (from the 8- to the 16-somite stage). Individual time points were manually drift-corrected. An average intensity projection of the fluorescent signal is shown (produced from 20 *z*-slices encompassing the otic region). A dorsal view is shown; anterior is to the top left. Scale bar, 50 μm.
(AVI)

S1 Model Details of the mathematical model. (PDF)

S1 Data Raw data for graphs in S7 and S12 Figs. (XLSX)

## References

[1] Whitfield TT, Hammond KL. Axial patterning in the developing vertebrate inner ear. Int J Dev Biol. 2007; 51 (Special Issue: Ear Development):507–20. 10.1387/ijdb.072380tw 17891713

[2] Feng Y, Xu Q. Pivotal role of *hmx2* and *hmx3* in zebrafish inner ear and lateral line development. Dev Biol. 2010; 339:507–18. 10.1016/j.ydbio.2009.12.028 20043901

[3] Kwak SJ, Vemaraju S, Moorman SJ, Zeddies D, Popper AN, Riley BB. Zebrafish *pax5* regulates development of the utricular macula and vestibular function. Dev Dyn. 2006; 235:3026–38. 10.1002/dvdy.20961 17013878

[4] Léger S, Brand M. Fgf8 and Fgf3 are required for zebrafish ear placode induction, maintenance and inner ear patterning. Mech Dev. 2002; 119:91–108. 12385757 10.1016/s0925-4773(02)00343-x

[5] McCarroll M, Nechiporuk A. Fgf3 and Fgf10a work in concert to promote maturation of the epibranchial placodes in zebrafish. PLoS One. 2013; 8:e85087 10.1371/journal.pone.0085087 24358375 PMC3866233

[6] Hammond KL, Whitfield TT. Fgf and Hh signalling act on a symmetrical pre-pattern to specify anterior and posterior identity in the zebrafish otic placode and vesicle. Development. 2011; 138:3977–87. 10.1242/dev.066639 21831919 PMC3160093

[7] Schmid B, Fürthauer M, Connors SA, Trout J, Thisse B, Thisse C, et al Equivalent genetic roles for *bmp7/snailhouse* and *bmp2b/swirl* in dorsoventral pattern formation. Development. 2000; 127:957–67. 10662635 10.1242/dev.127.5.957

[8] Mowbray C, Hammerschmidt M, Whitfield TT. Expression of BMP signalling pathway members in the developing zebrafish inner ear and lateral line. Mech Dev. 2001; 108:179–84. 11578872 10.1016/s0925-4773(01)00479-8

[9] Millimaki BB, Sweet EM, Dhason MS, Riley BB. Zebrafish *atoh1* genes: classic proneural activity in the inner ear and regulation by Fgf and Notch. Development. 2007; 134:295–305. 10.1242/dev.02734 17166920

[10] Ernest S, Rauch G-J, Haffter P, Geisler R, Petit C, Nicolson T. *Mariner* is defective in *myosin VIIA*: a zebrafish model for human hereditary deafness. Hum Mol Genet. 2000; 9:2189–96. 10958658 10.1093/hmg/9.14.2189

[11] Hammond KL, Loynes HE, Folarin AA, Smith J, Whitfield TT. Hedgehog signalling is required for correct anteroposterior patterning of the zebrafish otic vesicle. Development. 2003; 130:1403–17. 12588855 10.1242/dev.00360

[12] Hammond KL, van Eeden FJ, Whitfield TT. Repression of Hedgehog signalling is required for the acquisition of dorsolateral cell fates in the zebrafish otic vesicle. Development. 2010; 137:1361–71. 10.1242/dev.045666 20223756 PMC2847469

[13] Lecaudey V, Cakan-Akdogan G, Norton WH, Gilmour D. Dynamic Fgf signaling couples morphogenesis and migration in the zebrafish lateral line primordium. Development. 2008; 135:2695–705. 10.1242/dev.025981 18599504

[14] Millimaki BB, Sweet EM, Riley BB. Sox2 is required for maintenance and regeneration, but not initial development, of hair cells in the zebrafish inner ear. Dev Biol. 2010; 338:262–9. 10.1016/j.ydbio.2009.12.011 20025865 PMC2815045

[15] Kimmel CB, Ballard WW, Kimmel SR, Ullmann B, Schilling TF. Stages of embryonic development of the zebrafish. Dev Dyn. 1995; 203:253–310. 10.1002/aja.1002030302 8589427

[16] Phillips BT, Bolding K, Riley BB. Zebrafish *fgf3* and *fgf8* encode redundant functions required for otic placode induction. Dev Biol. 2001; 235:351–65. 10.1006/dbio.2001.0297 11437442

[17] Padanad M, Bhat N, Guo B, Riley B. Conditions that influence the response to Fgf during otic placode induction. Dev Biol. 2012; 364:1–10. 10.1016/j.ydbio.2012.01.022 22327005 PMC4709014

[18] Zou J, Guo Y, Guettouche T, Smith D, Voellmy R. Repression of heat shock transcription factor HSF1 activation by HSP90 (HSP90 complex) that forms a stress-sensitive complex with HSF1. Cell. 1998; 94:471–80. 9727490 10.1016/s0092-8674(00)81588-3

[19] Chen W, Burgess S, Hopkins N. Analysis of the zebrafish smoothened mutant reveals conserved and divergent functions of hedgehog activity. Development. 2001; 128:2385–96. 11493557 10.1242/dev.128.12.2385

[20] Varga ZM, Amores A, Lewis KE, Yan YL, Postlethwait JH, Eisen JS, et al Zebrafish *smoothened* functions in ventral neural tube specification and axon tract formation. Development. 2001; 128:3497–509. 11566855 10.1242/dev.128.18.3497

[21] Sapède D, Pujades C. Hedgehog signaling governs the development of otic sensory epithelium and its associated innervation in zebrafish. J Neurosci. 2010; 30:3612–23. 10.1523/JNEUROSCI.5109-09.2010 20219995 PMC6632237

[22] Thisse B, Thisse C. Fast release clones: a high throughput expression analysis. ZFIN Direct Data Submission (http://zfin.org). 2004.

[23] Zhang C, Li Q, Lim C, Qiu X, Jiang Y. The characterization of zebrafish antimorphic mib alleles reveals that Mib and Mind bomb-2 (Mib2) function redundantly. Dev Biol. 2007; 305:14–27. 10.1016/j.ydbio.2007.01.034 17331493

[24] Haddon C, Jiang Y-J, Smithers L, Lewis J. Delta-Notch signalling and the patterning of sensory cell differentiation in the zebrafish ear: evidence from the *mind bomb* mutant. Development. 1998; 125:4637–44. 9806913 10.1242/dev.125.23.4637

[25] Haddon C, Mowbray C, Whitfield T, Jones D, Gschmeissner S, Lewis J. Hair cells without supporting cells: further studies in the ear of the zebrafish *mind bomb* mutant. Journal of Neurocytology. 1999; 28:837–50. 10900088 10.1023/a:1007013904913

[26] Adamska M, Léger S, Brand M, Hadrys T, Braun T, Bober E. Inner ear and lateral line expression of a zebrafish *Nkx5-1* gene and its downregulation in the ears of FGF8 mutant, *ace*. Mechanisms of Development. 2000; 97:161–5. 11025218 10.1016/s0925-4773(00)00414-7

[27] Kwak S-J, Phillips BT, Heck R, Riley BB. An expanded domain of *fgf3* expression in the hindbrain of zebrafish *valentino* mutants results in mis-patterning of the otic vesicle. Development. 2002; 129:5279–87. 12399318 10.1242/dev.129.22.5279

[28] Maier E, Whitfield T. RA and FGF signalling are required in the zebrafish otic vesicle to pattern and maintain ventral otic identities. PLoS Genetics. 2014; 10:e1004858 10.1371/journal.pgen.1004858 25473832 PMC4256275

[29] Molina G, Watkins S, Tsang M. Generation of FGF reporter transgenic zebrafish and their utility in chemical screens. BMC Dev Biol. 2007; 7:62 10.1186/1471-213X-7-62 17553162 PMC1904198

[30] Bok J, Raft S, Kong KA, Koo SK, Dräger UC, Wu DK. Transient retinoic acid signaling confers anterior-posterior polarity to the inner ear. Proc Natl Acad Sci USA. 2011; 108:161–6. 10.1073/pnas.1010547108 21173260 PMC3017143

[31] Radosevic M, Robert-Moreno A, Coolen M, Bally-Cuif L, Alsina B. Her9 represses neurogenic fate downstream of Tbx1 and retinoic acid signaling in the inner ear. Development. 2011; 138:397–408. 10.1242/dev.056093 21205785

[32] Zagorski M, Tabata Y, Brandenberg N, Lutolf M, Tkačik G, Bollenbach T, et al Decoding of position in the developing neural tube from antiparallel morphogen gradients. Science. 2017; 356:1379–83. 10.1126/science.aam5887 28663499 PMC5568706

[33] Briscoe J, Small S. Morphogen rules: design principles of gradient-mediated embryo patterning. Development. 2015; 142:3996–4009. 10.1242/dev.129452 26628090 PMC4712844

[34] Anwar M, Tambalo M, Ranganathan R, Grocott T, Streit A. A gene network regulated by FGF signalling during ear development. Sci Rep. 2017; 7:6162 10.1038/s41598-017-05472-0 28733657 PMC5522468

[35] Roehl H, Nüsslein-Volhard C. Zebrafish *pea3* and *erm* are general targets of FGF8 signalling. Current Biology. 2001; 11:503–7. 11413000 10.1016/s0960-9822(01)00143-9

[36] Raible F, Brand M. Tight transcriptional control of the ETS domain factors Erm and Pea3 by Fgf signaling during early zebrafish development. Mech Dev. 2001; 107:105–17. 11520667 10.1016/s0925-4773(01)00456-7

[37] Scholpp S, Brand M. Endocytosis controls spreading and effective signaling range of Fgf8 protein. Curr Biol. 2004; 14:1834–41. 10.1016/j.cub.2004.09.084 15498491

[38] Ohta S, Wang B, Mansour S, Schoenwolf G. BMP regulates regional gene expression in the dorsal otocyst through canonical and non-canonical intracellular pathways. Development. 2016; epub ahead of print. 10.1242/dev.137133 PMC4920177 27151948

[39] Sweet EM, Vemaraju S, Riley BB. Sox2 and Fgf interact with Atoh1 to promote sensory competence throughout the zebrafish inner ear. Dev Biol. 2011; 358:113–21. 10.1016/j.ydbio.2011.07.019 21801718 PMC3171634

[40] Gou Y, Vemaraju S, Sweet E, Kwon H, Riley B. sox2 and sox3 Play unique roles in development of hair cells and neurons in the zebrafish inner ear. Dev Biol. 2018; 435:73–83. 10.1016/j.ydbio.2018.01.010 29355523 PMC5818298

[41] Wotton K, Weierud F, Juárez-Morales J, Alvares L, Dietrich S, Lewis K. Conservation of gene linkage in dispersed vertebrate NK homeobox clusters. Dev Genes Evol. 2009; 219:481–96. 10.1007/s00427-009-0311-y 20112453

[42] Wang W, Grimmer JF, Van De Water TR, T L. *Hmx2* and *Hmx3* homeobox genes direct development of the murine inner ear and hypothalamus and can be functionally replaced by *Drosophila Hmx*. Dev Cell. 2004; 7:439–53. 10.1016/j.devcel.2004.06.016 15363417

[43] Herbrand H, Guthrie S, Hadrys T, Hoffmann S, Arnold HH, Rinkwitz-Brandt S, et al Two regulatory genes, *cNkx5-1* and *cPax2*, show different responses to local signals during otic placode and vesicle formation in the chick embryo. Development. 1998; 125:645–54. 9435285 10.1242/dev.125.4.645

[44] Wang W, Van De Water T, Lufkin T. Inner ear and maternal reproductive defects in mice lacking the *Hmx3* homeobox gene. Development. 1998; 125:621–34. 9435283 10.1242/dev.125.4.621

[45] Rinkwitz-Brandt S, Justus M, Oldenettel I, Arnold H-H, Bober E. Distinct temporal expression of mouse *Nkx-5*.*1* and *Nkx-5*.*2* homeobox genes during brain and ear development. Mechanisms of Development. 1995; 52:371–81. 8541222 10.1016/0925-4773(95)00414-v

[46] Hadrys T, Braun T, Rinkwitz-Brandt S, Arnold H-H, Bober E. *Nkx5-1* controls semicircular canal formation in the mouse inner ear. Development. 1998; 125:33–9. 9389661 10.1242/dev.125.1.33

[47] Wang W, Chan EK, Baron S, Van de Water T, Lufkin T. *Hmx2* homeobox gene control of murine vestibular morphogenesis. Development. 2001; 128:5017–29. 11748138 10.1242/dev.128.24.5017

[48] Sangu N, Okamoto N, Shimojima K, Ondo Y, Nishikawa M, Yamamoto T. A de novo microdeletion in a patient with inner ear abnormalities suggests that the 10q26.13 region contains the responsible gene. Hum Genome Var. 2016; 3:16008 10.1038/hgv.2016.8 27274859 PMC4871931

[49] Miller N, Nance M, Wohler E, Hoover-Fong J, Lisi E, Thomas G, et al Molecular (SNP) analyses of overlapping hemizygous deletions of 10q25.3 to 10qter in four patients: evidence for HMX2 and HMX3 as candidate genes in hearing and vestibular function. Am J Hum Genet. 2009; 149A:669–80. 10.1002/ajmg.a.32705 PMC2743949 19253379

[50] Thisse C, Thisse B. High-resolution in situ hybridization to whole-mount zebrafish embryos. Nat Protoc. 2008; 3:59–69. 10.1038/nprot.2007.514 18193022

[51] Labun K, Montague T, Gagnon J, Thyme S, Valen E. CHOPCHOP v2: a web tool for the next generation of CRISPR genome engineering. Nuc Acids Res. 2016; 44:W272–6. 10.1093/nar/gkw398 PMC4987937 27185894

[52] Montague T, Cruz J, Gagnon J, Church G, Valen E. CHOPCHOP: a CRISPR/Cas9 and TALEN web tool for genome editing. Nuc Acids Res. 2014; 42:W401–7. 10.1093/nar/gku410 PMC4086086 24861617

[53] Gagnon J, Valen E, Thyme S, Huang P, Akhmetova L, Pauli A, et al Efficient mutagenesis by Cas9 protein-mediated oligonucleotide insertion and large-scale assessment of single-guide RNAs. PLoS One. 2014; 9:e98186 10.1371/journal.pone.0098186 24873830 PMC4038517

[54] Jao L, Wente S, Chen W. Efficient multiplex biallelic zebrafish genome editing using a CRISPR nuclease system. Proc Natl Acad Sci USA. 2013; 110:13904–9. 10.1073/pnas.1308335110 23918387 PMC3752207

[55] Kwan K, Fujimoto E, Grabher C, Mangum B, Hardy M, Campbell D, et al The Tol2kit: a multisite gateway-based construction kit for Tol2 transposon transgenesis constructs. Dev Dyn. 2007; 236:3088–99. 10.1002/dvdy.21343 17937395

[56] Schindelin J, Arganda-Carreras I, Frise E, Kaynig V, Longair M, Pietzsch T, et al Fiji: an open-source platform for biological-image analysis. Nat Meth. 2012; 9:676–82. 10.1038/nmeth.2019 PMC3855844 22743772

