# Supplementary figures and images for: Anteroposterior patterning of the zebrafish ear through Fgf- and Hh-dependent regulation of hmx3a expression
Source: PLoS Genet. 2019 Apr 25;15(4):e1008051. doi: 10.1371/journal.pgen.1008051 (PMC6504108; doi:10.1371/journal.pgen.1008051)

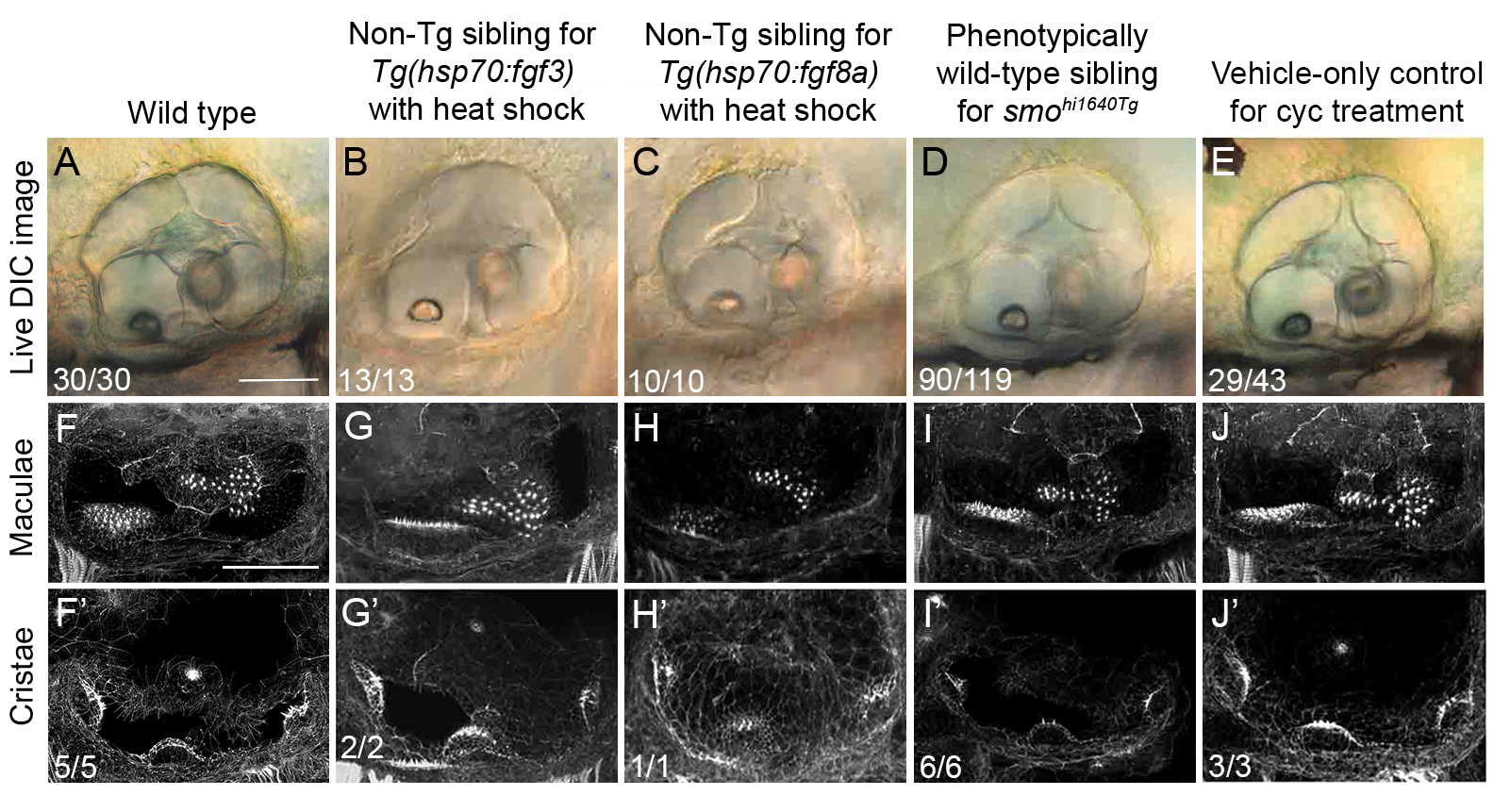

Supplement: S1 Fig — (A–E) Differential interference contrast (DIC) images of ears in live embryos at 3 dpf (72 hpf). (F–J’) Confocal images of FITC-phalloidin stains, revealing stereociliary bundles on sensory hair cells in the maculae (F–J) or cristae (F’–J’), as shown in Fig 1. The first column (‘Wild type’) repeats column 1 of Fig 1 for comparison. Subsequent columns show representative images of controls for the experiments shown in Fig 1. All ears shown were patterned normally, although views and focal planes differ slightly. All ears were of normal size and had two normally-positioned otoliths (A–E), two maculae of normal size, shape and position (F–J), and three cristae (F’–J’). Lateral views; anterior to the left. Cyc, cyclopamine. Scale bar in A, 50 μm (applies to A–E); scale bar in F, 50 μm (applies to F–J’). (TIF) [file pgen.1008051.s001.tif]

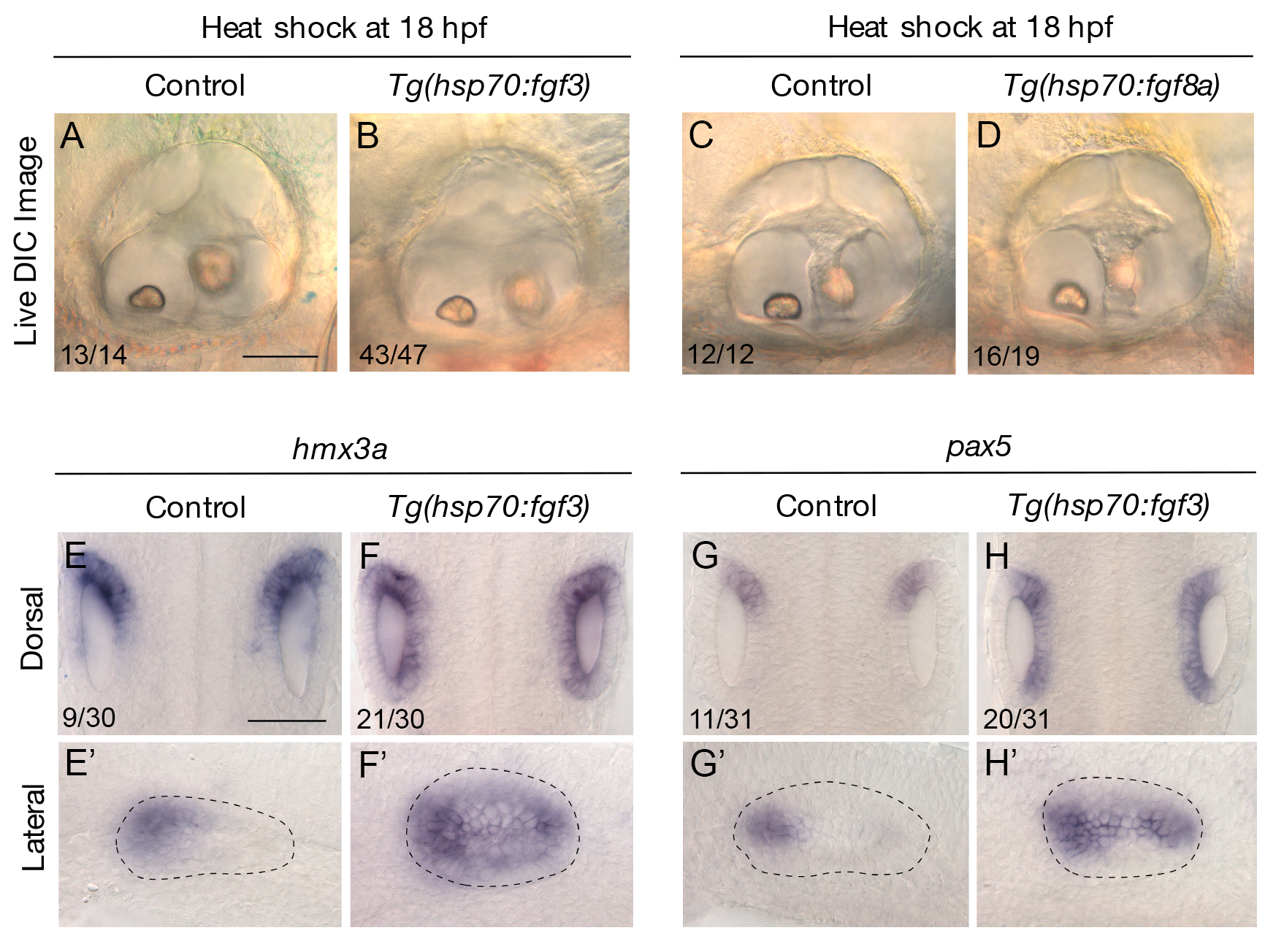

Supplement: S2 Fig — (A–D) Differential interference contrast (DIC) images of ears in live embryos at 3 dpf (72 hpf); lateral views with anterior to the left. Control embryos are non-transgenic siblings subjected to the same heat-shock treatment at 18 hpf. Representative phenotypes are shown; numbers of embryos showing the phenotype are indicated on each panel. Note the relatively normal size and shape of the ears after heat shock in transgenic animals. The focal plane for all panels is at the level of the anterior otolith; note that the posterior otolith (out of focus) is positioned dorsomedially, relative to the anterior otolith, in both control and transgenic ears. (E–H’) In situ hybridisation for hmx3a (E–F’) and pax5 (G–H’) at 22.5 hpf. Note the expansion of expression for both markers after heat shock of transgenic animals. E–H are dorsal views showing both ears; E’–H’ are lateral views with anterior to the left. Scale bar in A, 50 μm (applies to A–D); scale bar in E, 50 μm (applies to E–H’). (TIF) [file pgen.1008051.s002.tif]

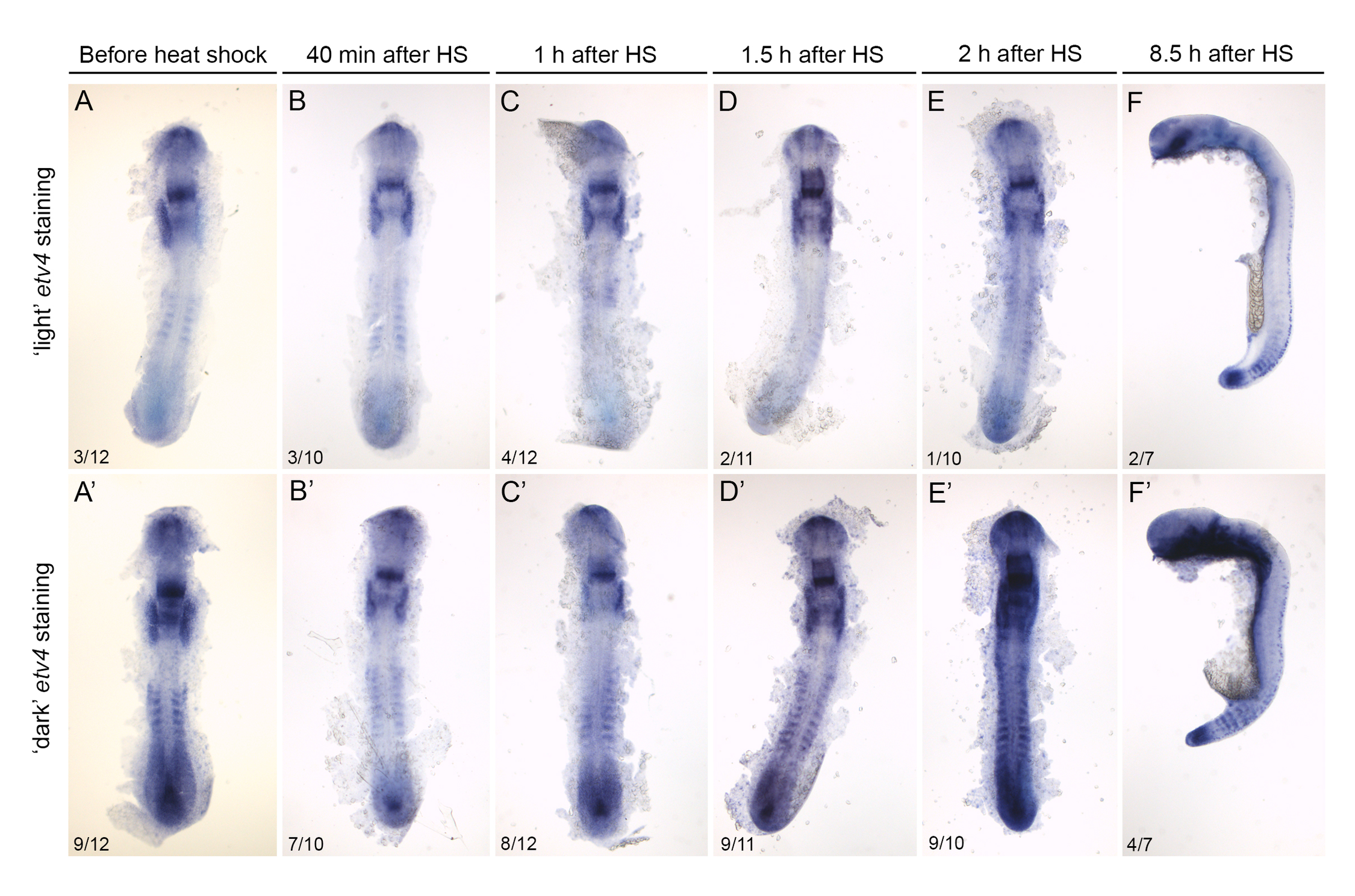

Supplement: S3 Fig — Embryos were heat-shocked for 30 minutes at 39°C at 14 hpf (the 10-somite stage), fixed at various times after the onset of heat shock as shown (top), and processed for in situ hybridisation for etv4. All embryos were stained and photographed using bright field optics under identical conditions, and scored as having ‘light’ or ‘dark’ expression (presumed transgenic and non-transgenic embryos, respectively; 75% of the batch was expected to be transgenic). Number of embryos with the phenotype shown and total number in the batch are shown directly on the panels (e.g. 3/12). Expression levels at the 10-somite stage before heat shock (A,A’) were very variable, possibly due to leaky expression of the transgene, but corresponded to the published spatial pattern of expression [22]. Robust, systemic up-regulation of etv4 was seen in embryos 2 hours after heat shock (E,E’). This persisted 8.5 hours after heat shock; here, 4/5 presumed transgenic embryos with abnormal morphology also had strong etv4 expression (F’). Morphology was normal in presumed non-transgenic siblings showing the endogenous expression pattern of etv4 (F). F,F’ show lateral views; all other panels are dorsal views of flat-mounted embryos. (TIF) [file pgen.1008051.s003.tif]

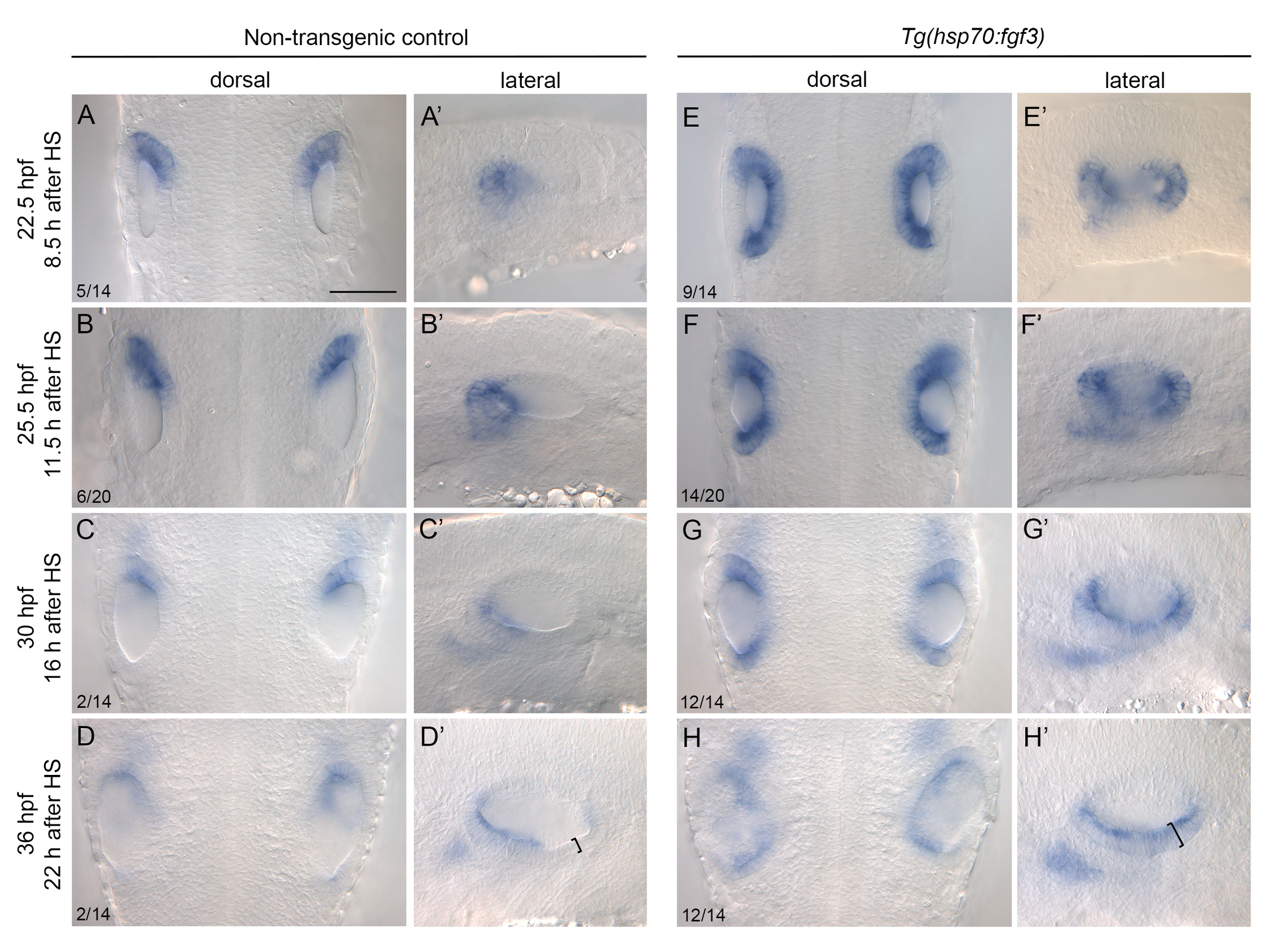

Supplement: S4 Fig — In situ hybridisation of otic expression of hmx2 in Tg(hsp70:fgf3) embryos following a 30-minute heat shock (HS) at the 10-somite stage (14 hpf). Controls (A–D’) were sibling non-transgenic embryos subjected to the same heat shock. Numbers in the dorsal view panels indicate the number of embryos with the phenotype shown and total number (e.g. 5/14) from a mixed batch of transgenic and non-transgenic embryos in each pair of panels; 75% of the batch was expected to be transgenic. The first and last rows are biological replicates of data shown in Fig 2. Note the weakening of expression in central medial otic epithelium by 25.5 hpf in transgenic embryos. By 36 hpf, in a lateral view, hmx2 is expressed throughout the ventral floor of the otic vesicle in transgenic embryos, associated with a thicker epithelium in posterolateral regions (D’, H’, brackets). All dorsal views show anterior to the top; all lateral views show anterior to the left. Scale bar in A, 50 μm (applies to all panels). (TIF) [file pgen.1008051.s004.tif]

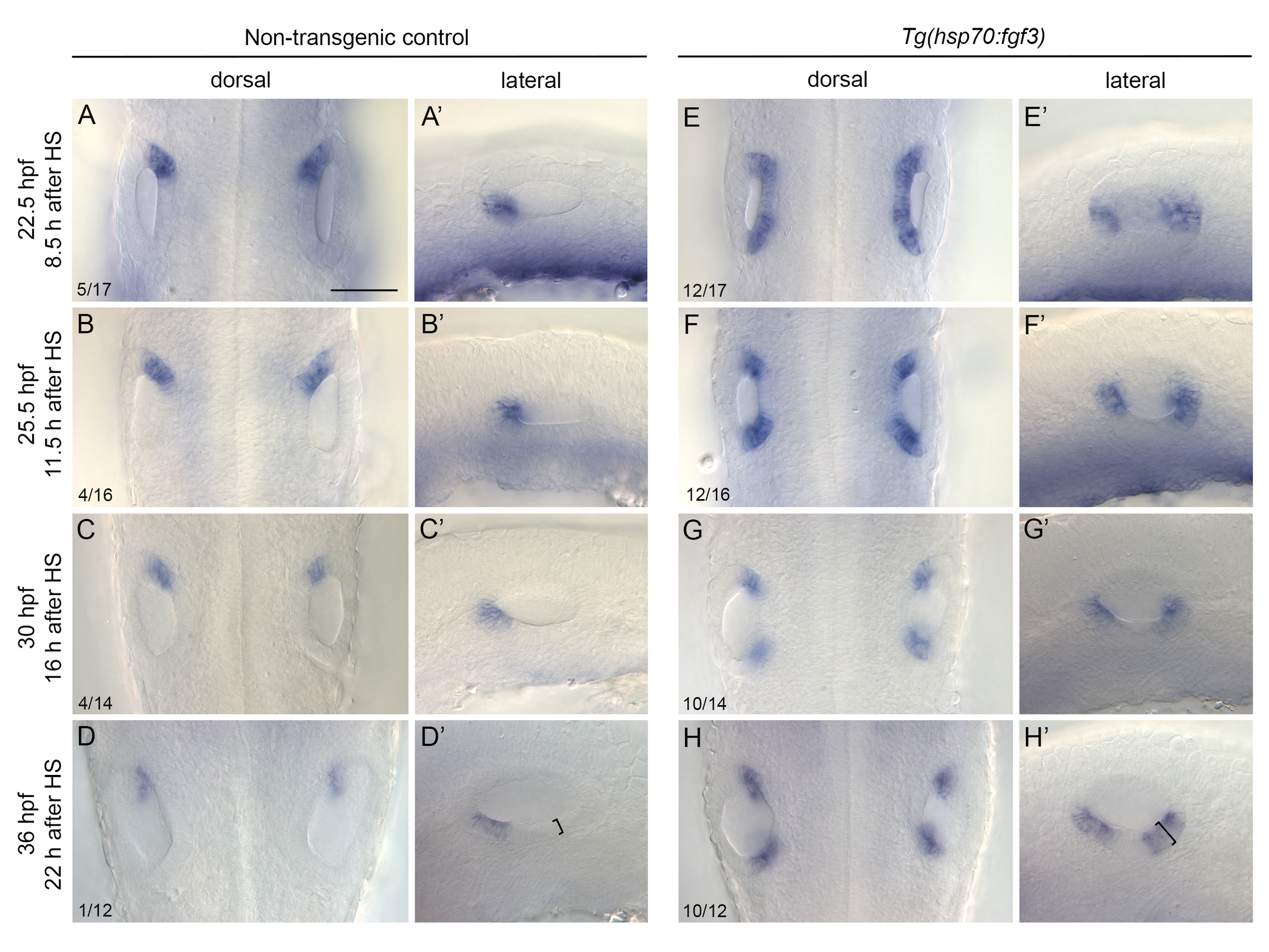

Supplement: S5 Fig — In situ hybridisation for otic expression of pax5 in Tg(hsp70:fgf3) embryos following a 30-minute heat shock (HS) at the 10-somite stage (14 hpf). Controls (A–D’) were sibling non-transgenic embryos subjected to the same heat shock. Numbers in the dorsal view panels indicate the number of embryos with the phenotype shown and total number (e.g. 5/17) from a mixed batch of transgenic and non-transgenic embryos in each pair of panels; 75% of the batch was expected to be transgenic. The first and last rows are biological replicates of data shown in Fig 2. Note that the ectopic expression in transgenic embryos has already resolved into two domains by 25.5 hpf, and two discrete domains persist at 36 hpf. At 36 hpf, in a lateral view, the ectopic domain of pax5 is associated with a thicker epithelium (D’, H’, brackets). One embryo at 36 hpf was unable to be scored. All dorsal views show anterior to the top; all lateral views show anterior to the left. Scale bar in A, 50 μm (applies to all panels). (TIF) [file pgen.1008051.s005.tif]

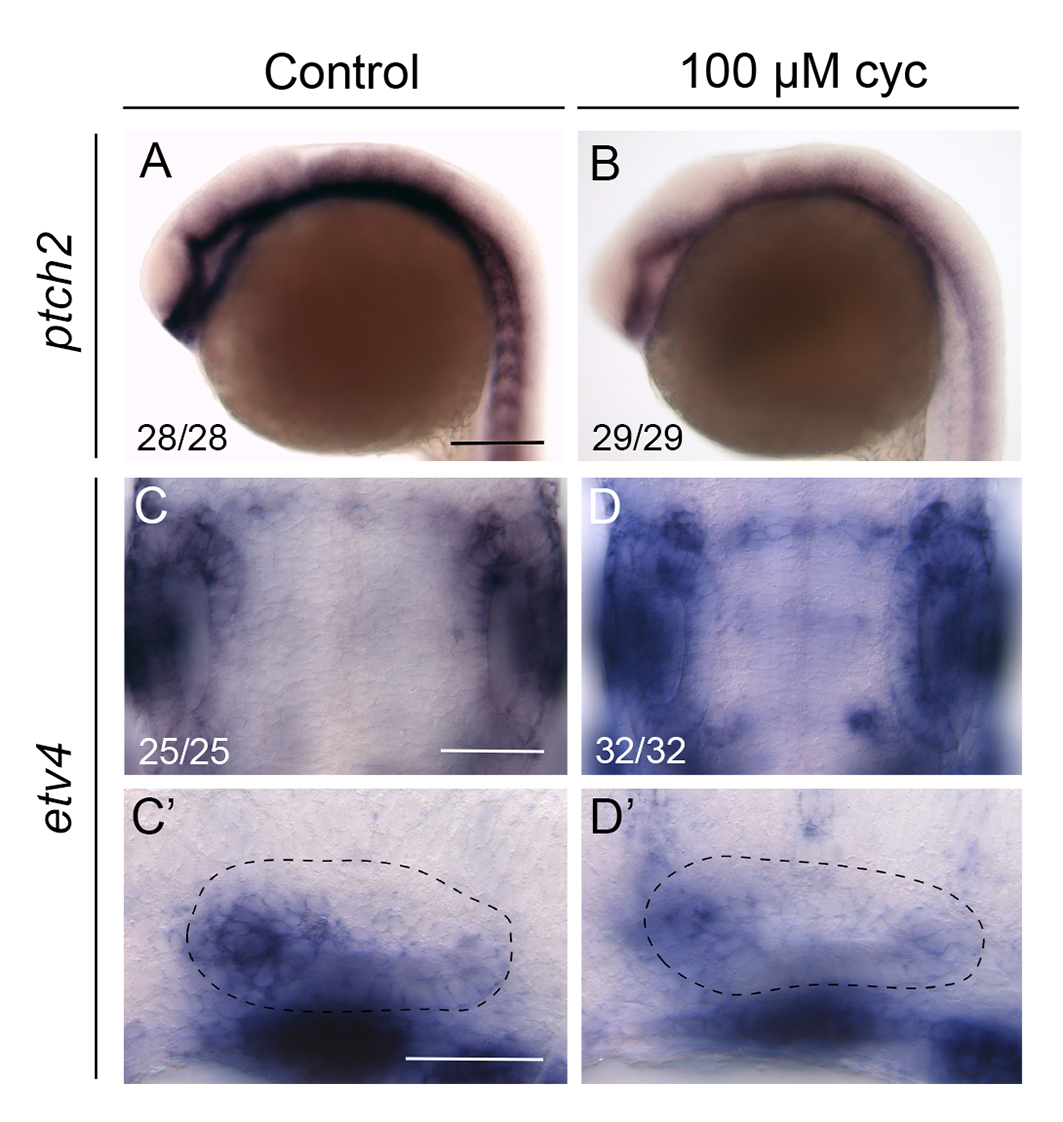

Supplement: S6 Fig — (A,B) In situ hybridisation for the Hh pathway target gene ptch2 at 22.5 hpf, after treatment from 14.5 hpf with vehicle (ethanol) only (A) or 100 μM cyclopamine (cyc; B). The head and yolk of the embryo are shown. Lateral views; anterior to the left. Expression is reduced but not abolished in cyclopamine-treated embryos (B). (C–D’) In situ hybridisation for the Fgf target gene etv4 at at 22.5 hpf, after treatment from 14.5 hpf with vehicle (ethanol) only (C,C’) or 100 μM cyclopamine (cyc; D,D’). There are no major changes to the otic expression of etv4 at this stage after cyclopamine treatment. C,D show dorsal views of the two otic vesicles with anterior to the top; C’,D’ are lateral views of the otic vesicle with anterior to the left. Scale bar in A, 200 μm (applies to B); scale bar in C, 50 μm (applies to D); scale bar in C’, 50 μm (applies to D’). (TIF) [file pgen.1008051.s006.tif]

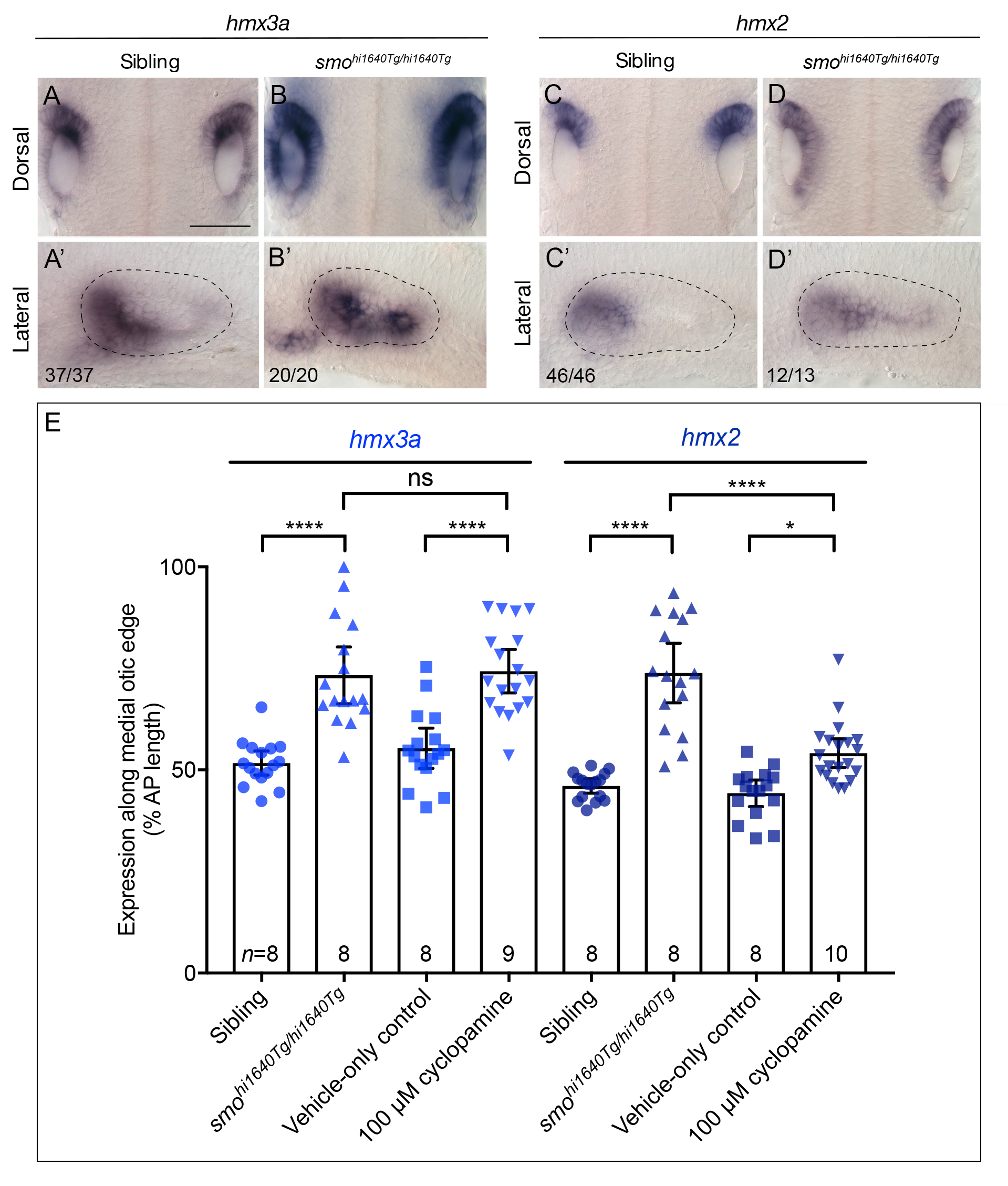

Supplement: S7 Fig — (A–D’) In situ hybridisation for hmx genes at 22.5 hpf in the otic vesicles of phenotypically wild-type and smohi1640Tg/hi1640Tg mutant embryos. In the mutant, otic expression extends posteriorly in a graded fashion at this stage. A–D show dorsal views of the two ears, with anterior to the top; A’–D’ show lateral views of the otic vesicle with anterior to the left. Scale bar in A, 50 μm (applies to A–D); scale bar in A’, 50 μm (applies to A’–D’). (E) Measurements of the extent of in situ hybridisation stain along the medial edge of the otic vesicle at 22.5 hpf, expressed as a percentage of the length of the medial edge of the otic epithelium. The expression domain of both hmx genes extends posteriorly in smohi1640Tg/hi1640Tg mutant embryos, and after treatment with 100 μM cyclopamine from 14 hpf. One-way ANOVA with Šídák’s post-test correction for multiple comparisons: ****p<0.0001; *p = 0.0119; ns, non-significant (p = 0.9998). Error bars represent the 95% confidence interval for the mean. n indicates number of embryos; two ears were measured for each embryo, with each ear measurement shown as a separate data point. See also S1 Data. (TIF) [file pgen.1008051.s007.tif]

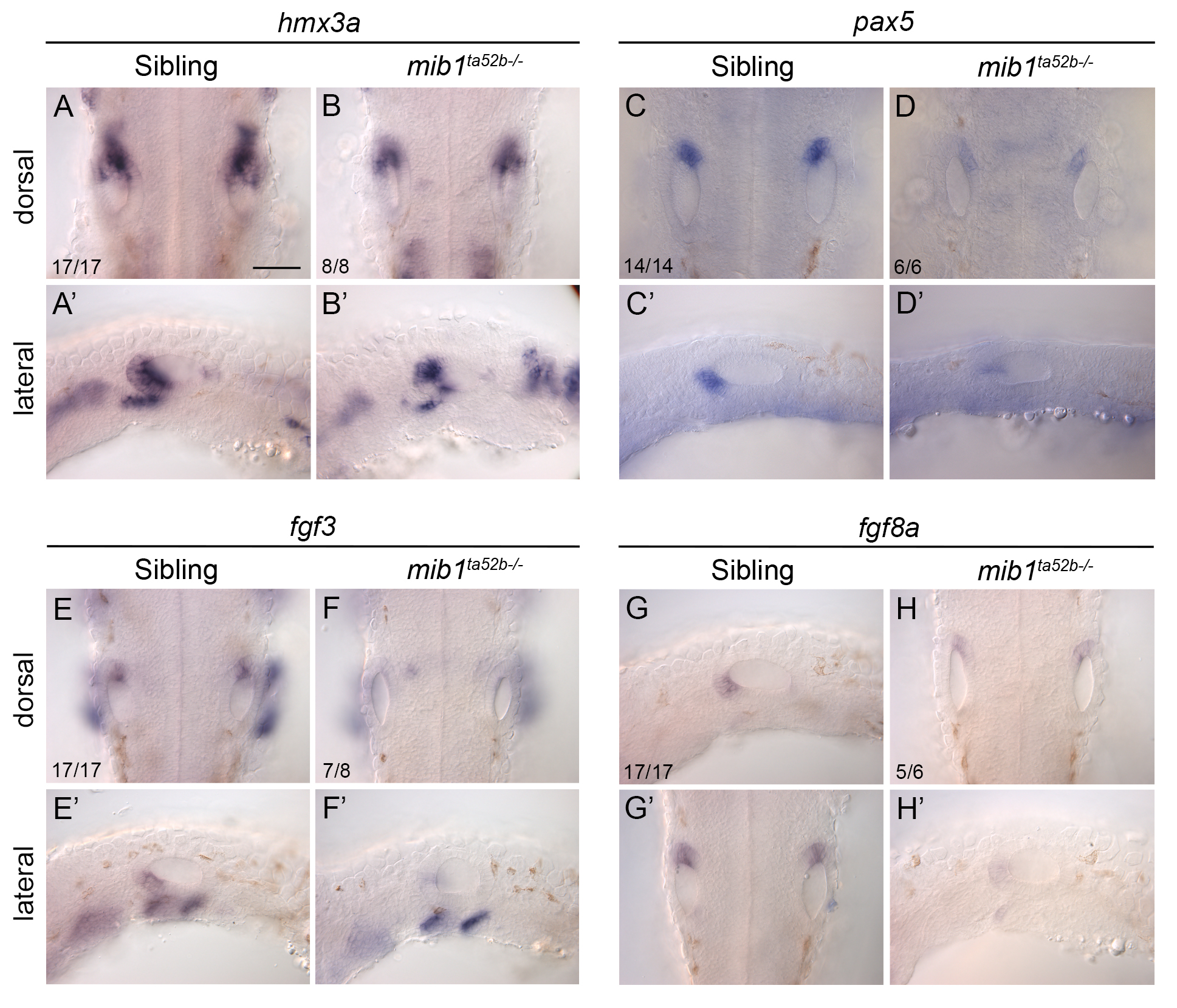

Supplement: S8 Fig — (A–H’) In situ hybridisation for anterior otic markers in phenotypically wild-type sibling (A–H) and mib1ta52b/ta52b mutant (A’–H’) ears at 25–26 hpf. All markers are expressed in the normal anterior domain in the mib1ta52b/ta52b mutant otic vesicle, but at lower levels than in sibling embryos. An anterior gap in expression is seen in the hmx3a expression domain in the mutant (B’). Expression of fgf3 in pharyngeal endoderm ventral to the ear is unaffected (F’). There is no evidence for expansion, strengthening or posterior duplicated otic expression for any of the genes tested. Numbers in the dorsal view panels indicate the number of embryos identified via morphological criteria as sibling or mutant that have the staining pattern shown. Scale bar in A, 50 μm (applies to all panels). (TIF) [file pgen.1008051.s008.tif]

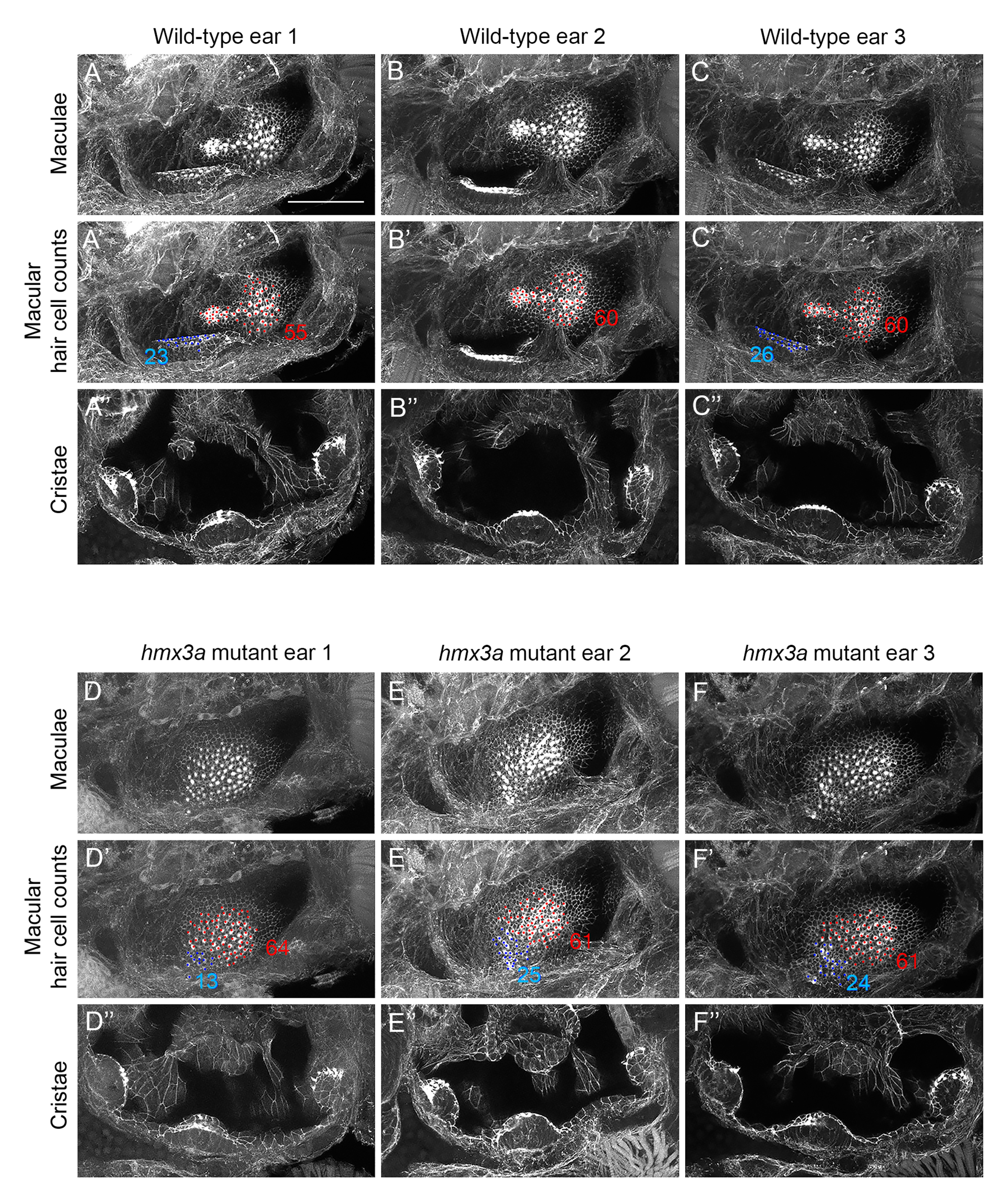

Supplement: S9 Fig — (A–F”) Confocal images of FITC-phalloidin-stained ears at 3 dpf (72 hpf); maximum intensity projections of selected z-stacks. Three ears were imaged for each genotype; note the fused maculae, but normal presence of three cristae, in each of the mutant ears (D–F”). The middle row of panels in each set is a duplicate of the panels above, with counts for visible hair cells in the anterior macula (blue) and posterior macula (red). The distinction between the anterior and posterior parts of the fused macula in panels D’–F’ was estimated based on hair cell position. Anterior macula counts in the wild-type samples are likely to be underestimates, as only some of the hair cells were visible in this orientation. It was not possible to distinguish any individual hair cells in the anterior macula in wild-type ear 2 (B’). The number of ears imaged was too small to draw firm conclusions about any changes in hair cell number in either macula. Note that the panels for wild-type ear 3 and hmx3aSU3/SU3 mutant ear 3 are reproduced in Fig 5. Scale bar, 50 μm (applies to all panels). (TIF) [file pgen.1008051.s009.tif]

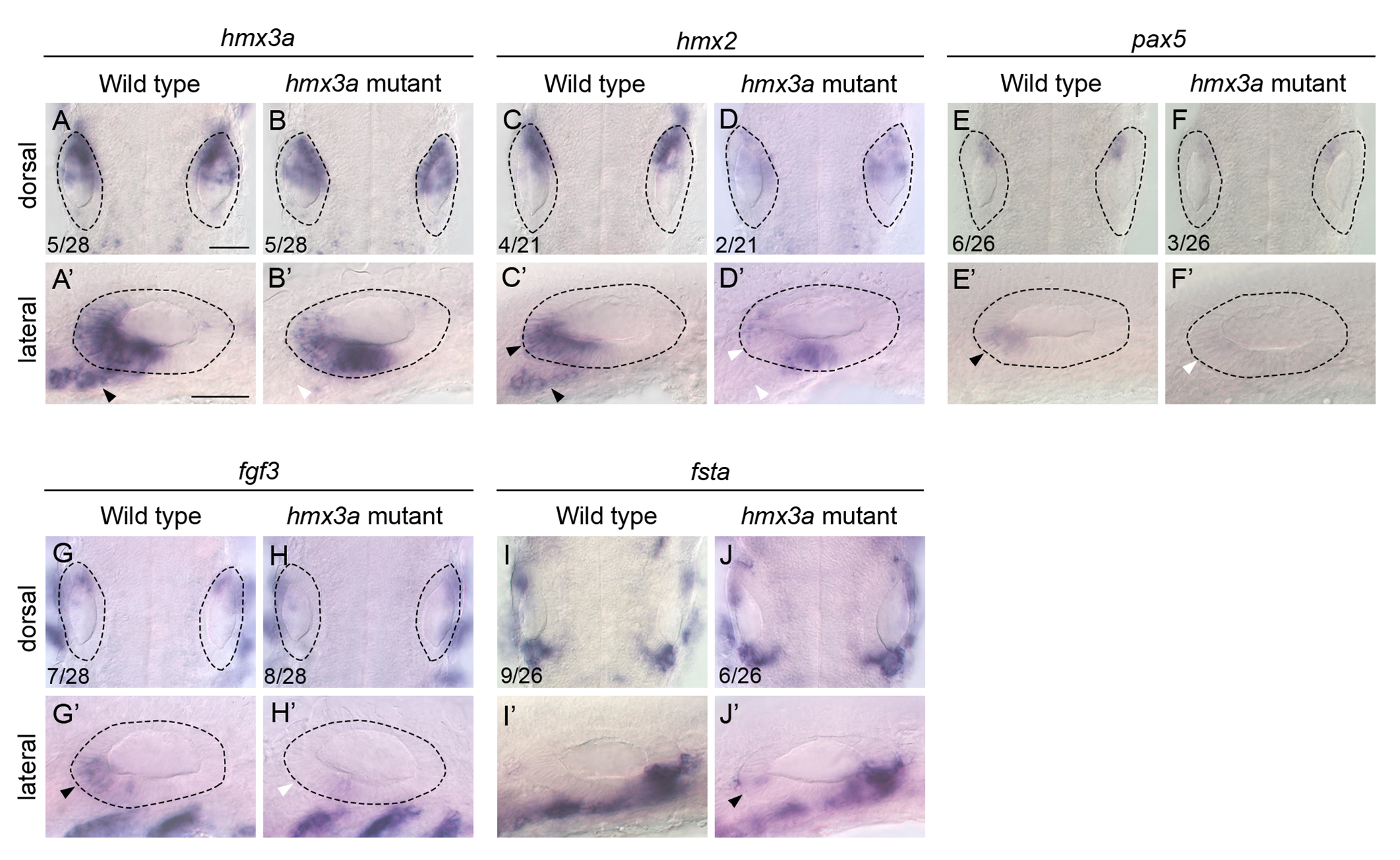

Supplement: S10 Fig — (A–H’) Reduction in expression of anterior otic markers in the otic vesicle of hmx3aSU3/SU3 mutants at 27 hpf. Black arrowheads mark expression domains in wild-type ears and in presumptive neuroblasts anteroventral to the ear that are reduced or missing in mutants (white arrowheads). (I–J’) Expression of the posterior otic marker follistatin-a (fsta) in the otic vesicle at 30 hpf. Although weak fsta expression was detected in anterior otic epithelium in mutants (J’, black arrowhead), levels were not above the natural variation seen in wild-type siblings. Numbers in panels A–J indicate numbers of embryos genotyped as either wild type or homozygous mutant that showed the representative expression patterns illustrated. Scale bar in A, 50 μm (applies to A–J); scale bar in A’, 50 μm (applies to A’–J’). (TIF) [file pgen.1008051.s010.tif]

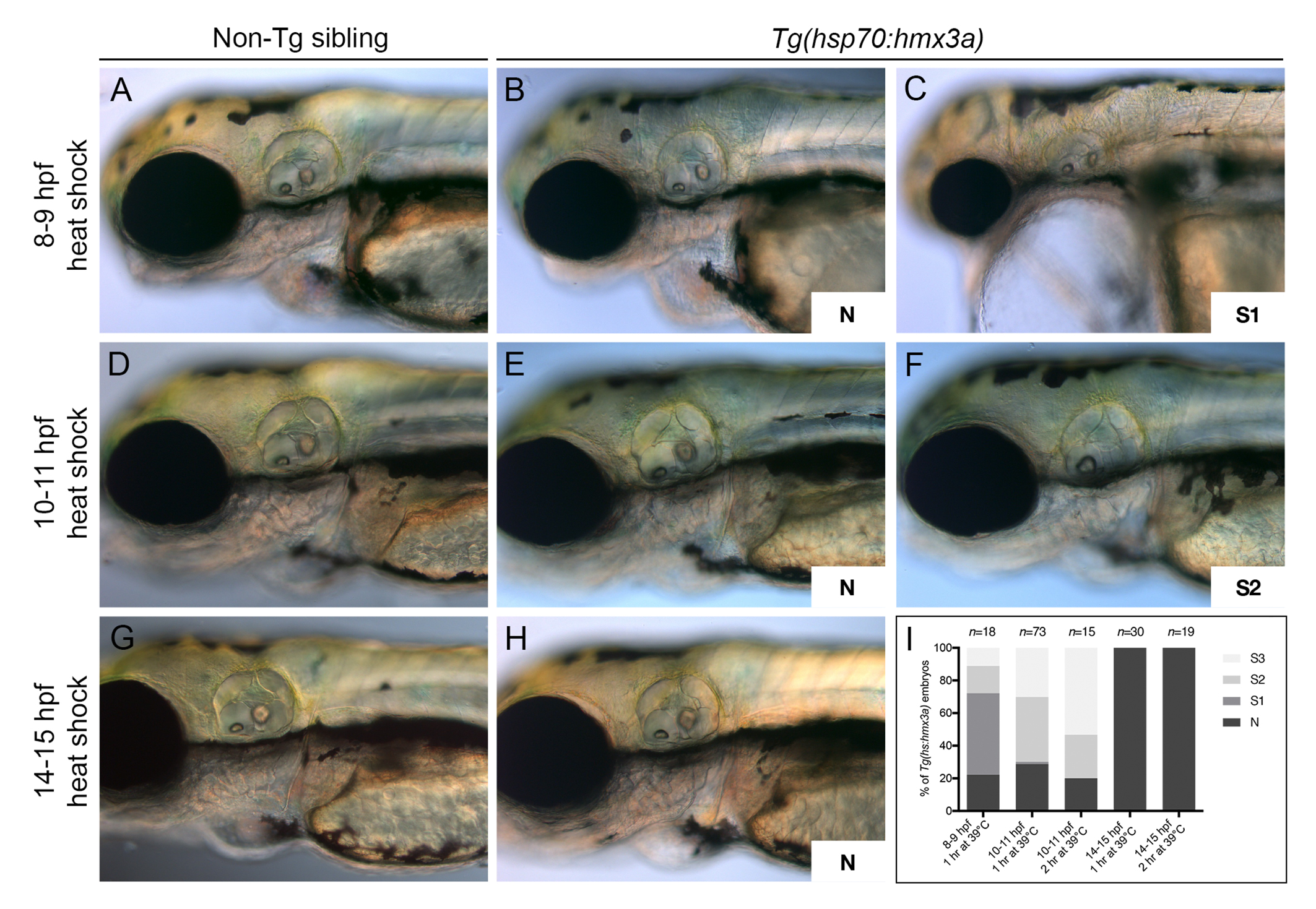

Supplement: S12 Fig — (A–H) Transgenic Tg(hsp70:hmx3a) and non-transgenic sibling embryos were heat-shocked for 1 hour at the times indicated, and grown on to 3 dpf for examination of general morphology and any otic phenotype. Representative examples are shown. (I) Graphical representation of the results from 1- and 2-hour heat shocks. Abbreviations: N, normal positioning of both anterior (ventrolateral) and posterior (posteromedial) otoliths; S1—anterior otolith in wild-type ventrolateral position but posterior otolith more ventrally positioned; S2—single otolith in one ear with two wild-type positioned otoliths in the contralateral ear; S3—single otolith in both ears; n, number of embryos. See also S1 Data. (TIF) [file pgen.1008051.s012.tif]

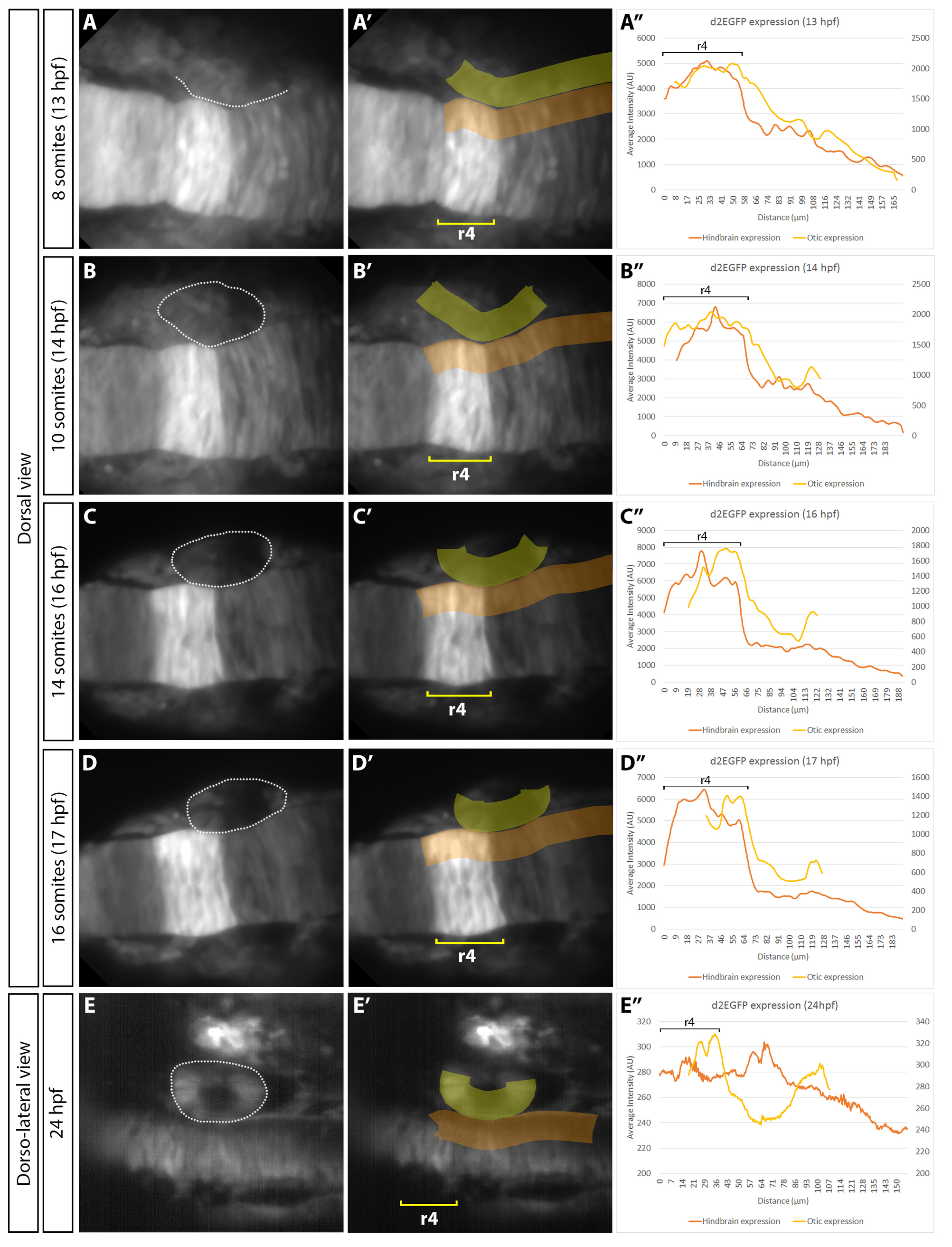

Supplement: S13 Fig — (A–E”) Light-sheet imaging and fluorescence measurements of a representative Tg(dusp6:d2EGFP) embryo from the 8-somite stage to the 16-somite stage (A–D), and a second representative embryo at 24 hpf (E). A dorsal view of the hindbrain and right-hand otic region (dotted outline) is shown; anterior is to the left. Rhombomere 4 (r4) expression is bright during the 8–16-somite stages. Expression levels were averaged over a 28.7 μm-wide band, over 20 z-sections at intervals of 1 μm, both in the hindbrain posterior to the r3/r4 boundary (orange; left-hand scale on graph) and otic region (yellow; right-hand scale on graph). Position of the otic region was estimated by tracing the dataset backwards from a stage when the otic vesicle was evident. At 24 hpf, expression of d2EGFP in r4 decreased, whereas the otic vesicle had high Fgf activity at both the anterior and posterior poles (E–E”). The measurements at 24 hpf were taken from a different embryo using a lower laser power, resulting in different arbitrary units on the graph (E”). At earlier stages, expression of d2EGFP in the otic region was much lower than that in the rhombomeres, but a graded expression (higher at the anterior, lower at the posterior) was evident between the 8- and 16-somite stages (A–D”). The images in A–D’ were taken from the dataset shown in S1 Movie. (TIF) [file pgen.1008051.s013.tif]
