# Supplementary material for: Anteroposterior patterning of the zebrafish ear through Fgf- and Hh-dependent regulation of hmx3a expression
Source: PLoS Genet. 2019 Apr 25;15(4):e1008051. doi: 10.1371/journal.pgen.1008051 (PMC6504108; doi:10.1371/journal.pgen.1008051)
Supplement: S11 Fig — Sequence of cDNA from three Tg(hsp70:hmx3a) embryos (Embryo 1–3) after heat shock, aligned to the NCBI RefSeq for zebrafish hmx3a (NM_131634.2). The start (ATG) and stop (TGA) codons of the open reading frame are shaded in pink. Exact matches with the RefSeq are shaded in black. (PDF) [file pgen.1008051.s011.pdf]

```
NM_131634.2    1 CAAGCCAACATGCCCGAAACAACACAGGATACGTGTGCTTCAGCGAAAGACTCTCCATTT
Embryo 1       1 CAAGCCAACATGCCCGAAACAACACAGGATACGTGTGCTTCAGCGAAAGACTCTCCATTT
Embryo 2       1 CAAGCCAACATGCCCGAAACAACACAGGATACGTGTGCTTCAGCGAAAGACTCTCCATTT
Embryo 3       1 CAAGCCNACATGCCCGAAACNACACAGGATACGTGTGCTTCNNCNNNAGANTCTCCATTT

NM_131634.2   61 TTCATTAAAAATCTTCTCAATTCTGACAGTAAGCCGTCAAAGCCTAAGCCTATTTTGGCA
Embryo 1      61 TTCATTAAAAATCTTCTCAATTCTGACAGTAAGCCGTCAAAGCCTAAGCCTATTTTGGCA
Embryo 2      61 TTCATTAAAAATCTTCTCAATTCTGACAGTAAGCCGTCAAAGCCTAAGCCTATTTTGGCA
Embryo 3      61 TTCATTAAAAATCTTCTCAATTCTGACAGTAAGCCGTCAAAGCCTAAGCCTATTTTGGCA

NM_131634.2  121 CCCACTAAAGCAGGACTTGATGGCAGCTTCTCCCTTTCTCAGGTTGGGGAAATAAACTTT
Embryo 1     121 CCCACTAAAGCAGGACTTGATGGCAGCTTCTCCCTTTCTCAGGTTGGGGAAATAAACTTT
Embryo 2     121 CCCACTAAAGCAGGACTTGATGGCAGCTTCTCCCTTTCTCAGGTTGGGGAAATAAACTTT
Embryo 3     121 CCCACTAAAGCAGGACTTGATGGCAGCTTCTCCCTTTCTCAGGTTGGGGAAATAAACTTT

NM_131634.2  181 CCTCGCTTTGAGTTACCCACCCAGCGCTTTGCGTTACCGGCTTACCTTGAGCGTGCCTCG
Embryo 1     181 CCTCGCTTTGAGTTACCCACCCAGCGCTTTGCGTTACCGGCTTACCTTGAGCGTGCCTCG
Embryo 2     181 CCTCGCTTTGAGTTACCCACCCAGCGCTTTGCGTTACCGGCTTACCTTGAGCGTGCCTCG
Embryo 3     181 CCTCGCTTTGAGTTACCCACCCAGCGCTTTGCGTTACCGGCTTACCTTGAGCGTGCCTCG

NM_131634.2  241 GCGTGGTGGTACCCGTACACACTCAGTGCATCCGCGCATCTTCACAGAACAGAAGCAGCA
Embryo 1     241 GCGTGGTGGTACCCGTACACACTCAGTGCATCCGCGCATCTTCACAGAACAGAAGCAGCA
Embryo 2     241 GCGTGGTGGTACCCGTACACACTCAGTGCATCCGCGCATCTTCACAGAACAGAAGCAGCA
Embryo 3     241 GCGTGGTGGTACCCGTACACACTCAGTGCATCCGCGCATCTTCACAGAACAGAAGCAGCA

NM_131634.2  301 CAGAAAGCGAGGGACTCTTCGCCGACCACAGGCACCGACCGAGATTCCCCCGAGCTTGTG
Embryo 1     301 CAGAAAGCGAGGGACTCTTCGCCGACCACAGGCACCGACCGAGATTCCCCCGAGCTTGTG
Embryo 2     301 CAGAAAGCGAGGGACTCTTCGCCGACCACAGGCACCGACCGAGATTCCCCCGAGCTTGTG
Embryo 3     301 CAGAAAGCGAGGGACTCTTCGCCGACCACAGGCACCGACCGAGATTCCCCCGAGCTTGTG

NM_131634.2  361 CTCAAATCAGATCCGGACGCCAAGGACGATGAAGACGACAACAAAAGTGGCGACGAGATT
Embryo 1     361 CTCAAATCAGATCCGGACGCCAAGGACGATGAAGACGACAACAAAAGTGGCGACGAGATT
Embryo 2     361 CTCAAATCAGATCCGGACGCCAAGGACGATGAAGACGACAACAAAAGTGGCGACGAGATT
Embryo 3     361 CTCAAATCAGATCCGGACGCCAAGGACGATGAAGACGACAACAAAAGTGGCGACGAGATT

NM_131634.2  421 GTCCTCGAGGAGAGCGACACCGAAGACGGTAAAAAAGAAGGCGGCATAGACGACTGGAAG
Embryo 1     421 GTCCTCGAGGAGAGCGACACCGAAGACGGTAAAAAAGAAGGCGGCATAGACGACTGGAAG
Embryo 2     421 GTCCTCGAGGAGAGCGACACCGAAGACGGTAAAAAAGAAGGCGGCATAGACGACTGGAAG
Embryo 3     421 GTCCTCGAGGAGAGCGACACCGAAGACGGTAAAAAAGAAGGCGGCATAGACGACTGGAAG

NM_131634.2  481 AAGAGTGACGACGGCGCTGACAAGAAACCTTGCCGGAAAAAGAAAACTCGCACGGTGTTC
Embryo 1     481 AAGAGTGACGACGGCGCTGACAAGAAACCTTGCCGGAAAAAGAAAACTCGCACGGTGTTC
Embryo 2     481 AAGAGTGACGACGGCGCTGACAAGAAACCTTGCCGGAAAAAGAAAACTCGCACGGTGTTC
Embryo 3     481 AAGAGTGACGACGGCGCTGACAAGAAACCTTGCCGGAAAAAGAAAACTCGCACGGTGTTC

NM_131634.2  541 TCGCGGAGTCAGGTGTTCCAGCTGGAGTCCACCTTCGACATGAAACGCTACCTCAGCAGC
Embryo 1     541 TCGCGGAGTCAGGTGTTCCAGCTGGAGTCCACCTTCGACATGAAACGCTACCTCAGCAGC
Embryo 2     541 TCGCGGAGTCAGGTGTTCCAGCTGGAGTCCACCTTCGACATGAAACGCTACCTCAGCAGC
Embryo 3     541 TCGCGGAGTCAGGTGTTCCAGCTGGAGTCCACCTTCGACATGAAACGCTACCTCAGCAGC

NM_131634.2  601 TCGGAGCGCGCGGGCCTTGCTGCCTCCCTTCACCTCACAGAGACTCAAGTGAAAATCTGG
Embryo 1     601 TCGGAGCGCGCGGGCCTTGCTGCCTCCCTTCACCTCACAGAGACTCAAGTGAAAATCTGG
Embryo 2     601 TCGGAGCGCGCGGGCCTTGCTGCCTCCCTTCACCTCACAGAGACTCAAGTGAAAATCTGG
Embryo 3     601 TCGGAGCGCGCGGGCCTTGCTGCCTCCCTTCACCTCACAGAGACTCAAGTGAAAATCTGG

NM_131634.2  661 TTTCAGAACCGCAGAAACAAATGGAAACGTCAGCTGGCCGCAGAGCTGGAGGCCGCCAAC
Embryo 1     661 TTTCAGAACCGCAGAAACAAATGGAAACGTCAGCTGGCCGCAGAGCTGGAGGCCGCCAAC
Embryo 2     661 TTTCAGAACCGCAGAAACAAATGGAAACGTCAGCTGGCCGCAGAGCTGGAGGCCGCCAAC
Embryo 3     661 TTTCAGAACCGCAGAAACAAATGGAAACGTCAGCTGGCCGCAGAGCTGGAGGCCGCCAAC

NM_131634.2  721 TTGAGCCACGCAGCAGCACAAAGGATTGTGAGAGTACCCATCCTGTATCACGAGAACTCG
Embryo 1     721 TTGAGCCACGCAGCAGCACAAAGGATTGTGAGAGTACCCATCCTGTATCACGAGAACTCG
Embryo 2     721 TTGAGCCACGCAGCAGCACAAAGGATTGTGAGAGTACCCATCCTGTATCACGAGAACTCG
Embryo 3     721 TTGAGCCACGCAGCAGCACAAAGGATTGTGAGAGTACCCATCCTGTATCACGAGAACTCG

NM_131634.2  781 GCCTCTGAGAGCACCAACACAGCGGGCAACGTTCCTGTAAGCCAGCCGCTGCTCACTTTC
Embryo 1     781 GCCTCTGAGAGCACCAACACAGCGGGCAACGTTCCTGTAAGCCAGCCGCTGCTCACTTTC
Embryo 2     781 GCCTCTGAGAGCACCAACACAGCGGGCAACGTTCCTGTAAGCCAGCCGCTGCTCACTTTC
Embryo 3     781 GCCTCTGAGAGCACCAACACAGCGGGCAACGTTCCTGTAAGCCAGCCGCTGCTCACTTTC

NM_131634.2  841 CCTCATCCGGTTTACTACTCGCACCCCATCGTCACCTCCGTGCCCCTCCTCAGACCGGTT
Embryo 1     841 CCTCATCCGGTTTACTACTCGCACCCCATCGTCACCTCCGTGCCCCTCCTCAGACCGGTT
Embryo 2     841 CCTCATCCGGTTTACTACTCGCACCCCATCGTCACCTCCGTGCCCCTCCTCAGACCGGTT
Embryo 3     841 CCTCATCCGGTTTACTACTCGCACCCCATCGTCACCTCCGTGCCCCTCCTCAGACCGGTT

NM_131634.2  901 TGAGAGGACGACAGATGT
Embryo 1     901 TGAGAGGACGACAGATGT
Embryo 2     901 TGAGAGGACGACAGATGT
Embryo 3     901 TGAGAGGACGACAGATGT
```
