# Supplementary material for: Anteroposterior patterning of the zebrafish ear through Fgf- and Hh-dependent regulation of hmx3a expression
Source: PLoS Genet. 2019 Apr 25;15(4):e1008051. doi: 10.1371/journal.pgen.1008051 (PMC6504108; doi:10.1371/journal.pgen.1008051)
Supplement: S1 Model — (PDF) [file pgen.1008051.s015.pdf]

Anteroposterior patterning of the zebrafish ear through Fgf- and Hh-dependent regulation of *hmx3a* expression

Ryan D. Hartwell, Samantha J. England, Nicholas A. M. Monk, Nicholas J. van Hateren, Sarah Baxendale, Mar Marzo, Katharine E. Lewis and Tanya T. Whitfield

S1 Mathematical Model

Representation of otic tissue

For the purposes of modelling gene expression in the developing otic tissue between 14 and 36 hours post fertilisation (hpf), we represent the medial side of the otic tissue as a one-dimensional array of cells. Distance along this array—represented by the variable x—is measured in percentage length along the anterior-posterior (AP) axis. At the stages studied, the length of the medial side is approximately 100μm.

Competence to express *fgf*

Our data suggest that competence to express *fgf* in the otic tissue (fgf_i) begins at around 14 hpf (see Fig 7B) and is strongest at the poles. We therefore assume that this competence can be represented by a function of the form

$$C(x,t) = \begin{cases} 0 & \text{if } t \leq 14 \text{ hpf}, \\ 0.15 + 0.85\left(\dfrac{x_c^m}{x_c^m + x^m} + \dfrac{x_c^m}{x_c^m + (100-x)^m}\right) & \text{if } t > 14 \text{ hpf}, \end{cases} \tag{1}$$

where $0 \leq x \leq 100$ is percentage length along the AP axis, x_c is a measure of the extent of the polar competence regions, and m is a measure of the sharpness of the boundaries between regions of high and low competence. In our model simulations, we assume $x_c = 20\%$ AP length and $m = 5$, giving the competence profile shown in Fig S1A.

Extrinsic Fgf expression

We assume that rhombomere 4 of the hindbrain acts as the main source of extrinsic Fgf signalling; both *fgf3* and *fgf8a* are expressed here at the time of initial otic anteroposterior patterning (Maves et al., 2002). Further support is provided by analysis of mutants for *mafba* (Kwak et al., 2002) and *hnf1ba* (Lecaudey et al., 2007). These two genes code for transcription factors expressed in the hindbrain and are required for restriction of *fgf3* expression to rhombomere 4. In the *mafba*$^{-/-}$ and *hnf1ba*$^{-/-}$ mutants, posterior

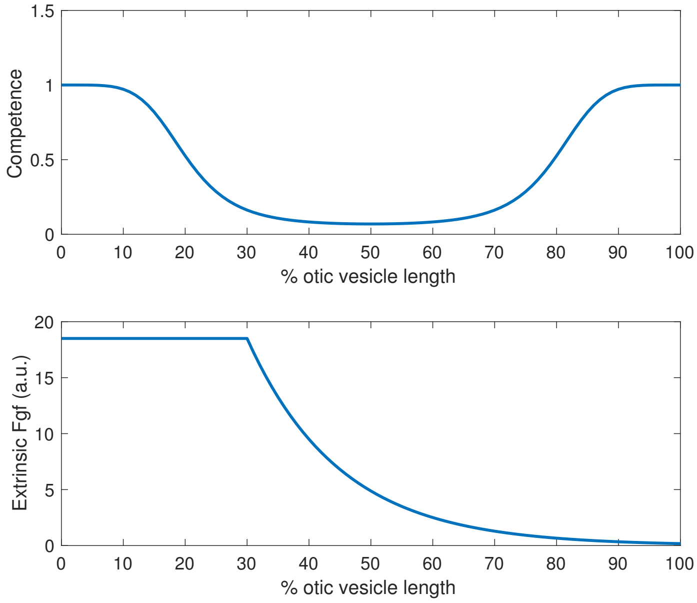

Figure S1: (A) Assumed distribution of competence for endogenous *fgf* expression in the otic tissue with $x_c = 20\%$ AP length and $m = 5$ (see Eq. (1)); (B) Assumed distribution (up until 18 hpf) in the otic tissue of Fgf protein produced in rhombomere 4 (see Eq. (2)). $F_0 = 18.5$, $x_0 = 30\%$ AP axis, $\lambda_f = 15\%$ AP axis.

expansions of *fgf3* expression in the hindbrain correlate with expansions or duplications of otic anterior markers similar to those we see in *Tg(hsp70:fgf3)* embryos after heat shock.

We assume that the extrinsic Fgf3 protein spreads away from the cells in which it is produced, resulting in a graded expression profile in the neighbouring otic tissue. In support of this, we show in S13 Fig that a reporter of Fgf signalling activity is expressed in a decreasing gradient in the otic region, with highest activity in the tissue neighbouring rhombomere 4. Up to time $t = 18$ hpf, we assume that the distribution of rhombomeric Fgf protein in the otic tissue is given by

$$F_r(x) = \begin{cases} F_0 & \text{if } x \leq x_0, \\ F_0 \exp(-(x - x_0)/\lambda_f) & \text{if } x > x_0, \end{cases} \tag{2}$$

where $F_r(x)$ is the Fgf protein concentration (in arbitrary units) in the otic tissue at AP position x, $0 \leq x \leq x_0$ is the region of overlap between rhombomere 4 and the otic tissue, F_0 is the maximum protein concentration, and λ_f is the effective "diffusion wavelength" of Fgf. The resulting concentration profile is shown in Fig S1B for $F_0 = 18.5$, $x_0 = 30\%$ AP axis, and $\lambda_f = 15\%$ AP axis (approximately 15 μm at the stages studied).
fgf expression in rhombomere 4 decreases significantly at around 18 hpf (Maves *et al.*, 2002). Up until this time, the Fgf concentration profile in the otic tissue is given by Eq. (2). At times later than 18 hpf, we assume linear degradation of the Fgf protein. The Fgf concentration at time t is therefore given by

$$F_e(x, t) = \begin{cases} F_r(x) & \text{if } t \leq 18 \text{ hpf}, \\ F_r(x) \exp(-(t - 18)/\tau_e) & \text{if } t > 18 \text{ hpf}, \end{cases} \tag{3}$$

where τ_e is the half-life (in hours) of the Fgf protein.

Regulated gene expression in the otic tissue

Fgf protein originating from rhombomere 4 initiates a temporal sequence of spatially patterned gene expression in the otic tissue. Based on the inferred interactions summarised in Fig 7A, we represent transcription and translation using a system of coupled differential equations as follows:

$$\frac{\partial h_m}{\partial t} = k\left(f(F_t, R; \theta_1, \rho_1) + g(H_p; \theta_{10})\right) - \mu_1 h_m, \quad (4)$$

$$\frac{\partial H_p}{\partial t} = \kappa h_m - \nu_1 H_p, \quad (5)$$

$$\frac{\partial p_m}{\partial t} = k f(F_t, R; \theta_2, \rho_2)\ g(H_p; \theta_3) - \mu_2 p_m, \quad (6)$$

$$\frac{\partial f_{3m}}{\partial t} = C(x,t)\ k f(F_t, R; \theta_4, \rho_4)\ g(H_p; \theta_5) - \mu_4 f_{3m}, \quad (7)$$

$$\frac{\partial f_{8m}}{\partial t} = C(x,t)\ k f(F_t, R; \theta_6, \rho_6)\ \left[\beta_7 + g(H_p; \theta_7)\right] - \mu_6 f_{8m}, \quad (8)$$

$$\frac{\partial f_{10m}}{\partial t} = C(x,t)\ k f(F_t, R; \theta_8, \rho_8)\ \left[\beta_9 + g(H_p; \theta_9)\right] - \mu_8 f_{10m}, \quad (9)$$

$$\frac{\partial F_{3p}}{\partial t} = \kappa f_{3m} - \nu_2 F_{3p} + D\frac{\partial^2 F_{3p}}{\partial x^2}, \quad (10)$$

$$\frac{\partial F_{8p}}{\partial t} = \kappa f_{8m} - \nu_3 F_{3p} + D\frac{\partial^2 F_{8p}}{\partial x^2}, \quad (11)$$

$$\frac{\partial F_{10p}}{\partial t} = \kappa f_{10m} - \nu_4 F_{3p} + D\frac{\partial^2 F_{10p}}{\partial x^2}, \quad (12)$$

where

$$F_t = F_e + F_{3p} + F_{8p} + F_{10p} \quad (13)$$

represents the total amount of Fgf protein in the otic tissue and the transcription regulation functions are given by increasing sigmoid (Hill) functions of the general form:

$$f(F_t, R; \theta, \rho) = \frac{F_t^2}{(\theta + \rho R)^2 + F_t^2}, \quad (14)$$

$$g(H_p; \theta) = \frac{H_p^2}{\theta^2 + H_p^2}. \quad (15)$$

In these functions, the parameter θ represents the activation threshold — the concentration of activator required to achieve a half-maximal rate of transcription. The effect of Hh attenuation is to increase the threshold in Eq. (14) by an amount ρR, where R is a measure of the amount of Hh signalling in the otic tissue, and ρ is the relative attenuation strength for each gene. Hh attenuation thus reduces the rate of transcription resulting from a given concentration of Fgf.

The meaning of all model variables and parameters is summarised in Tables S1–S3.

Variable	*Meaning*
h_m	*hmx3a* mRNA concentration
H_p	Hmx3a protein concentration
p_m	*pax5* mRNA concentration
f_{3m}	*fgf3* mRNA concentration
f_{8m}	*fgf8a* mRNA concentration
f_{10m}	*fgf10a* mRNA concentration
F_{3p}	Fgf3 protein concentration
F_{8p}	Fgf8a protein concentration
F_{10p}	Fgf10a protein concentration
F_e	Extrinsic Fgf protein concentration
F_t	Total Fgf protein concentration
R	Hh protein concentration
$C(x,t)$	Competence to express *fgf*
$F_e(x,t)$	Extrinsic Fgf protein

Table S1: Definition of model variables

In the model, expression of all genes is activated by the total amount of Fgf protein (both extrinsic and that produced within the otic tissue) and attenuated by Hh protein. Expression of *hmx3a*, *pax5*, *fgf3*, *fgf8a* and *fgf10a* is additionally activated by Hmx3a protein.

Simulations of the model were performed with the parameter values listed in Tables S2 and S3 on a discrete spatial domain comprising 100 spatial cells (with the diffusion terms in Eqs. (10)–(12) represented by a simple finite difference scheme), with zero-flux boundary conditions at the anterior and posterior poles of the otic vesicle. Simulations covered the time period 10–36 hpf, with initial values of all intrinsic mRNA and protein variables set to zero. The resulting spatiotemporal mRNA expression patterns are shown in Fig. 8, for three conditions: wild type (1st column), heat shock induction of *fgf3* (2nd column), and inhibition of Hh signalling by cyclopamine treatment (3rd column). The simulation protocols for the latter two conditions are described below. Fig S2 shows the spatial profiles of mRNA and total Fgf protein expression for the three conditions at 22.5 hpf and 36 hpf.

Transcription and translation rates for all endogenous genes and proteins have been set to 1, so all expression levels are expressed in arbitrary units. Half-lives of mRNA and protein have been set to reflect the observed dynamics of expression patterns. For example, the

pax5 mRNA half life is set to be low (0.5 hrs) to reflect the fact that *pax5* mRNA expression induced in the middle part of the otic vesicle by heat shock Fgf3 protein is lost by 36 hpf. The *hmx3a* mRNA half life is also set to be low (0.5 hrs) to reflect the early onset of *hmx3a* expression. In contrast, the *fgf* mRNA half lives are set to be higher to reflect the later onset of their expression. The Fgf protein diffusion coefficient is set to be low in order to avoid Fgf protein produced in the anterior and posterior poles “flooding” the otic vesicle. The short half lives of the Fgf proteins also contribute to the restriction of Fgf proteins to the poles. Indeed, Fgf protein diffusion can be omitted from the model without affecting the dynamics of the mRNA expression patterns.

The transcription regulation parameters, which reflect the level of expression at which regulating proteins effect regulation of their targets, were chosen with reference to the expression levels achieved by each protein in the model (shown in Fig S3). For example, the threshold for regulation of *hmx3a* expression by Fgf protein (θ_1) is the primary determinant of the extent of the anterior expression domain of *hmx3a* mRNA.

Parameter	*Value*	*Description*
k	1 a.u. per hr	Maximum transcription rate
κ	1 a.u. per mRNA per hr	Translation rate
k_{HS}	100 a.u. per hr	*fgf3* heat shock transcription rate
μ_1	$\ln 2/0.5$ hr^{-1}	*hmx3a* degradation rate
μ_2	$\ln 2/0.5$ hr^{-1}	*pax5* degradation rate
μ_4	$\ln 2/3$ hr^{-1}	*fgf3* degradation rate
μ_6	$\ln 2/6$ hr^{-1}	*fgf8a* degradation rate
μ_8	$\ln 2/4$ hr^{-1}	*fgf10a* degradation rate
ν_1	$\ln 2/1.5$ hr^{-1}	Hmx3a degradation rate
ν_2	$\ln 2/0.5$ hr^{-1}	Fgf3 degradation rate
ν_3	$\ln 2/0.5$ hr^{-1}	Fgf8a degradation rate
ν_4	$\ln 2/0.5$ hr^{-1}	Fgf10a degradation rate
D	50 μm^2 hr^{-1}	Fgf diffusion coefficient
F_0	18.5	Extrinsic Fgf amount (a.u.)
R	15	Hh attenuation strength

Table S2: Production, degradation and diffusion parameters

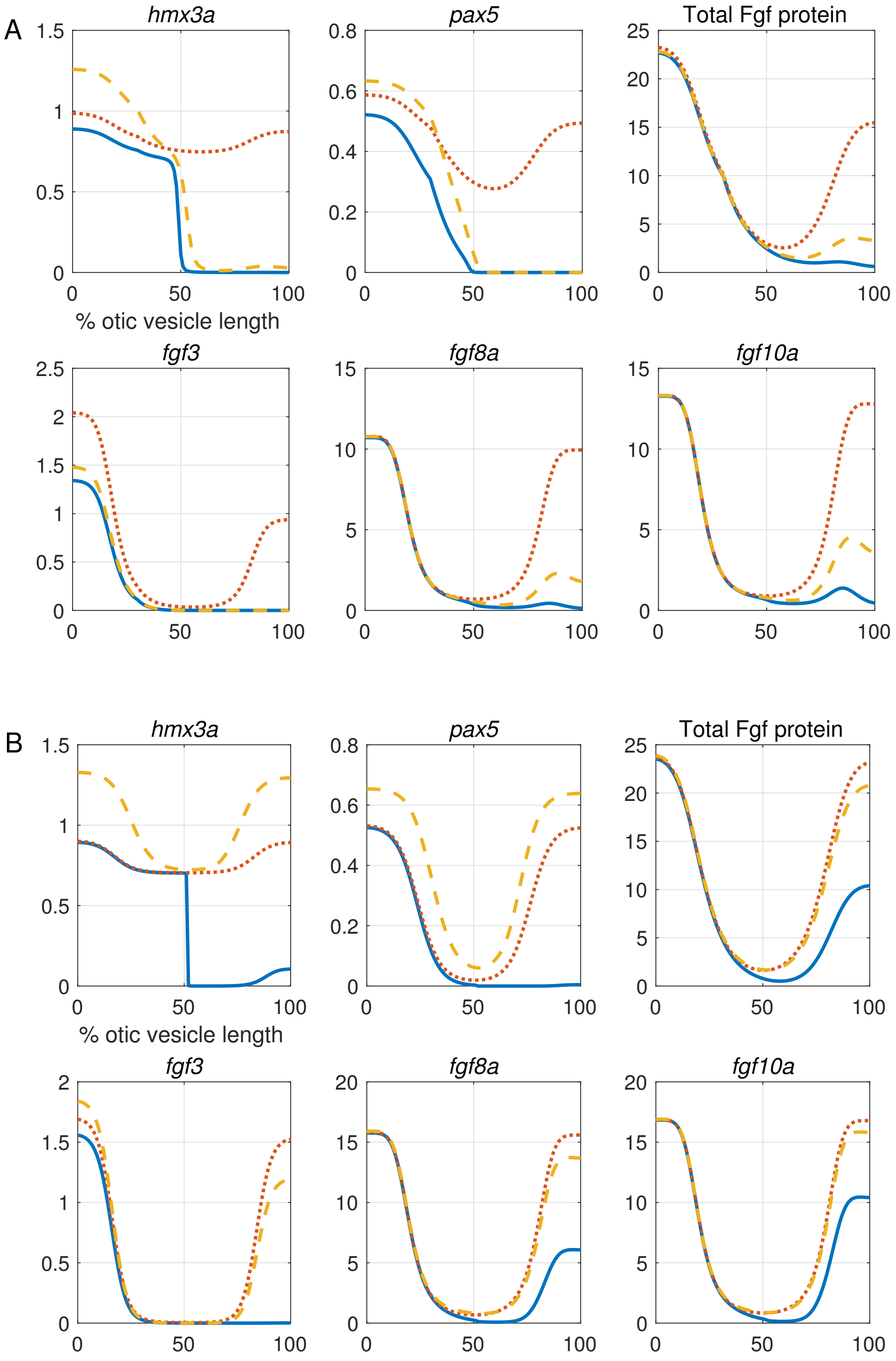

Figure S2: Spatial expression profiles of model variables at 22.5 hpf (A) and 36 hpf (B). In each panel, the solid blue curve represents the wild type, the dotted red line represents heat shock induction of *fgf3*, and the dashed orange line represents cyclopamine treatment (inhibition of Hh signalling). mRNA profiles (*hmx3a*, *pax5*, *fgf3*, *fgf8a*, *fgf10a*) are measured as transcripts per cell; Fgf protein is measured as protein molecules per cell, and "Total Fgf protein" represents F_t — the sum of all Fgf proteins (intrinsic and extrinsic).

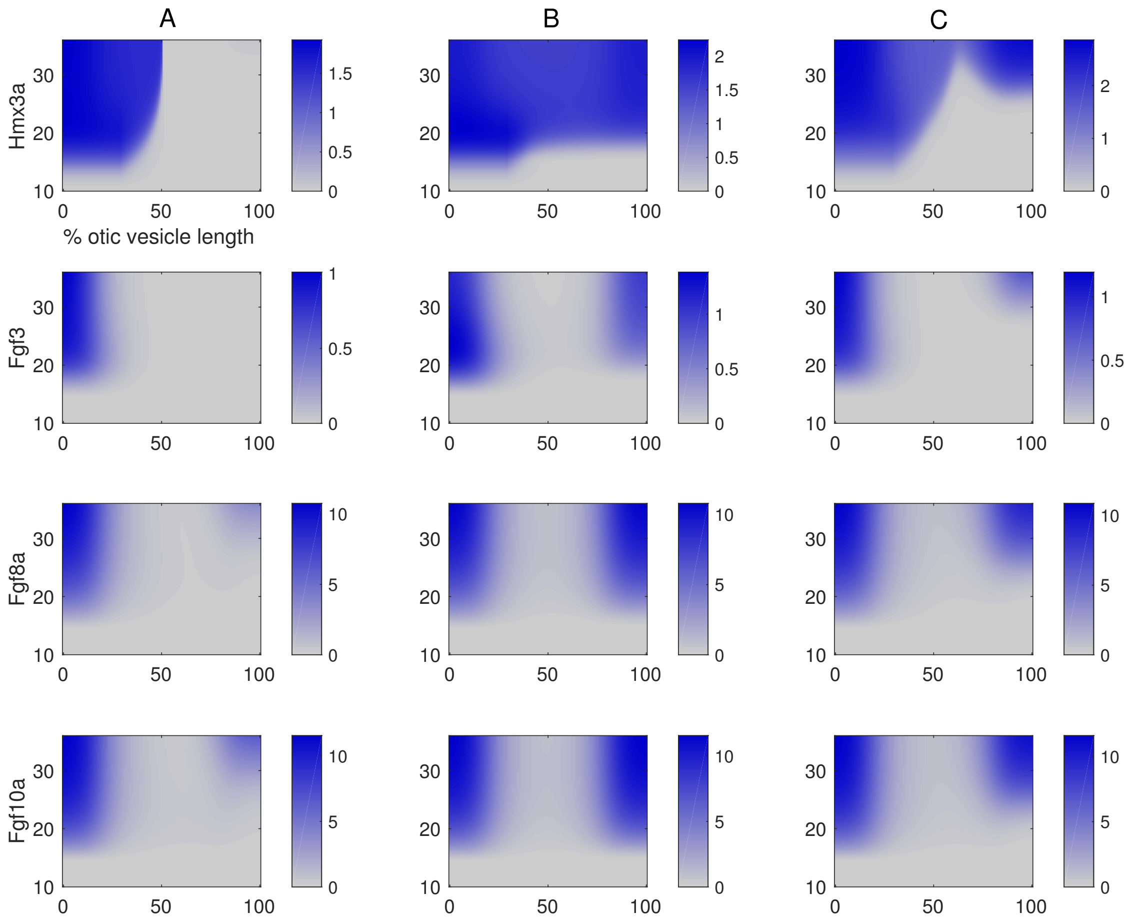

Figure S3: Spatio-temporal expression profiles of Hmx3a, Fgf3, Fgf8a and Fgf10a protein model variables in simulations of wild type (column A), heat shock induction of *fgf3* (column B), and cyclopamine treatment (column C).

Parameter	*Value*	*Description*
θ_1	10	Fgf–*hmx3a* threshold
θ_2	5	Fgf–*pax5* threshold
θ_3	0.68	Hmx3a–*pax5* threshold
θ_4	25	Fgf–*fgf3* threshold
θ_5	0.83	Hmx3a–*fgf3* threshold
θ_6	1	Fgf–*fgf8a* threshold
θ_7	0.06	Hmx3a–*fgf8a* threshold
θ_8	1	Fgf–*fgf10a* threshold
θ_9	0.04	Hmx3a–*fgf10a* threshold
θ_{10}	0.25	Hmx3a–*hmx3a* threshold
ρ_1	2	Hh–*hmx3a* attenuation coefficient
ρ_2	0.4	Hh–*pax5* attenuation coefficient
ρ_4	0.1	Hh–*fgf3* attenuation coefficient
ρ_6	0.1	Hh–*fgf8a* attenuation coefficient
ρ_8	0.05	Hh–*fgf10a* attenuation coefficient
β_7	1	Hmx3a-independent *fgf8a* production coefficient
β_9	2	Hmx3a-independent *fgf10a* production coefficient

Table S3: Transcription regulation parameters

Simulation of heat shock induction of *fgf3a* expression and cyclopamine treatment

To simulate heat shock induction of *fgf3* expression, we include additional variables to represent *fgf3* mRNA and Fgf3 protein produced from the *fgf3* transgene. We assume that transcription from the transgene starts at 14 hpf and terminates at 14.5 hpf. Because of the time taken for the resulting mRNA and protein to decay, the effects of the heat shock on target gene expression extend beyond 14.5 hpf.

To simulate treatment of embryos with cyclopamine (a chemical inhibitor of Hh signalling) at 14 hpf, we assume that a consequent reduction in the Hh-dependent antagonism of Fgf-dependent transcription (the variable R in the model equations) does not begin until 15 hpf. In this way, we represent the time taken for a reduction in Hh signalling to feed through to a reduction in the intracellular effectors of Hh signalling. We further assume that the inhibitory term R decays exponentially for $t > 15$ hpf, with a half-life of 3 hrs.

References

Kwak, S.-J., Phillips, B. T., Heck, R. and Riley, B. B. (2002). An expanded domain of *fgf3* expression in the hindbrain of zebrafish *valentino* mutants results in mis-patterning of the otic vesicle. *Development* **129**, 5279–5287.

Lecaudey, V., Ulloa, E., Anselme, I., Stedman, A., Schneider-Maunoury, S. and Pujades, C. (2007). Role of the hindbrain in patterning the otic vesicle: A study of the zebrafish *vhnf1* mutant. *Dev. Biol.* **303**, 134–143.

Maves, L., Jackman, W. and Kimmel, C. B. (2002). FGF3 and FGF8 mediate a rhombomere 4 signaling activity in the zebrafish hindbrain. *Development* **129**, 3825–3837.
